# Supplementary material for: Is personalised prehabilitation feasible to implement for patients undergoing oncological treatment for lung cancer at a London teaching hospital? Protocol of a feasibility trial
Source: BMJ Open. 2023 Jul 17;13(7):e072367. doi: 10.1136/bmjopen-2023-072367 (PMC10357652; doi:10.1136/bmjopen-2023-072367)
Supplement: Supplementary data [file bmjopen-2023-072367supp001.pdf]

Co-design approach used for the development of the personalised prehabilitation programme.

A participatory action research approach using qualitative group interviews to develop the intervention, as guided by the MRC framework, was undertaken in partnership with a single London teaching hospital and Maggie's cancer centre (an independent charity that provides free cancer support, workshops and courses to help anyone affected by cancer).

Participants who had completed oncological treatment for lung cancer were invited to an informal face-to-face group discussion. Participants were invited either in person at the monthly lung cancer support group held at Maggie's cancer centre or by their clinical oncologist or clinical nurse specialist at a follow up outpatient appointment.

Nine participants were invited to the informal group discussion and a total of six participants attended. The main reason for non-attendance was a lack of time. Participants were invited to share their thoughts on what they would want from a prehabilitation programme and through facilitated discussion, participant priorities were agreed. Many participants remarked on the importance of individualisation, being seen face-to-face and the programme being endorsed by all members of the team.

Separately, five healthcare professionals from a range of different specialties including clinical psychology, physiotherapy, dietetics and cancer services met to discuss their ideas about what a prehabilitation programme for people with lung cancer undergoing chemotherapy or radiotherapy treatment should look like.

A joint meeting was then held with both participants and healthcare professionals to share thoughts and ideas and an agreement was sought on what an ideal prehabilitation programme should look like.
