# Supplementary material for: Is personalised prehabilitation feasible to implement for patients undergoing oncological treatment for lung cancer at a London teaching hospital? Protocol of a feasibility trial
Source: BMJ Open. 2023 Jul 17;13(7):e072367. doi: 10.1136/bmjopen-2023-072367 (PMC10357652; doi:10.1136/bmjopen-2023-072367)
Supplement: Author's manuscript [file bmjopen-2023-072367.draft_revisions.pdf]

BMJ Open is committed to open peer review. As part of this commitment we make the peer review history of every article we publish publicly available.

When an article is published we post the peer reviewers' comments and the authors' responses online. We also post the versions of the paper that were used during peer review. These are the versions that the peer review comments apply to.

The versions of the paper that follow are the versions that were submitted during the peer review process. They are not the versions of record or the final published versions. They should not be cited or distributed as the published version of this manuscript.

BMJ Open is an open access journal and the full, final, typeset and author-corrected version of record of the manuscript is available on our site with no access controls, subscription charges or pay-per-view fees (http://bmjopen.bmj.com).

If you have any questions on BMJ Open's open peer review process please email info.bmjopen@bmj.com

BMJ Open

Personalised prehabilitation for patients undergoing oncological treatment for lung cancer. Protocol of a feasibility study.

Journal:	*BMJ Open*
Manuscript ID	bmjopen-2023-072367
Article Type:	Protocol
Date Submitted by the Author:	01-Feb-2023
Complete List of Authors:	Wade-Mcbane, Kelly; Imperial College Healthcare NHS Trust<br>King, Alex; Imperial College Healthcare NHS Trust<br>Urch, Catherine; Imperial College Healthcare NHS Trust<br>Johansson, Lina; Imperial College Healthcare NHS Trust<br>Wells, Mary; Imperial College Healthcare NHS Trust
Keywords:	CHEMOTHERAPY, RADIOTHERAPY, Respiratory tract tumours < ONCOLOGY

SCHOLARONE™
Manuscripts

Personalised prehabilitation for patients undergoing oncological treatment for lung cancer. Protocol of a feasibility study.

Kelly Wade-Mcbane[1], Alex King[1], Catherine Urch[1], Lina Johansson[1], Mary Wells[1]

[1]Imperial College Healthcare NHS Trust, London, UK

Correspondence: Kelly Wade-Mcbane, Nutrition and Dietetics Department, Charing Cross Hospital, Imperial College Healthcare NHS Trust, London, UK. Email: k.wade-mcbane@nhs.net

Abstract

Introduction
There is significant potential to improve outcomes for patients with lung cancer in terms of quality of life and survival. There is some evidence that prehabilitation can help, but, to date, this has only been tested in surgical populations, despite 70-80% of lung cancer patients in the UK receiving non-surgical treatment. The physiological and psychological benefits of prehabilitation seen in surgical patients could be extrapolated to those receiving non-surgical treatment, particularly in such a poor prognosis group.
With patients and healthcare professionals, we have co-designed a personalised and evidence-based prehabilitation programme. This draws on a conceptual framework that aligns with patient values and needs as well as functional goals. We aim to investigate whether this programme is feasible to implement and evaluate in clinical practice.

Methods and analysis
A feasibility study incorporating quantitative assessments, a qualitative free text questionnaire and reflective field notes. All recruited participants will receive a personalised prehabilitation programme during their oncological treatment. This includes a one-hour face-to-face appointment prior to, at week three and at week six of their treatment regimen as well as a weekly telephone call. Interventions including nutrition, physical activity and psychological wellbeing are stratified according to a patient's priorities, level of readiness and expressed needs.

Primary outcome
Feasibility of the personalised prehabilitation programme in clinical practice by investigating areas of uncertainty regarding patient recruitment, attrition, treatment fidelity, intervention adherence and acceptability of study outcome measures.
Secondary outcomes: quality of life, functional capacity and grip strength.

Ethics and dissemination
Ethical approval has been obtained from the Health Research Authority (HRA) (reference number 22/PR/0390). Results of this study will be disseminated through publication in peer-reviewed articles, presentations at scientific conferences and in collaboration with patient and public involvement representatives.

Keywords: prehabilitation, physical activity, nutrition, wellbeing, chemotherapy, radiotherapy, immunotherapy, conceptual framework, psychological adjustment theory

For peer review only - http://bmjopen.bmj.com/site/about/guidelines.xhtml

Strengths and limitations of this study

- A novel way of delivering a multi-modal prehabilitation programme using a personalised care approach that aligns with patient values and needs as well as functional goals.
- Scarce evidence on the feasibility of prehabilitation in lung cancer patients receiving chemotherapy, radiotherapy or immunotherapy treatment.
- Risk of potential bias owing the lead researcher also being the clinician delivering the interventions.

Introduction

Multi-modal prehabilitation programmes incorporating nutrition, physical activity and psychological wellbeing are increasingly being used in surgical cancer pathways. This is in order to optimise health outcomes and enhance recovery by reducing post-operative complications (1–5) and hospital length of stay (4,6–8) as well as improving health reported quality of life (9–12). Few prehabilitation pathways exist for patients who do not have surgery, despite the fact that 50-60% of patients with cancer in the UK are treated with primary, neo-adjuvant or palliative chemotherapy and/or radiotherapy treatment (13).

Only a minority of patients with lung cancer are eligible for surgery, with 70-80% of lung cancer patients in the UK receiving non-surgical treatment (14) without any prehabilitation. UK deaths from lung cancer are higher than any other cancer and treatment outcomes are poor (15). There is significant potential to improve outcomes for patients with lung cancer and there is some evidence that prehabilitation can help. However, this has only been tested in surgical populations. A small number of studies have shown that prehabilitation before lung cancer surgery is feasible and can improve functional capacity and reduce post-operative complications. However, most of these studies focused on the benefits of exercise prehabilitation as opposed to a multi-modal approach (16–25).

Prehabilitation during chemotherapy, radiotherapy or immunotherapy treatment for lung cancer poses significant challenges. Treatment regimes are often prolonged and patients may experience a range of toxicities, which could limit their ability to engage in prehabilitation interventions. Personalisation to patient-led values and needs is therefore important, as initiation and adherence to any intervention is determined by behavioural, psychological, physiological, environmental and social factors (26–28).

We have co-designed a multi-modal personalised prehabilitation programme with healthcare professionals and patients. This co-design process is illustrated in figure one. The multi-modal structure of the programme is informed by the Macmillan, Royal College of Anaesthetists and National Institute of Health Cancer and Nutrition Collaboration, Research Principles and Guidance for Prehabilitation within the Management and Support of People with Cancer (29). This guidance incorporates nutrition, physical activity and psychological wellbeing using a tiered approach, tailored to whether patients need universal (applicable to anyone with cancer), targeted (applicable to those at risk of acute, chronic or latent effects of disease or treatment) or specialist (applicable to those with complex needs, severe impairment and/or disability) intervention (29). The first level of intervention – universal – comprises of written information and relevant signposting to support resources for all patients. If psychological, nutrition and/or physical activity needs are identified, patients then

For peer review only - http://bmjopen.bmj.com/site/about/guidelines.xhtml

receive the appropriate intervention (targeted or specialist) according to their level of need and readiness.

The personalised prehabilitation programme uses the Adversity, Restoration and Compatibility (ARC) framework (27) to underpin a tailored and collaborative approach. The ARC framework provides a synthesised view of how people conceptualise the personal experience of living with and beyond cancer. Consistent with psychological adjustment theory (30), it frames this experience as an ongoing process of learning about the evolving challenge (Adversity), learning how to cope effectively (Restoration) and adapting one's identity (Compatibility), in parallel (see figure two). Our programme provides a structure and process for personalising prehabilitation, which is patient-centred rather than providing a purely logistical or psychometric approach. As recommend by Hickmann et al (31), such an approach could be key to empower patients to maintain progress through the prolonged, more variable context of peri-oncological prehabilitation.

This proposed feasibility study aims to investigate whether a personalised prehabilitation programme co-designed with healthcare professionals and patients and informed by theory and evidence is feasible to implement and evaluate in clinical practice.

Methods and analysis

Feasibility studies are particularly relevant to assess whether a future definitive study can or should be done (32,33). Using the Orsmond & Cohn framework for feasibility studies (34), this study will use quantitative assessments, a qualitative free text questionnaire and reflective field notes to investigate areas of uncertainty regarding patient recruitment, attrition, treatment fidelity, intervention adherence and acceptability of study outcome measures. This will help inform if the personalised prehabilitation programme can be implemented and evaluated in clinical practice and will provide data to estimate the parameters required to design a future definitive study.

Eligibility criteria

Adults (≥18 years) with a diagnosis of lung cancer who are due to commence chemotherapy, radiotherapy or immunotherapy treatment.
Participants who are due to undergo lung cancer surgery, have had previous lung cancer surgery or unable to give informed consent will not be eligible for this study.

Recruitment

Participants will be recruited from the study hospital's lung cancer clinic via referral from the Lung Cancer Multi-Disciplinary Team (LCMDT). Recruitment will continue until 30 participants have been recruited or until six months have elapsed. A pragmatic sample size of 30 participants over a six-month recruitment period has been selected, which is feasible within the time frame of this study. This is based on 144 - 240 new lung cancer diagnoses per year at the study hospital, with an assumption of a 50% uptake over a six-month period. Furthermore, it is in accordance with published guidance on recommended sample sizes for feasibility studies (35,36).

For peer review only - http://bmjopen.bmj.com/site/about/guidelines.xhtml

The personalised prehabilitation programme

All participants will be required to attend three face-to-face appointments at the study hospital for study assessments, but the interventions will be delivered using a hybrid approach; either face-to-face at the study hospital or remotely via telephone or video conferencing depending on participant preference.

The three face-to-face sessions with the lead researcher will be prior to, at week three and at week six of a participant's oncological treatment regimen.
Following consent, participants will be guided through a set of questionnaires (as detailed below) to complete prior to their first face-to-face appointment. These questionnaires will be reviewed during the initial session, enabling the nutrition, physical activity and/or psychological wellbeing interventions to be stratified according to a participant's priorities, level of readiness and expressed needs:

- Cancer Behaviour Inventory (CBI) brief form (37) - to measure self-efficacy for coping with cancer
- Patient Activation Measure (PAM) (38) - to measure a person's knowledge, skills and confidence in managing their own health and wellbeing
- Modified Godin leisure time exercise questionnaire (39) - to measure activity
- Patient Generated Subjective Global Assessment (PG-SGA) short form (40) - to identify malnutrition risk
- Emotions Thermometer (ET) (41) - to evaluate levels of emotional distress
- Patient Generated Index (PGI) (42) – to identify participant priorities

Questionnaire responses will inform the conversation with the participant about their priorities and enable interventions to be stratified according to the participant's level of readiness and need, as illustrated in figures 3a, 3b and 3c.

The personalised prehabilitation programme uses a universal, targeted and specialist approach to prehabilitation interventions, in line with current guidance (29). All participants will receive a universal intervention comprising of written information and relevant signposting to cancer specific nutrition, physical activity and/or psychosocial wellbeing support resources. If a participant identifies a psychological, nutrition and/or physical activity need, they will then receive the appropriate intervention according to their level of need and readiness (see figures 3a, 3b and 3c). Targeted interventions will be delivered via the use of certified apps and/or specific group activities at Maggie's cancer centre (an independent charity that provides free cancer support, workshops and courses to help anyone affected by cancer). For example, an exercise class for participants undergoing treatment, nutritional education sessions as well as a lung cancer support group. These group activities are run by cancer support workers or qualified instructors. Specialist interventions involve a 1:1 assessment with a registered healthcare professional such as a physiotherapist, dietitian and/or a clinical psychologist depending on a participant's needs.

During each of the face-to-face sessions, participants will also be asked to (a) undertake a six-minute walk test (6MWT) to assess functional capacity and (b) measure their grip strength using a handgrip dynamometer to assess upper body strength. These functional

For peer review only - http://bmjopen.bmj.com/site/about/guidelines.xhtml

measurements will be used to assess a participant's level of need to help tailor the intervention (see figures 3a, 3b and 3c).

Owing to the prolonged nature of chemotherapy, radiotherapy or immunotherapy treatment in contrast to a defined one-off target such as surgery, patient-led values and needs are likely to be more variable within the context of peri-oncological prehabilitation. Throughout treatment, participants will be asked to keep a daily diary to record their symptoms, appetite, mobility and level of distress each day using a Likert scale. This will provide participants with a visual representation of their progress and help identify any adverse trends.
Participants will also receive a weekly telephone call from the lead researcher (a specialist oncology dietitian). Participants will be able to use the diary to highlight any days they found particularly good or difficult and have the opportunity to discuss these. Personalisation is central to this study and the language used during the face-to-face appointments and the weekly telephone calls has validation and compassion at its core to help build self-efficacy and sustain behaviour change (43). The use of the PGI to understand personal priorities is fundamental to this personalised approach. A systematic review (44) demonstrated the large potential value of using the PGI by enabling patients with cancer to participate in the decision-making process. This is key to empower patients to take ownership of their own health and for the sustainability of long-term positive behaviour change and self-care.

Primary objective and outcome measure
The primary objective is to assess the feasibility of the personalised prehabilitation programme in clinical practice by gathering data on how accessible and relevant it is for lung cancer patients undergoing chemotherapy, radiotherapy or immunotherapy treatment. This will be addressed using the research objectives and methods of assessment outlined in table 1 below.

Table 1. Research objectives and methods of assessment to investigate the feasibility of the personalised prehabilitation programme in clinical practice.

Research objective	Method of assessment(s)
To determine how many participants participate in the personalised prehabilitation programme	Descriptive statistics – total number of participants eligible, approached and taking part
To determine how many participants do not participate in the personalised prehabilitation programme and explore the reasons why	Descriptive statistics<br>Field notes taken during the Lung Cancer Multi-Disciplinary Team (LCMDT)<br>Participant discussion
To assess who is not suitable for the personalised prehabilitation programme and the reasons why	Field notes taken during the LCMDT, where discussions of eligibility will take place
To estimate the feasible eligibility, recruitment and refusal rates	Descriptive statistics – number approached, eligible, recruited, refused
To assess attrition rate throughout the personalised prehabilitation programme	Descriptive statistics – number who drop out and at what stage
To assess adherence to the personalised prehabilitation interventions	Weekly telephone call – adherence to goals set
To assess the barriers and facilitators to adherence and intervention fidelity	Daily diary – symptom log<br>Weekly telephone call

For peer review only - http://bmjopen.bmj.com/site/about/guidelines.xhtml

	Short free text questionnaire at the end of the study, analysed thematically
To assess if study processes and outcome measures are feasible and acceptable to participants	Completion rates<br>Weekly telephone call<br>Short free text questionnaire at the end of the study, analysed thematically
To evaluate contextual or implementation factors which may influence acceptability or effectiveness of the intervention/s	Daily diary – symptom log<br>Weekly telephone call – participant reflection, needs and priorities<br>Short free text questionnaire at the end of the study, analysed thematically
To determine the 'prehabilitation window' (length of time between diagnosis and the start of treatment)	Descriptive statistics – date of diagnosis, date of start of treatment, date of start and finish of prehabilitation
To determine the extent to which the intervention/s are likely to have an impact on psychological wellbeing, nutrition and/or physical activity and quality of life	Questionnaires – change between baseline and follow up<br>Daily diary – symptom log<br>Weekly telephone call<br>Short free text questionnaire at the end of the study, analysed thematically
To determine the number of participants who require universal, targeted and specialist intervention	Questionnaires – level of need and readiness<br>Descriptive statistics

Secondary objectives and outcome measures

The benefits of prehabilitation can be seen in as little as two weeks (29). The secondary objectives will assess the impact of the personalised prehabilitation programme on:

- Overall quality of life as measured by:
  - European Organisation for Research and Treatment of Cancer (EORTC) QLQ-C30; a cancer specific assessment of quality of life, sensitive to changes over time (45)
  - EuroQOL group EQ-5D-5L for a descriptive overview of quality of life, which can be used for economic evaluation (46).
- Functional capacity as measured by the six-minute walk test (6MWT) (47). The 6MWT is a safe, valid and reliable measure of functional capacity in cancer populations (48). All participants will perform a standardised, self-paced 6MWT in a 30-metre long corridor. Each participant will be instructed to walk as far as they can for six minutes. Participants will be allowed to stop at any time. The distance covered (to the nearest metre) after the six minutes will be measured.
- Handgrip strength as measured by a handgrip dynamometer (49). Grip strength has shown to be correlated with overall health status (50–54). Each participant will be instructed to sit in a chair and bend the elbow of their non-dominant arm at a 90-degree angle. The lead researcher will ask the participant to squeeze the device as hard as they can. Each participant will repeat this three times and the average will be recorded.

To tailor the intervention, the above outcome measures will be assessed prior to starting treatment and before the personalised prehabilitation programme begins (baseline), at week three and at week six of a participant's oncological treatment (end point).

Demographic and clinical data including age, gender, level of education achieved, ethnicity, employment status, level of social support (measured using the Duke-University of North

For peer review only - http://bmjopen.bmj.com/site/about/guidelines.xhtml

Carolina Functional Social Support Questionnaire; DUFSSQ) (55), diagnosis, staging, treatment and co-morbidities (measured using the Adult Co-morbidity Evaluation (ACE) 27 score) (56) will be collected at baseline.

Training

The lead researcher is a highly specialised oncology dietitian trained in advanced communication and psychological skills (57) to manage complex conversations with patients and help recognise and respond to psychological distress. The lead researcher is familiar with using a handgrip dynamometer from previous experience in clinical practice. Training in conducting the 6MWT has been provided by a specialist oncology physiotherapist.

Patient and public involvement (PPI)

Four patients (and a spouse) who had recently completed chemotherapy or radiotherapy treatment for lung cancer were consulted on the personalised prehabilitation programme via a focus group. This was separate to focus groups used in the initial co-design process to develop the personalised prehabilitation programme (see figure one). Members of the focus group were very positive about the programme, with many patients reporting that they found having a strong mind-set, staying active and eating well helped them get through treatment. These are key components of the personalised prehabilitation programme. The focus group also reviewed the study questionnaires and patient documentation. Patients and their relatives expressed the benefits of having a weekly telephone call to discuss their concerns and reported that the questionnaires and daily diary would make them feel supported, as the information would be tailored to them individually.

All participants who participate in the study will be offered the opportunity to provide feedback on their experience of the personalised prehabilitation programme via the use of a short free text questionnaire. This feedback will help inform future programme adjustments and have the potential to improve patient experience.

Data collection and management

Data will be collected and stored according to the Data Protection Act (2018) and in line with General Data Protection Regulation (GDPR).

All data included in this study will be kept as part of the participants clinical care record on a secure password protected electronic patient record system at the study hospital.

A subset of this data will be extracted and entered into a case report form, which will be kept on a secure password protected database at the study hospital and only accessed by the research team. This data will be pseudonymised and all participants will be given a unique study number.

Statistical analysis plan

This study includes a small cohort of lung cancer patients from a single London teaching hospital. There are no groups to detect differences between, therefore, no formal tests of statistical significance will be undertaken.

Descriptive statistics will be used to analyse baseline demographic and clinical data.

Continuous measures will be reported as means, standard deviations and/or medians, while categorical data will be reported as percentages. Missing data will be reported where applicable.

Inductive thematic analysis will be used to analyse qualitative data from the short free text questionnaire regarding patient experience.

For peer review only - http://bmjopen.bmj.com/site/about/guidelines.xhtml

The data collected will be used to inform the design of a larger randomised pilot study.

Strengths and limitations

This study uses a novel way of delivering a multi-modal prehabilitation programme using a personalised care approach that aligns with patient values and needs as well as functional goals. The personalised prehabilitation programme provides a structure and process for personalising prehabilitation that is patient-centred and underpinned by a conceptual framework, which is consistent with psychological adjustment theory. This could be key to empower patients to maintain activity through the longer, more variable context of peri-oncological prehabilitation.

This study will help address key uncertainties, which need to be resolved before implementation, including engagement and adherence to prehabilitation interventions in lung cancer patients receiving chemotherapy, radiotherapy or immunotherapy treatment.

This study is limited by (a) lack of a comparison group (b) the small sample size used and (c) the lead researcher also being the clinician delivering the interventions. In order to mitigate potential bias regarding the latter, where possible, aspects of the programme have been pre-defined.

Ethics and dissemination

Ethical approval was obtained from the Health Research Authority (HRA) on 13th April 2022 (reference number 22/PR/0390) prior to the start of the study. Any modifications will be approved by the HRA. This study has been registered with ClinicalTrials.gov. Imperial College Healthcare NHS Trust is the sponsor of the study. Monitoring and auditing will be conducted in accordance with the sponsor’s policies and procedures. Results of this study will be disseminated to academics, stakeholders, cancer alliances, relevant charities and to the public and people with cancer. This will be through publication in peer-reviewed articles, presentations at scientific conferences, social media and collaboration with patient and public involvement representatives.

Author contributions

KWM is the principal investigator. KWM devised the clinical question, applied for funding and research ethics and was involved in the conception and design of the study and protocol. AK contributed to the study design and methods. AK and CU were involved in the conception and design of the study. LJ and MW contributed to all versions of the protocol. AK, CU and MW provided their expertise in cancer care and contributed to the knowledge needed for prehabilitation. CU, LJ and MW provided supervision and mentorship. All authors read and approved the final protocol.

Funding

This work was supported by Health Education England and the National Institute for Health and Care Research as part of an ICA Pre-doctoral Clinical and Practitioner Academic Fellowship (Grant award number: NIHR302680).

For peer review only - http://bmjopen.bmj.com/site/about/guidelines.xhtml

Competing interests

The authors declare that they have no competing interests.

References

1. Berkel AEM, Bongers BC, van Kamp MJS, Kotte H, Weltevreden P, de Jongh FHC, et al. The effects of prehabilitation versus usual care to reduce postoperative complications in high-risk patients with colorectal cancer or dysplasia scheduled for elective colorectal resection: study protocol of a randomized controlled trial. BMC Gastroenterol. 2018 Dec;18(1):29.

2. van Rooijen S, Carli F, Dalton S, Thomas G, Bojesen R, Le Guen M, et al. Multimodal prehabilitation in colorectal cancer patients to improve functional capacity and reduce postoperative complications: the first international randomized controlled trial for multimodal prehabilitation. BMC Cancer. 2019 Dec;19(1):98.

3. Moore J, Merchant Z, Rowlinson K, McEwan K, Evison M, Faulkner G, et al. Implementing a system-wide cancer prehabilitation programme: The journey of Greater Manchester's 'Prehab4cancer.' Eur J Surg Oncol. 2021 Mar;47(3):524–32.

4. Perry R, Herbert G, Atkinson C, England C, Northstone K, Baos S, et al. Pre-admission interventions (prehabilitation) to improve outcome after major elective surgery: a systematic review and meta-analysis. BMJ Open. 2021 Sep;11(9):e050806.

5. Molenaar CJ, van Rooijen SJ, Fokkenrood HJ, Roumen RM, Janssen L, Slooter GD. Prehabilitation versus no prehabilitation to improve functional capacity, reduce postoperative complications and improve quality of life in colorectal cancer surgery. Cochrane Colorectal Group, editor. Cochrane Database Syst Rev [Internet]. 2022 May 19 [cited 2023 Jan 26];2022(5). Available from: http://doi.wiley.com/10.1002/14651858.CD013259.pub2

6. Gillis C, Buhler K, Bresee L, Carli F, Gramlich L, Culos-Reed N, et al. Effects of Nutritional Prehabilitation, With and Without Exercise, on Outcomes of Patients Who Undergo Colorectal Surgery: A Systematic Review and Meta-analysis. Gastroenterology. 2018 Aug;155(2):391-410.e4.

7. Lambert JE, Hayes LD, Keegan TJ, Subar DA, Gaffney CJ. The Impact of Prehabilitation on Patient Outcomes in Hepatobiliary, Colorectal, and Upper Gastrointestinal Cancer Surgery: A PRISMA-Accordant Meta-analysis. Ann Surg. 2021 Jul;274(1):70–7.

8. Waterland JL, McCourt O, Edbrooke L, Granger CL, Ismail H, Riedel B, et al. Efficacy of Prehabilitation Including Exercise on Postoperative Outcomes Following Abdominal Cancer Surgery: A Systematic Review and Meta-Analysis. Front Surg. 2021 Mar 19;8:628848.

9. Treanor C, Kyaw T, Donnelly M. An international review and meta-analysis of prehabilitation compared to usual care for cancer patients. J Cancer Surviv. 2018 Feb;12(1):64–73.

For peer review only - http://bmjopen.bmj.com/site/about/guidelines.xhtml

10. Fulop A, Lakatos L, Susztak N, Szijarto A, Banky B. The effect of trimodal prehabilitation on the physical and psychological health of patients undergoing colorectal surgery: a randomised clinical trial. Anaesthesia. 2021 Jan;76(1):82–90.

11. Wu F, Rotimi O, Laza-Cagigas R, Rampal T. The Feasibility and Effects of a Telehealth-Delivered Home-Based Prehabilitation Program for Cancer Patients during the Pandemic. Curr Oncol. 2021 Jun 17;28(3):2248–59.

12. Allen SK, Brown V, White D, King D, Hunt J, Wainwright J, et al. Multimodal Prehabilitation During Neoadjuvant Therapy Prior to Esophagogastric Cancer Resection: Effect on Cardiopulmonary Exercise Test Performance, Muscle Mass and Quality of Life—A Pilot Randomized Clinical Trial. Ann Surg Oncol. 2022 Mar;29(3):1839–50.

13. Cancer Research UK. Cancer Research UK. Cancer treatment statistics 2013-2016 [Internet]. 2013 2016 [cited 2022 Oct 3]. Available from: https://www.cancerresearchuk.org/health-professional/cancer-statistics/treatment

14. Cancer Research UK. Cancer Research UK. Lung cancer treatment statistics 2013-2014 [Internet]. 2013 2014 [cited 2022 Oct 17]. Available from: https://www.cancerresearchuk.org/health-professional/cancer-statistics/statistics-by-cancer-type/lung-cancer/diagnosis-and-treatment#heading-Three

15. Cancer Research UK. Cancer Research UK. Lung cancer mortality statistics 2017-2019 [Internet]. 2017 2019 [cited 2022 Oct 17]. Available from: https://www.cancerresearchuk.org/health-professional/cancer-statistics/statistics-by-cancer-type/lung-cancer#heading-Five

16. Laurent H, Aubreton S, Galvaing G, Pereira B, Merle P, Richard R, et al. Preoperative respiratory muscle endurance training improves ventilatory capacity and prevents pulmonary postoperative complications after lung surgery. Eur J Phys Rehabil Med [Internet]. 2020 Feb [cited 2022 Oct 17];56(1). Available from: https://www.minervamedica.it/index2.php?show=R33Y2020N01A0073

17. Liu Z, Qiu T, Pei L, Zhang Y, Xu L, Cui Y, et al. Two-Week Multimodal Prehabilitation Program Improves Perioperative Functional Capability in Patients Undergoing Thoracoscopic Lobectomy for Lung Cancer: A Randomized Controlled Trial. Anesth Analg. 2020 Sep;131(3):840–9.

18. Ricketts WM, Bollard K, Streets E, Hutton K, Hornby C, Lau K. Feasibility of setting up a pre-operative optimisation 'pre-hab' service for lung cancer surgery in the UK. Perioper Med. 2020 Dec;9(1):14.

19. Avancini A, Cavallo A, Trestini I, Tregnago D, Belluomini L, Crisafulli E, et al. Exercise prehabilitation in lung cancer: Getting stronger to recover faster. Eur J Surg Oncol. 2021 Aug;47(8):1847–55.

20. Bradley P, Merchant Z, Rowlinson-Groves K, Grundy S, Al-Najjar H, Brown L, et al. P212 Prehab4Cancer: an innovative regional lung cancer prehabilitation service. In: The lung cancer diagnostic journey [Internet]. BMJ Publishing Group Ltd and British Thoracic Society; 2021 [cited 2022 Oct 17]. p. A204.2-A205. Available from: https://thorax.bmj.com/lookup/doi/10.1136/thorax-2020-BTSabstracts.357

21. Ferreira V, Minnella EM, Awasthi R, Gamsa A, Ferri L, Mulder D, et al. Multimodal Prehabilitation for Lung Cancer Surgery: A Randomized Controlled Trial. Ann Thorac Surg. 2021 Nov;112(5):1600–8.

For peer review only - http://bmjopen.bmj.com/site/about/guidelines.xhtml

22. Goldsmith I, Chesterfield-Thomas G, Toghill H. Pre-treatment optimization with pulmonary rehabilitation in lung cancer: Making the inoperable patients operable. EClinicalMedicine. 2021 Jan;31:100663.

23. Bradley P, Merchant Z, Rowlinson-Groves K, Taylor M, Moore J, Evison M. Feasibility and outcomes of a real-world regional lung cancer prehabilitation programme in the UK. Br J Anaesth. 2022 Jul;S0007091222002951.

24. Granger C, Cavalheri V. Preoperative exercise training for people with non-small cell lung cancer. Cochrane Lung Cancer Group, editor. Cochrane Database Syst Rev [Internet]. 2022 Sep 28 [cited 2023 Jan 11];2022(9). Available from: http://doi.wiley.com/10.1002/14651858.CD012020.pub3

25. Schmid S, Minnella EM, Pilon Y, Rokah M, Rayes R, Najmeh S, et al. Neoadjuvant Prehabilitation Therapy for Locally Advanced Non–Small-Cell Lung Cancer: Optimizing Outcomes Throughout the Trajectory of Care. Clin Lung Cancer. 2022 Nov;23(7):593–9.

26. Ferreira V, Agnihotram RV, Bergdahl A, van Rooijen SJ, Awasthi R, Carli F, et al. Maximizing patient adherence to prehabilitation: what do the patients say? Support Care Cancer. 2018 Aug;26(8):2717–23.

27. Boutillier CL, Archer S, Barry C, King A, Mansfield L, Urch C. Conceptual framework for living with and beyond cancer: A systematic review and narrative synthesis. Psychooncology. 2019;28(5):948–59.

28. Grimmett C, Bradbury K, Dalton SO, Fecher-Jones I, Hoedjes M, Varkonyi-Sepp J, et al. The Role of Behavioral Science in Personalized Multimodal Prehabilitation in Cancer. Front Psychol. 2021;12:261.

29. Macmillan Cancer Support, NIHR Cancer and Nutrition Collaboration, Royal College of Anaesthetists. Principles and guidance for prehabilitation within the management and support of people with cancer [Internet]. Macmillan Cancer Support; 2019 [cited 2021 Oct 27]. Available from: https://www.macmillan.org.uk/healthcare-professionals/news-and-resources/guides/principles-and-guidance-for-prehabilitation

30. Brennan J. Adjustment to cancer?coping or personal transition? Psychooncology. 2001 Jan;10(1):1–18.

31. Hickmann E, Richter P, Schlieter H. All together now – patient engagement, patient empowerment, and associated terms in personal healthcare. BMC Health Serv Res. 2022 Sep 2;22(1):1116.

32. Eldridge SM, Lancaster GA, Campbell MJ, Thabane L, Hopewell S, Coleman CL, et al. Defining Feasibility and Pilot Studies in Preparation for Randomised Controlled Trials: Development of a Conceptual Framework. Lazzeri C, editor. PLOS ONE. 2016 Mar 15;11(3):e0150205.

33. on behalf of the PAFS consensus group, Eldridge SM, Chan CL, Campbell MJ, Bond CM, Hopewell S, et al. CONSORT 2010 statement: extension to randomised pilot and feasibility trials. Pilot Feasibility Stud. 2016 Dec;2(1):64, s40814-016-0105–8.

34. Orsmond GI, Cohn ES. The Distinctive Features of a Feasibility Study: Objectives and Guiding Questions. OTJR Occup Particip Health. 2015 Jul;35(3):169–77.

For peer review only - http://bmjopen.bmj.com/site/about/guidelines.xhtml

35. Julious SA. Sample size of 12 per group rule of thumb for a pilot study. Pharm Stat. 2005 Oct;4(4):287–91.

36. Sim J, Lewis M. The size of a pilot study for a clinical trial should be calculated in relation to considerations of precision and efficiency. J Clin Epidemiol. 2012 Mar;65(3):301–8.

37. Heitzmann CA, Merluzzi TV, Jean-Pierre P, Roscoe JA, Kirsh KL, Passik SD. Assessing self-efficacy for coping with cancer: development and psychometric analysis of the brief version of the Cancer Behavior Inventory (CBI-B). Psychooncology. 2011 Mar;20(3):302–12.

38. Hibbard JH, Mahoney ER, Stockard J, Tusler M. Development and Testing of a Short Form of the Patient Activation Measure. Health Serv Res. 2005 Dec;40(6p1):1918–30.

39. Godin G. The Godin-Shephard Leisure-Time Physical Activity Questionnaire. Health Fit J Can. 2011 Jan 30;18-22 Pages.

40. Ottery F. Definition of standardized nutritional assessment and interventional pathways in oncology. Nutrition. 1996 Jan;12:S15–9.

41. Harju E, Michel G, Roser K. A systematic review on the use of the emotion thermometer in individuals diagnosed with cancer. Psychooncology. 2019 Sep;28(9):1803–18.

42. Ruta DA, Garratt AM, Leng M, Russell IT, MacDonald LM. A New Approach to the Measurement of Quality of Life: The Patient-Generated Index. Med Care. 1994 Nov;32(11):1109–26.

43. Kwasnicka D, Dombrowski SU, White M, Sniehotta F. Theoretical explanations for maintenance of behaviour change: a systematic review of behaviour theories. Health Psychol Rev. 2016 Jul 2;10(3):277–96.

44. Tang JA, Oh T, Scheer JK, Parsa AT. The current trend of administering a patient-generated index in the oncological setting: a systematic review. Oncol Rev [Internet]. 2014 Jun 30 [cited 2022 Oct 17]; Available from: https://www.frontierspartnerships.org/articles/10.4081/oncol.2014.245

45. Aaronson NK, Ahmedzai S, Bergman B, Bullinger M, Cull A, Duez NJ, et al. The European Organization for Research and Treatment of Cancer QLQ-C30: A Quality-of-Life Instrument for Use in International Clinical Trials in Oncology. JNCI J Natl Cancer Inst. 1993 Mar 3;85(5):365–76.

46. Herdman M, Gudex C, Lloyd A, Janssen Mf, Kind P, Parkin D, et al. Development and preliminary testing of the new five-level version of EQ-5D (EQ-5D-5L). Qual Life Res. 2011 Dec;20(10):1727–36.

47. ATS Statement: Guidelines for the Six-Minute Walk Test. Am J Respir Crit Care Med. 2002 Jul 1;166(1):111–7.

48. Schmidt K, Vogt L, Thiel C, Jäger E, Banzer W. Validity of the Six-Minute Walk Test in Cancer Patients. Int J Sports Med. 2013 Feb 26;34(07):631–6.

49. Spruit MA, Sillen MJH, Groenen MTJ, Wouters EFM, Franssen FME. New Normative Values for Handgrip Strength: Results From the UK Biobank. J Am Med Dir Assoc. 2013 Oct;14(10):775.e5-775.e11.

For peer review only - http://bmjopen.bmj.com/site/about/guidelines.xhtml

50. Musalek C, Kirchengast S. Grip Strength as an Indicator of Health-Related Quality of Life in Old Age—A Pilot Study. Int J Environ Res Public Health. 2017 Nov 24;14(12):1447.

51. Celis-Morales C, Welsh P, Lyall D, Steell L, Petermann F, Anderson J, et al. Associations of grip strength with cardiovascular, respiratory, and cancer outcomes and all cause mortality: prospective cohort study of half a million UK Biobank participants. 2018 Aug 5;361:1–10.

52. Bohannon RW. Grip Strength: An Indispensable Biomarker For Older Adults. Clin Interv Aging. 2019 Oct;Volume 14:1681–91.

53. Soysal P, Hurst C, Demurtas J, Firth J, Howden R, Yang L, et al. Handgrip strength and health outcomes: Umbrella review of systematic reviews with meta-analyses of observational studies. J Sport Health Sci. 2021 May;10(3):290–5.

54. Jiang R, Westwater ML, Noble S, Rosenblatt M, Dai W, Qi S, et al. Associations between grip strength, brain structure, and mental health in > 40,000 participants from the UK Biobank. BMC Med. 2022 Sep 9;20(1):286.

55. Saracino R, Kolva E, Rosenfeld B, Breitbart W. Measuring social support in patients with advanced medical illnesses: An analysis of the Duke–UNC Functional Social Support Questionnaire. Palliat Support Care. 2015 Oct;13(5):1153–63.

56. Kallogjeri D, Piccirillo JF, Spitznagel EL, Steyerberg EW. Comparison of scoring methods for ACE-27: Simpler is better. J Geriatr Oncol. 2012 Jul;3(3):238–45.

57. London Cancer Alliance. Guidance for Level Two Healthcare Workers and Level Three/Four Supervisors [Internet]. London Cancer Alliance; 2015. Available from: https://rmpartners.nhs.uk/wp-content/uploads/2017/03/lca-guidance-for-level-2-healthcare-workers-and-level-3_4-supervisors-september-2015.pdf

For peer review only

Figure one. Co-design approach used for the development of the personalised prehabilitation programme.

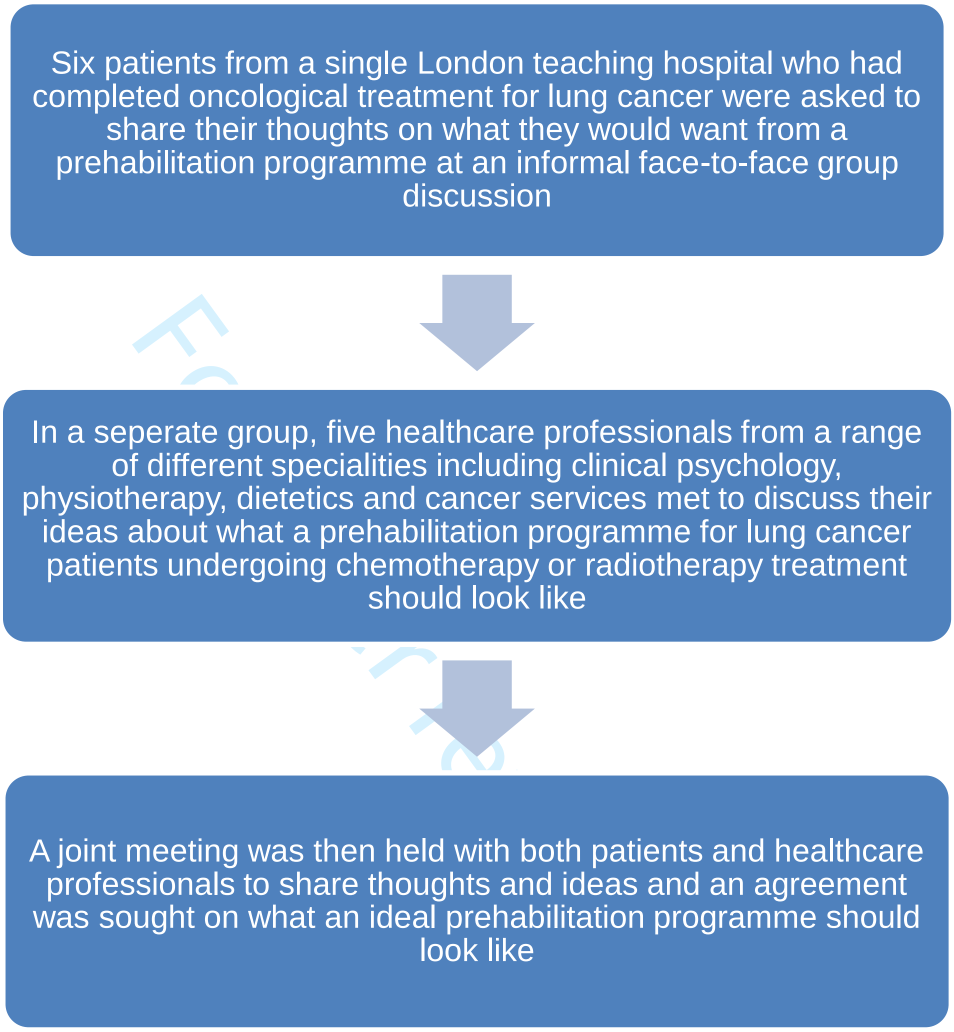

Figure two. Diagrammatic representation of the Adversity, Restoration and Compatibility (ARC) model of the experience of adjusting to living with and beyond cancer. The waves and curves reflect the fluidity and non-linear nature of process.

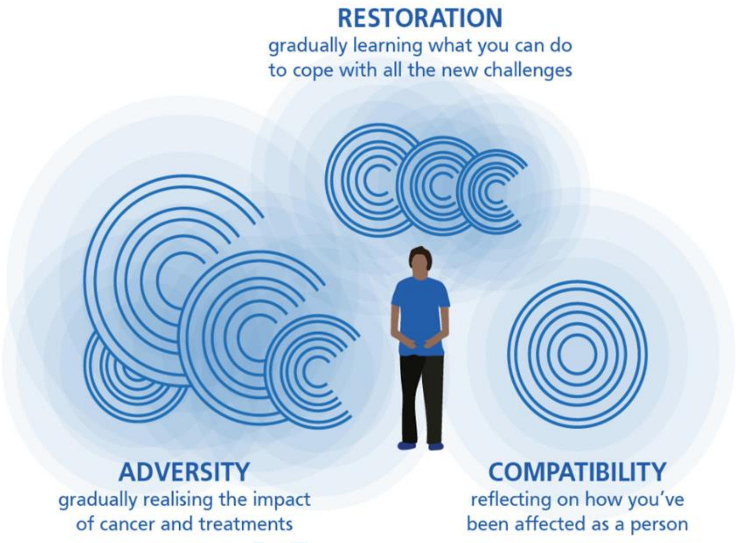

For peer review only

Figures 3a, 3b and 3c. Personalised prehabilitation

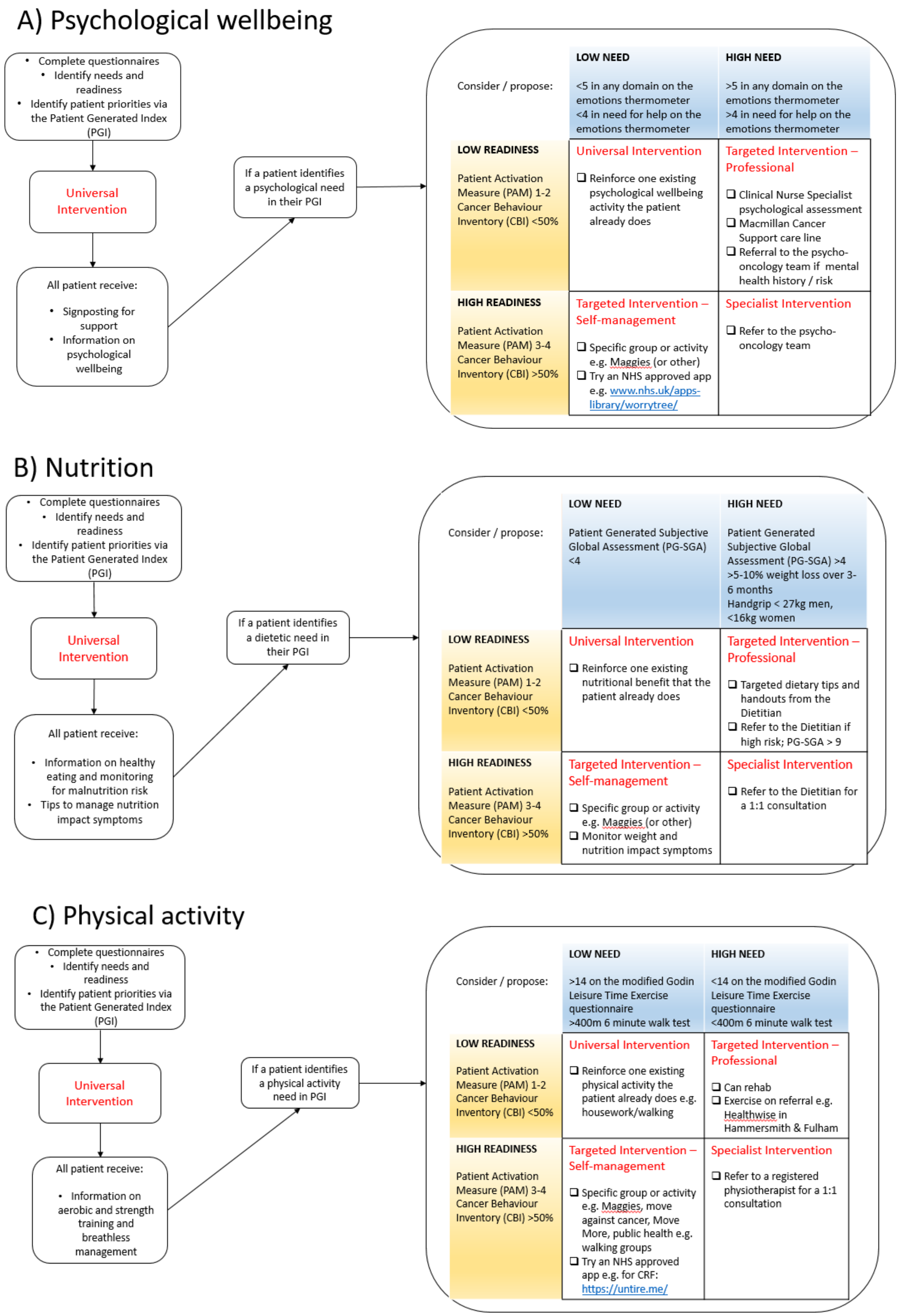

BMJ Open

Is personalised prehabilitation feasible to implement for patients undergoing oncological treatment for lung cancer at a London teaching hospital? Protocol of a feasibility study.

Journal:	*BMJ Open*
Manuscript ID	bmjopen-2023-072367.R1
Article Type:	Protocol
Date Submitted by the Author:	05-May-2023
Complete List of Authors:	Wade-Mcbane, Kelly; Imperial College Healthcare NHS Trust<br>King, Alex; Imperial College Healthcare NHS Trust<br>Urch, Catherine; Imperial College Healthcare NHS Trust<br>Johansson, Lina; Imperial College Healthcare NHS Trust<br>Wells, Mary; Imperial College Healthcare NHS Trust
**Primary Subject Heading**:	Oncology
Secondary Subject Heading:	Patient-centred medicine
Keywords:	CHEMOTHERAPY, RADIOTHERAPY, Respiratory tract tumours < ONCOLOGY

SCHOLARONE™ Manuscripts

Is personalised prehabilitation feasible to implement for patients undergoing oncological treatment for lung cancer at a London teaching hospital? Protocol of a feasibility study.

Kelly Wade-Mcbane[1], Alex King[1], Catherine Urch[1], Lina Johansson[1], Mary Wells[1]

[1]Imperial College Healthcare NHS Trust, London, UK

Correspondence: Kelly Wade-Mcbane, Nutrition and Dietetics Department, Charing Cross Hospital, Imperial College Healthcare NHS Trust, London, UK. Email: k.wade-mcbane@nhs.net

Abstract

Introduction

There is significant potential to improve outcomes for patients with lung cancer in terms of quality of life and survival. There is some evidence that prehabilitation can help, but, to date, this has only been tested in surgical populations, despite 70-80% of lung cancer patients in the UK receiving non-surgical treatment. The physiological and psychological benefits of prehabilitation seen in surgical patients could be extrapolated to those receiving non-surgical treatment, particularly in such a poor prognosis group.

With patients and healthcare professionals, we have co-designed a personalised and evidence-based prehabilitation programme. This draws on a conceptual framework that aligns with patient values and needs as well as functional goals. We aim to investigate whether this programme is feasible to implement and evaluate in clinical practice.

Methods and analysis

An open-label, single-group feasibility study incorporating quantitative assessments, a qualitative free text questionnaire and reflective field notes. Thirty participants will be recruited over an eight-month period from a single London teaching hospital. All recruited participants will receive a personalised prehabilitation programme during their oncological treatment. This includes a one-hour face-to-face appointment prior to, at week three and at week six of their treatment regimen as well as a weekly telephone call. Interventions including nutrition, physical activity and psychological wellbeing are stratified according to a patient's priorities, level of readiness and expressed needs. The primary outcome will be feasibility of the personalised prehabilitation programme in clinical practice by investigating areas of uncertainty regarding patient recruitment, attrition, treatment fidelity, intervention adherence and acceptability of study outcome measures. Secondary outcomes will include quality of life, functional capacity and grip strength.

Ethics and dissemination

Ethical approval has been obtained from the Health Research Authority (HRA) (reference number 22/PR/0390). Results of this study will be disseminated through publication in peer-reviewed articles, presentations at scientific conferences and in collaboration with patient and public involvement representatives.

Keywords: prehabilitation, physical activity, nutrition, wellbeing, chemotherapy, radiotherapy, immunotherapy, conceptual framework, psychological adjustment theory

For peer review only - http://bmjopen.bmj.com/site/about/guidelines.xhtml

Strengths and limitations of this study

- Feasibility study to assess whether a future definitive study can or should be done.
- Inclusive research approach, tailoring interventions to a participant's capabilities.
- Use of a stratified person-centred prehabilitation intervention co-designed with patient partners.
- Risk of potential bias owing to the lead researcher also being the clinician delivering the interventions.

Introduction

Multi-modal prehabilitation programmes incorporating nutrition, physical activity and psychological wellbeing are increasingly being used in surgical cancer pathways. This is in order to optimise health outcomes and enhance recovery by reducing post-operative complications (1–5) and hospital length of stay (4,6–8) as well as improving health reported quality of life (9–12). Few prehabilitation pathways exist for people who do not have surgery, despite the fact that 50-60% of people with cancer in the UK are treated with primary, neo-adjuvant or palliative chemotherapy and/or radiotherapy treatment (13). A small number of studies have investigated the use of prehabilitation in the neo-adjuvant setting and have shown benefit in terms of post-surgical outcomes, (14–18), but only one of these have been in lung cancer (19).

Only a minority of people with lung cancer are eligible for surgery, with 70-80% receiving non-surgical treatment within the UK (20) without any prehabilitation. UK deaths from lung cancer are higher than any other cancer and treatment outcomes are poor (21). There is significant potential to improve outcomes for people with lung cancer and there is some evidence that prehabilitation can help. However, this has only been tested in surgical populations. A small number of studies have shown that prehabilitation before lung cancer surgery is feasible and can improve functional capacity and reduce post-operative complications. However, most of these studies focused on the benefits of exercise prehabilitation as opposed to a multi-modal approach (22–30,19).

Prehabilitation during chemotherapy, radiotherapy or immunotherapy treatment for lung cancer poses significant challenges. Treatment regimes are often prolonged and people may experience a range of toxicities, which could limit their ability to engage in prehabilitation interventions. Personalisation to person-led values and needs is therefore important, as initiation and adherence to any intervention is determined by behavioural, psychological, physiological, environmental and social factors (31–33). This is further supported by Bayly et al's research in thoracic lung cancer (34,35) which found that participants had varied needs, highlighting the importance of tailored support to optimise symptom control, treatment tolerance and independence.

We have co-designed a multi-modal personalised prehabilitation programme with healthcare professionals and patients using a three-step approach. The multi-modal structure of the programme is informed by the Macmillan, Royal College of Anaesthetists and National Institute of Health Cancer and Nutrition Collaboration, Research Principles and Guidance for Prehabilitation within the Management and Support of People with Cancer (36). This guidance incorporates nutrition, physical activity and psychological wellbeing using a tiered

For peer review only - http://bmjopen.bmj.com/site/about/guidelines.xhtml

approach, tailored to whether patients need universal (applicable to anyone with cancer), targeted (applicable to those at risk of acute, chronic or latent effects of disease or treatment) or specialist (applicable to those with complex needs, severe impairment and/or disability) intervention (36). The first level of intervention – universal – comprises of written information and relevant signposting to support resources for all patients. If psychological, nutrition and/or physical activity needs are identified, patients then receive the appropriate intervention (targeted or specialist) according to their level of need and readiness.

The personalised prehabilitation programme uses the Adversity, Restoration and Compatibility (ARC) framework (32) to underpin a tailored and collaborative approach. The ARC framework provides a synthesised view of how people conceptualise the personal experience of living with and beyond cancer. Consistent with psychological adjustment theory (37), it frames this experience as an ongoing process of learning about the evolving challenge (Adversity), learning how to cope effectively (Restoration) and adapting one's identity (Compatibility), in parallel (see figure 1). Our programme provides a structure and process for personalising prehabilitation, which is person-centred rather than providing a purely logistical or psychometric approach. As recommend by Hickmann et al (38), such an approach could be key to empower patients to maintain progress through the prolonged, more variable context of peri-oncological prehabilitation.

This proposed feasibility study aims to investigate whether a personalised prehabilitation programme co-designed with healthcare professionals and patients and informed by theory and evidence is feasible to implement and evaluate in clinical practice and could thereby support a future definitive study.

Methods and analysis

Feasibility studies are particularly relevant to assess whether a future definitive study can or should be done (39–41). Using the Orsmond & Cohn framework for feasibility studies (42), this open-label, single-group study will use a mix of methods to understand the different aspects of feasibility. This will be through the inclusion of quantitative assessments, a qualitative free text questionnaire and reflective field notes to investigate areas of uncertainty regarding patient recruitment, attrition, treatment fidelity, intervention adherence and acceptability of study outcome measures. This is situated within the feasibility stage of the Medical Research Council (MRC) framework for developing and evaluating complex interventions (43). It will help inform if the personalised prehabilitation programme can be implemented and evaluated in clinical practice and will provide data to estimate the parameters required to design a future definitive study. The Consolidated Standards of Reporting Trials (CONSORT) reporting guideline for pilot and feasibility studies (39) has been used for this protocol. See supplemental material file 1.

Eligibility criteria

- Adults (≥18 years) with a diagnosis of lung cancer who are due to commence chemotherapy, radiotherapy or immunotherapy treatment. This study includes all lung cancer types and stages. People with co-morbidities and those on medication will be considered eligible. Interventions will be tailored to a participant's capabilities, thereby promoting inclusivity and optimisation of participant recruitment.

For peer review only - http://bmjopen.bmj.com/site/about/guidelines.xhtml

- Participants who are due to undergo lung cancer surgery, have had previous lung cancer surgery or unable to give informed consent will not be eligible for this study.

Recruitment

Participants will be recruited from the lung cancer clinic at a single London teaching hospital via referral from the Lung Cancer Multi-Disciplinary Team (LCMDT). Recruitment will continue until 30 participants have been recruited or until eight months have elapsed. A pragmatic sample size of 30 participants over an eight-month recruitment period from November 2022 – June 2023 has been selected, which is feasible within the time frame of this study. This is based on 144 - 240 new lung cancer diagnoses per year at the study hospital, with an assumption of a 50% uptake over an eight-month period. Furthermore, it is in accordance with published guidance on recommended sample sizes for feasibility studies (44,45).

The personalised prehabilitation programme

All participants will be required to attend three face-to-face appointments at the study hospital for study assessments, but the interventions will be delivered using a hybrid approach; either face-to-face at the study hospital or remotely via telephone or video conferencing depending on participant preference.

The three face-to-face sessions with the lead researcher will be prior to, at week three and at week six of a participant's oncological treatment regimen.
Following consent, participants will be guided through a set of questionnaires (as detailed below) to complete prior to their first face-to-face appointment. These questionnaires will be reviewed during the initial session, enabling the nutrition, physical activity and/or psychological wellbeing interventions to be stratified according to a participant's priorities, level of readiness and expressed needs:

- Cancer Behaviour Inventory (CBI) brief form - to measure self-efficacy for coping with cancer. A score of less than 50% indicates a low level of readiness, whereas a score of 50% or greater indicates a high level of readiness (46).
- Patient Activation Measure (PAM) - to measure a person's knowledge, skills and confidence in managing their own health and wellbeing. A score of one-two indicates a low level of readiness, whereas a score of three-four indicates a high level of readiness (47).
- Modified Godin leisure time exercise questionnaire - to measure activity. A score of 14 or greater demonstrates that a person is active, thereby indicating a low level of need for physical activity. Conversely, a score less than 14 demonstrates that a person is insufficiently active, thereby indicating a high level of need to optimise activity levels (48).
- Patient Generated Subjective Global Assessment (PG-SGA) short form - to identify malnutrition risk. A score less than four demonstrates no or minimal risk for malnutrition, thereby indicating a low level of need for nutritional support. Conversely, a score of four or greater demonstrates that a person is at risk of malnutrition or is malnourished, thereby indicating a high level of need for nutrition support (49).
- Emotions Thermometer (ET) - to evaluate levels of emotional distress. A score in any domain less than five or a score of less than four in need for help demonstrates low levels of emotional distress, thereby indicating a low level of need. Conversely, a score

For peer review only - http://bmjopen.bmj.com/site/about/guidelines.xhtml

of five or greater in any domain or a score of four or greater in need for help demonstrates high levels of emotional distress, thereby indicating a high level of need (50).

- Patient Generated Index (PGI) - to identify participant priorities (51).

Participant needs will also be determined objectively using the six-minute walk test (6MWT) to assess functional capacity and handgrip strength to assess upper body strength. Participants will undertake these functional assessments during the face-to-face sessions.

Questionnaire responses and outcomes from the functional measurements will inform the conversation with the participant about their priorities and enable interventions to be stratified according to a participant's level of readiness and need. If a subjective (questionnaire) or objective measurement (6MWT or handgrip strength) indicates a low or high level of readiness or need, the intervention will be tailored to the individual accordingly. See figures 2a, 2b and 2c.

The personalised prehabilitation programme uses a universal, targeted and specialist approach to prehabilitation interventions, in line with current guidance (36). All participants will receive a universal intervention comprising of written information and relevant signposting to cancer specific nutrition, physical activity and/or psychosocial wellbeing support resources. If a participant identifies a psychological, nutrition and/or physical activity need, they will then receive the appropriate intervention according to their level of need and readiness (see figures 2a, 2b and 2c). Targeted interventions will be delivered via the use of certified apps and/or specific group activities at Maggie's cancer centre (an independent charity that provides free cancer support, workshops and courses to help anyone affected by cancer). For example, exercise classes or nutritional educational sessions for participants undergoing treatment as well as the opportunity to attend a lung cancer support group. These group activities are run by cancer support workers or qualified instructors. Specialist interventions involve a 1:1 assessment with a registered healthcare professional such as a physiotherapist, dietitian and/or a clinical psychologist depending on a participant's needs. The objectives of specialist intervention with the physiotherapist and dietitian are to increase physical activity and optimise dietary intake to minimise loss of body weight and muscle mass, respectively. Specialist intervention with the physiotherapist will include tailored advice regarding resistance and aerobic exercise. In comparison, specialist intervention with the dietitian will include a full individualised nutritional assessment, taking into account anthropometric, biochemical and clinical parameters as well as undertaking a comprehensive diet history and calculation of nutritional requirements to minimise further malnutrition.

Owing to the prolonged nature of chemotherapy, radiotherapy or immunotherapy treatment in contrast to a defined one-off target such as surgery, person-led values and needs are likely to be more variable within the context of peri-oncological prehabilitation. Throughout treatment, participants will be asked to keep a daily diary to record their symptoms, appetite, mobility and level of distress each day using a Likert scale. This will provide participants with a visual representation of their progress and help identify any adverse trends.
Participants will also receive a weekly telephone call from the lead researcher (a specialist oncology dietitian). Participants will be able to use the diary to highlight any days they found particularly good or difficult and have the opportunity to discuss these. Personalisation is

For peer review only - http://bmjopen.bmj.com/site/about/guidelines.xhtml

central to this study and the language used during the face-to-face appointments and the weekly telephone calls has validation and compassion at its core to help build self-efficacy and sustain behaviour change (52). The use of the PGI to understand personal priorities is fundamental to this personalised approach. A systematic review (53) demonstrated the large potential value of using the PGI by enabling people with cancer to participate in the decision-making process. This is key to empower people to take ownership of their own health and for the sustainability of long-term positive behaviour change and self-care.

A traffic light system with varying levels of acceptability has often been cited as a means of judging feasibility (39,41,54–57). Following consensus amongst the research team, a priori feasibility endpoints were defined using a RAG criteria as shown in table 1.

Table 1. A priori feasibility endpoints using a RAG criteria.

Parameter	**Red** (don't proceed to a further study)	**Amber** (modifications +/- further study required)	**Green** (proceed to a larger study)
Recruitment rate	<30%	30-60%	>60%
Refusal rate	>50%	30-50%	<30%
Attrition rate	>80%	20-80%	<20%
Intervention adherence	<30%	30-60%	>60%

Primary objective and outcome measure

The primary objective is to assess the feasibility of the personalised prehabilitation programme in clinical practice by gathering data on how accessible and relevant it is for lung cancer patients undergoing chemotherapy, radiotherapy or immunotherapy treatment. This will be addressed using the research objectives and methods of assessment outlined in table 2 below.

Table 2. Research objectives and methods of assessment to investigate the feasibility of the personalised prehabilitation programme in clinical practice.

Research objective	Method of assessment(s)
To estimate eligibility, recruitment, refusal and attrition rates (including the reasons why)	Descriptive statistics – number approached, eligible, recruited, refused and number who drop out and at what stage<br>Field notes taken during the Lung Cancer Multi-Disciplinary Team (LCMDT)<br>Participant discussion
To assess adherence to the personalised prehabilitation interventions	Weekly telephone call – adherence to goals set
To assess the barriers and facilitators to adherence and intervention fidelity	Daily diary – symptom log<br>Weekly telephone call<br>Short free text questionnaire at the end of the study, analysed thematically
To evaluate acceptability of the study processes and outcome measures	Completion rates<br>Weekly telephone call<br>Short free text questionnaire at the end of the study, analysed thematically

For peer review only - http://bmjopen.bmj.com/site/about/guidelines.xhtml

To determine the 'prehabilitation window' (length of time between diagnosis and the start of treatment)	Descriptive statistics – date of diagnosis, date of start of treatment, date of start and finish of prehabilitation
To determine the extent to which the intervention/s are likely to have an impact on psychological wellbeing, nutrition and/or physical activity and quality of life	Questionnaires – change between baseline and follow up<br>Daily diary – symptom log<br>Weekly telephone call<br>Short free text questionnaire at the end of the study, analysed thematically]<br>Functional objective assessments
To determine the number of participants who require universal, targeted and specialist intervention	Questionnaires – level of need and readiness<br>Descriptive statistics

Heterogeneity of participant characteristics is expected within the study. This will provide valuable insights into how different people experience the personalised prehabilitation programme.

Secondary objectives and outcome measures

Functional benefits of prehabilitation can be seen in as little as two weeks (36,58). The secondary objectives will assess the impact of the personalised prehabilitation programme on:

- Overall quality of life as measured by:
  - European Organisation for Research and Treatment of Cancer (EORTC) QLQ-C30; a cancer specific assessment of quality of life, sensitive to changes over time (59)
  - EuroQOL group EQ-5D-5L for a descriptive overview of quality of life, which can be used for economic evaluation (60).
- Functional capacity as measured by the six-minute walk test (6MWT) (61). The 6MWT is a safe, valid and reliable measure of functional capacity in cancer populations (62). All participants will perform a standardised, self-paced 6MWT in a 30-metre long corridor. Each participant will be instructed to walk as far as they can for six minutes. Participants will be allowed to stop at any time. The distance covered (to the nearest metre) after the six minutes will be measured. A cut off value of 400m has been used to determine a participant's level of need for physical activity prehabilitation intervention. This is in line with a study by Kasymjanova et al (63) on the prognostic value of the 6MWT in advanced non-small cell lung cancer in patients undergoing chemotherapy.
- Handgrip strength as measured by a handgrip dynamometer (64). Grip strength has shown to be correlated with overall health status (65–69). Each participant will be instructed to sit in a chair and bend the elbow of their non-dominant arm at a 90-degree angle. The lead researcher will ask the participant to squeeze the device as hard as they can. Each participant will repeat this three times and the average will be recorded. A cut off value of 27kg for men and 16kg for women has been used to determine a participant's level of need for nutritional prehabilitation intervention. This is based on the revised European consensus guidelines on the definition and diagnosis of sarcopenia (70).

To tailor the intervention, the above outcome measures will be assessed prior to starting treatment and before the personalised prehabilitation programme begins (baseline), at week three and at week six of a participant's oncological treatment (end point).

For peer review only - http://bmjopen.bmj.com/site/about/guidelines.xhtml

Demographic and clinical data including age, gender, level of education achieved, ethnicity, employment status, level of social support (measured using the Duke-University of North Carolina Functional Social Support Questionnaire; DUFSSQ) (71), diagnosis, staging, treatment and co-morbidities (measured using the Adult Co-morbidity Evaluation (ACE) 27 score) (72) will be collected at baseline.

Training

The lead researcher is a highly specialised oncology dietitian trained in advanced communication and psychological skills (73) to manage complex conversations with patients and help recognise and respond to psychological distress. The lead researcher is familiar with using a handgrip dynamometer from previous experience in clinical practice. Training in conducting the 6MWT has been provided by a specialist oncology physiotherapist.

Patient and public involvement (PPI)

Four patients (and a spouse) who had recently completed chemotherapy or radiotherapy treatment for lung cancer were consulted on the personalised prehabilitation programme via a focus group. This was separate to focus groups used in the initial co-design process to develop the personalised prehabilitation programme, which used a three-step approach. See supplemental material file 2. Members of the focus group were very positive about the programme, with many patients reporting that they found having a strong mind-set, staying active and eating well helped them get through treatment. These are key components of the personalised prehabilitation programme. The focus group also reviewed the study questionnaires and patient documentation. Patients and their relatives expressed the benefits of having a weekly telephone call to discuss their concerns and reported that the questionnaires and daily diary would make them feel supported, as the information would be tailored to them individually.

All participants who participate in the study will be offered the opportunity to provide feedback on their experience of the personalised prehabilitation programme via the use of a short free text questionnaire. This feedback will help inform future programme adjustments and have the potential to improve patient experience.

Data collection and management

Data will be collected and stored according to the Data Protection Act (2018) and in line with General Data Protection Regulation (GDPR).

All data included in this study will be kept as part of the participants clinical care record on a secure password protected electronic patient record system at the study hospital.

A subset of this data will be extracted and entered into a case report form, which will be kept on a secure password protected database at the study hospital and only accessed by the research team. This data will be pseudonymised and all participants will be given a unique study number.

Statistical analysis plan

This study includes a small cohort of lung cancer participants from a single London teaching hospital. There are no groups to detect differences between, therefore, no formal tests of statistical significance will be undertaken.

Descriptive statistics will be used to analyse baseline demographic and clinical data.

Continuous measures will be reported as means, standard deviations and/or medians along

For peer review only - http://bmjopen.bmj.com/site/about/guidelines.xhtml

with confidence intervals, while categorical data will be reported as percentages. Missing data will be reported where applicable.
Inductive thematic analysis will be used to analyse qualitative data from the short free text questionnaire regarding patient experience.
Feasibility will be determined if the a priori thresholds for feasibility have been met.
The data collected will be used to inform the design of a larger randomised pilot study.

Strengths and limitations
This study uses a novel way of delivering a multi-modal prehabilitation programme through the use of a personalised, inclusive research approach that aligns with patient values, needs and functional goals, with interventions tailored to a participant's capabilities. The personalised prehabilitation programme provides a structure and process for personalising prehabilitation that is person-centred and underpinned by a conceptual framework, which is consistent with psychological adjustment theory. This could be key to empower patients to maintain activity through the longer, more variable context of peri-oncological prehabilitation. This study will help address key uncertainties, which need to be resolved before implementation, including engagement and adherence to prehabilitation interventions in lung cancer patients receiving chemotherapy, radiotherapy or immunotherapy treatment.
This study is limited by (a) lack of a comparison group (b) the small sample size used (c) self-reported adherence to the prehabilitation interventions and (d) the lead researcher also being the clinician delivering the interventions and performing assessments. In order to mitigate potential bias regarding the latter, where possible, aspects of the programme have been pre-defined.

Ethics and dissemination
Ethical approval was obtained from the Health Research Authority (HRA) on 13th April 2022 (reference number 22/PR/0390) prior to the start of the study. Any modifications will be approved by the HRA. This study has been registered with ClinicalTrials.gov (NCT: 22CX7570). Imperial College Healthcare NHS Trust is the sponsor of the study. Monitoring and auditing will be conducted in accordance with the sponsor's policies and procedures. Results of this study will be disseminated to academics, stakeholders, cancer alliances, relevant charities and to the public and people with cancer. This will be through publication in peer-reviewed articles, presentations at scientific conferences, social media and collaboration with patient and public involvement representatives.

Author contributions

KWM is the principal investigator. KWM devised the clinical question, applied for funding and research ethics and was involved in the conception and design of the study and protocol. AK contributed to the study design and methods. AK and CU were involved in the conception and design of the study. LJ and MW contributed to all versions of the protocol. AK, CU and MW provided their expertise in cancer care and contributed to the knowledge needed for prehabilitation. CU, LJ and MW provided supervision and mentorship. All authors read and approved the final protocol.

For peer review only - http://bmjopen.bmj.com/site/about/guidelines.xhtml

Funding

This work was supported by Health Education England and the National Institute for Health and Care Research as part of an ICA Pre-doctoral Clinical and Practitioner Academic Fellowship (Grant award number: NIHR302680).

Competing interests

The authors declare that they have no competing interests.

References

1. Berkel AEM, Bongers BC, van Kamp MJS, Kotte H, Weltevreden P, de Jongh FHC, et al. The effects of prehabilitation versus usual care to reduce postoperative complications in high-risk patients with colorectal cancer or dysplasia scheduled for elective colorectal resection: study protocol of a randomized controlled trial. BMC Gastroenterol. 2018 Dec;18(1):29.

2. van Rooijen S, Carli F, Dalton S, Thomas G, Bojesen R, Le Guen M, et al. Multimodal prehabilitation in colorectal cancer patients to improve functional capacity and reduce postoperative complications: the first international randomized controlled trial for multimodal prehabilitation. BMC Cancer. 2019 Dec;19(1):98.

3. Moore J, Merchant Z, Rowlinson K, McEwan K, Evison M, Faulkner G, et al. Implementing a system-wide cancer prehabilitation programme: The journey of Greater Manchester's 'Prehab4cancer.' Eur J Surg Oncol. 2021 Mar;47(3):524–32.

4. Perry R, Herbert G, Atkinson C, England C, Northstone K, Baos S, et al. Pre-admission interventions (prehabilitation) to improve outcome after major elective surgery: a systematic review and meta-analysis. BMJ Open. 2021 Sep;11(9):e050806.

5. Molenaar CJ, van Rooijen SJ, Fokkenrood HJ, Roumen RM, Janssen L, Slooter GD. Prehabilitation versus no prehabilitation to improve functional capacity, reduce postoperative complications and improve quality of life in colorectal cancer surgery. Cochrane Colorectal Group, editor. Cochrane Database Syst Rev [Internet]. 2022 May 19 [cited 2023 Jan 26];2022(5). Available from: http://doi.wiley.com/10.1002/14651858.CD013259.pub2

6. Gillis C, Buhler K, Bresee L, Carli F, Gramlich L, Culos-Reed N, et al. Effects of Nutritional Prehabilitation, With and Without Exercise, on Outcomes of Patients Who Undergo Colorectal Surgery: A Systematic Review and Meta-analysis. Gastroenterology. 2018 Aug;155(2):391-410.e4.

7. Lambert JE, Hayes LD, Keegan TJ, Subar DA, Gaffney CJ. The Impact of Prehabilitation on Patient Outcomes in Hepatobiliary, Colorectal, and Upper Gastrointestinal Cancer Surgery: A PRISMA-Accordant Meta-analysis. Ann Surg. 2021 Jul;274(1):70–7.

8. Waterland JL, McCourt O, Edbrooke L, Granger CL, Ismail H, Riedel B, et al. Efficacy of Prehabilitation Including Exercise on Postoperative Outcomes Following Abdominal Cancer Surgery: A Systematic Review and Meta-Analysis. Front Surg. 2021 Mar 19;8:628848.

For peer review only - http://bmjopen.bmj.com/site/about/guidelines.xhtml

9. Treanor C, Kyaw T, Donnelly M. An international review and meta-analysis of prehabilitation compared to usual care for cancer patients. J Cancer Surviv. 2018 Feb;12(1):64–73.

10. Fulop A, Lakatos L, Susztak N, Szijarto A, Banky B. The effect of trimodal prehabilitation on the physical and psychological health of patients undergoing colorectal surgery: a randomised clinical trial. Anaesthesia. 2021 Jan;76(1):82–90.

11. Wu F, Rotimi O, Laza-Cagigas R, Rampal T. The Feasibility and Effects of a Telehealth-Delivered Home-Based Prehabilitation Program for Cancer Patients during the Pandemic. Curr Oncol. 2021 Jun 17;28(3):2248–59.

12. Allen SK, Brown V, White D, King D, Hunt J, Wainwright J, et al. Multimodal Prehabilitation During Neoadjuvant Therapy Prior to Esophagogastric Cancer Resection: Effect on Cardiopulmonary Exercise Test Performance, Muscle Mass and Quality of Life—A Pilot Randomized Clinical Trial. Ann Surg Oncol. 2022 Mar;29(3):1839–50.

13. Cancer Research UK. Cancer Research UK. Cancer treatment statistics 2013-2016 [Internet]. 2013 2016 [cited 2022 Oct 3]. Available from: https://www.cancerresearchuk.org/health-professional/cancer-statistics/treatment

14. West MA, Loughney L, Lythgoe D, Barben CP, Sripadam R, Kemp GJ, et al. Effect of prehabilitation on objectively measured physical fitness after neoadjuvant treatment in preoperative rectal cancer patients: a blinded interventional pilot study. Br J Anaesth. 2015 Feb;114(2):244–51.

15. Christensen JF, Simonsen C, Banck-Petersen A, Thorsen-Streit S, Herrstedt A, Djurhuus SS, et al. Safety and feasibility of preoperative exercise training during neoadjuvant treatment before surgery for adenocarcinoma of the gastro-oesophageal junction: Preoperative exercise training during neoadjuvant treatment for gastro-oesophageal junction adenocarcinoma. BJS Open. 2019 Feb;3(1):74–84.

16. Dewberry LC, Wingrove LJ, Marsh MD, Glode AE, Schefter TE, Leong S, et al. Pilot Prehabilitation Program for Patients With Esophageal Cancer During Neoadjuvant Therapy and Surgery. J Surg Res. 2019 Mar;235:66–72.

17. Halliday LJ, Doganay E, Wynter-Blyth V, Osborn H, Buckley J, Moorthy K. Adherence to Pre-operative Exercise and the Response to Prehabilitation in Oesophageal Cancer Patients. J Gastrointest Surg. 2021 Apr;25(4):890–9.

18. Ligthart-Melis GC, Weijs PJM, te Boveldt ND, Buskermolen S, Earthman CP, Verheul HMW, et al. Dietician-delivered intensive nutritional support is associated with a decrease in severe postoperative complications after surgery in patients with esophageal cancer: INS limits postoperative complications. Dis Esophagus. 2013 Aug;26(6):587–93.

19. Schmid S, Minnella EM, Pilon Y, Rokah M, Rayes R, Najmeh S, et al. Neoadjuvant Prehabilitation Therapy for Locally Advanced Non–Small-Cell Lung Cancer: Optimizing Outcomes Throughout the Trajectory of Care. Clin Lung Cancer. 2022 Nov;23(7):593–9.

20. Cancer Research UK. Cancer Research UK. Lung cancer treatment statistics 2013-2014 [Internet]. 2013 2014 [cited 2022 Oct 17]. Available from: https://www.cancerresearchuk.org/health-professional/cancer-statistics/statistics-by-cancer-type/lung-cancer/diagnosis-and-treatment#heading-Three

For peer review only - http://bmjopen.bmj.com/site/about/guidelines.xhtml

21. Cancer Research UK. Cancer Research UK. Lung cancer mortality statistics 2017-2019 [Internet]. 2017 2019 [cited 2022 Oct 17]. Available from: https://www.cancerresearchuk.org/health-professional/cancer-statistics/statistics-by-cancer-type/lung-cancer#heading-Five

22. Laurent H, Aubreton S, Galvaing G, Pereira B, Merle P, Richard R, et al. Preoperative respiratory muscle endurance training improves ventilatory capacity and prevents pulmonary postoperative complications after lung surgery. Eur J Phys Rehabil Med [Internet]. 2020 Feb [cited 2022 Oct 17];56(1). Available from: https://www.minervamedica.it/index2.php?show=R33Y2020N01A0073

23. Liu Z, Qiu T, Pei L, Zhang Y, Xu L, Cui Y, et al. Two-Week Multimodal Prehabilitation Program Improves Perioperative Functional Capability in Patients Undergoing Thoracoscopic Lobectomy for Lung Cancer: A Randomized Controlled Trial. Anesth Analg. 2020 Sep;131(3):840–9.

24. Ricketts WM, Bollard K, Streets E, Hutton K, Hornby C, Lau K. Feasibility of setting up a pre-operative optimisation 'pre-hab' service for lung cancer surgery in the UK. Perioper Med. 2020 Dec;9(1):14.

25. Avancini A, Cavallo A, Trestini I, Tregnago D, Belluomini L, Crisafulli E, et al. Exercise prehabilitation in lung cancer: Getting stronger to recover faster. Eur J Surg Oncol. 2021 Aug;47(8):1847–55.

26. Bradley P, Merchant Z, Rowlinson-Groves K, Grundy S, Al-Najjar H, Brown L, et al. P212 Prehab4Cancer: an innovative regional lung cancer prehabilitation service. In: The lung cancer diagnostic journey [Internet]. BMJ Publishing Group Ltd and British Thoracic Society; 2021 [cited 2022 Oct 17]. p. A204.2-A205. Available from: https://thorax.bmj.com/lookup/doi/10.1136/thorax-2020-BTSabstracts.357

27. Ferreira V, Minnella EM, Awasthi R, Gamsa A, Ferri L, Mulder D, et al. Multimodal Prehabilitation for Lung Cancer Surgery: A Randomized Controlled Trial. Ann Thorac Surg. 2021 Nov;112(5):1600–8.

28. Goldsmith I, Chesterfield-Thomas G, Toghill H. Pre-treatment optimization with pulmonary rehabilitation in lung cancer: Making the inoperable patients operable. EClinicalMedicine. 2021 Jan;31:100663.

29. Bradley P, Merchant Z, Rowlinson-Groves K, Taylor M, Moore J, Evison M. Feasibility and outcomes of a real-world regional lung cancer prehabilitation programme in the UK. Br J Anaesth. 2022 Jul;S0007091222002951.

30. Granger C, Cavalheri V. Preoperative exercise training for people with non-small cell lung cancer. Cochrane Lung Cancer Group, editor. Cochrane Database Syst Rev [Internet]. 2022 Sep 28 [cited 2023 Jan 11];2022(9). Available from: http://doi.wiley.com/10.1002/14651858.CD012020.pub3

31. Ferreira V, Agnihotram RV, Bergdahl A, van Rooijen SJ, Awasthi R, Carli F, et al. Maximizing patient adherence to prehabilitation: what do the patients say? Support Care Cancer. 2018 Aug;26(8):2717–23.

32. Boutillier CL, Archer S, Barry C, King A, Mansfield L, Urch C. Conceptual framework for living with and beyond cancer: A systematic review and narrative synthesis. Psychooncology. 2019;28(5):948–59.

For peer review only - http://bmjopen.bmj.com/site/about/guidelines.xhtml

33. Grimmett C, Bradbury K, Dalton SO, Fecher-Jones I, Hoedjes M, Varkonyi-Sepp J, et al. The Role of Behavioral Science in Personalized Multimodal Prehabilitation in Cancer. Front Psychol. 2021;12:261.

34. Bayly J, Edwards BM, Peat N, Warwick G, Hennig IM, Arora A, et al. Developing an integrated rehabilitation model for thoracic cancer services: views of patients, informal carers and clinicians. Pilot Feasibility Stud. 2018 Dec;4(1):160.

35. Bayly J, Fettes L, Douglas E, Teixiera MJ, Peat N, Tunnard I, et al. Short-term integrated rehabilitation for people with newly diagnosed thoracic cancer: a multi-centre randomized controlled feasibility trial. Clin Rehabil. 2020 Feb;34(2):205–19.

36. Macmillan Cancer Support, NIHR Cancer and Nutrition Collaboration, Royal College of Anaesthetists. Principles and guidance for prehabilitation within the management and support of people with cancer [Internet]. Macmillan Cancer Support; 2019 [cited 2021 Oct 27]. Available from: https://www.macmillan.org.uk/healthcare-professionals/news-and-resources/guides/principles-and-guidance-for-prehabilitation

37. Brennan J. Adjustment to cancer?coping or personal transition? Psychooncology. 2001 Jan;10(1):1–18.

38. Hickmann E, Richter P, Schlieter H. All together now – patient engagement, patient empowerment, and associated terms in personal healthcare. BMC Health Serv Res. 2022 Sep 2;22(1):1116.

39. Eldridge SM, Chan CL, Campbell MJ, Bond CM, Hopewell S, Thabane L, et al. CONSORT 2010 statement: extension to randomised pilot and feasibility trials. BMJ. 2016 Oct 24;i5239.

40. Eldridge SM, Lancaster GA, Campbell MJ, Thabane L, Hopewell S, Coleman CL, et al. Defining Feasibility and Pilot Studies in Preparation for Randomised Controlled Trials: Development of a Conceptual Framework. Lazzeri C, editor. PLOS ONE. 2016 Mar 15;11(3):e0150205.

41. Pearson N, Naylor PJ, Ashe MC, Fernandez M, Yoong SL, Wolfenden L. Guidance for conducting feasibility and pilot studies for implementation trials. Pilot Feasibility Stud. 2020 Dec;6(1):167.

42. Orsmond GI, Cohn ES. The Distinctive Features of a Feasibility Study: Objectives and Guiding Questions. OTJR Occup Particip Health. 2015 Jul;35(3):169–77.

43. Skivington K, Matthews L, Simpson SA, Craig P, Baird J, Blazeby JM, et al. A new framework for developing and evaluating complex interventions: update of Medical Research Council guidance. BMJ. 2021 Sep 30;n2061.

44. Julious SA. Sample size of 12 per group rule of thumb for a pilot study. Pharm Stat. 2005 Oct;4(4):287–91.

45. Sim J, Lewis M. The size of a pilot study for a clinical trial should be calculated in relation to considerations of precision and efficiency. J Clin Epidemiol. 2012 Mar;65(3):301–8.

46. Heitzmann CA, Merluzzi TV, Jean-Pierre P, Roscoe JA, Kirsh KL, Passik SD. Assessing self-efficacy for coping with cancer: development and psychometric analysis of the brief version of the Cancer Behavior Inventory (CBI-B). Psychooncology. 2011 Mar;20(3):302–12.

For peer review only - http://bmjopen.bmj.com/site/about/guidelines.xhtml

47. Hibbard JH, Mahoney ER, Stockard J, Tusler M. Development and Testing of a Short Form of the Patient Activation Measure. Health Serv Res. 2005 Dec;40(6p1):1918–30.

48. Godin G. The Godin-Shephard Leisure-Time Physical Activity Questionnaire. Health Fit J Can. 2011 Jan 30;18-22 Pages.

49. Ottery F. Definition of standardized nutritional assessment and interventional pathways in oncology. Nutrition. 1996 Jan;12:S15–9.

50. Harju E, Michel G, Roser K. A systematic review on the use of the emotion thermometer in individuals diagnosed with cancer. Psychooncology. 2019 Sep;28(9):1803–18.

51. Ruta DA, Garratt AM, Leng M, Russell IT, MacDonald LM. A New Approach to the Measurement of Quality of Life: The Patient-Generated Index. Med Care. 1994 Nov;32(11):1109–26.

52. Kwasnicka D, Dombrowski SU, White M, Sniehotta F. Theoretical explanations for maintenance of behaviour change: a systematic review of behaviour theories. Health Psychol Rev. 2016 Jul 2;10(3):277–96.

53. Tang JA, Oh T, Scheer JK, Parsa AT. The current trend of administering a patient-generated index in the oncological setting: a systematic review. Oncol Rev [Internet]. 2014 Jun 30 [cited 2022 Oct 17]; Available from: https://www.frontierspartnerships.org/articles/10.4081/oncol.2014.245

54. Thabane L, Ma J, Chu R, Cheng J, Ismaila A, Rios LP, et al. A tutorial on pilot studies: the what, why and how. BMC Med Res Methodol. 2010 Jan 6;10(1):1.

55. Avery KNL, Williamson PR, Gamble C, O'Connell Francischetto E, Metcalfe C, Davidson P, et al. Informing efficient randomised controlled trials: exploration of challenges in developing progression criteria for internal pilot studies. BMJ Open. 2017 Feb;7(2):e013537.

56. Hallingberg B, Turley R, Segrott J, Wight D, Craig P, Moore L, et al. Exploratory studies to decide whether and how to proceed with full-scale evaluations of public health interventions: a systematic review of guidance. Pilot Feasibility Stud. 2018 Dec;4(1):104.

57. Mellor K, Albury C, Dutton SJ, Eldridge S, Hopewell S. Recommendations for progression criteria during external randomised pilot trial design, conduct, analysis and reporting. Pilot Feasibility Stud. 2023 Apr 15;9(1):59, s40814-023-01291–5.

58. Faithfull S, Turner L, Poole K, Joy M, Manders R, Weprin J, et al. Prehabilitation for adults diagnosed with cancer: A systematic review of long-term physical function, nutrition and patient-reported outcomes. Eur J Cancer Care (Engl) [Internet]. 2019 Jul [cited 2021 Oct 27];28(4). Available from: https://onlinelibrary.wiley.com/doi/10.1111/ecc.13023

59. Aaronson NK, Ahmedzai S, Bergman B, Bullinger M, Cull A, Duez NJ, et al. The European Organization for Research and Treatment of Cancer QLQ-C30: A Quality-of-Life Instrument for Use in International Clinical Trials in Oncology. JNCI J Natl Cancer Inst. 1993 Mar 3;85(5):365–76.

60. Herdman M, Gudex C, Lloyd A, Janssen Mf, Kind P, Parkin D, et al. Development and preliminary testing of the new five-level version of EQ-5D (EQ-5D-5L). Qual Life Res. 2011 Dec;20(10):1727–36.

For peer review only - http://bmjopen.bmj.com/site/about/guidelines.xhtml

61. ATS Statement: Guidelines for the Six-Minute Walk Test. Am J Respir Crit Care Med. 2002 Jul 1;166(1):111–7.

62. Schmidt K, Vogt L, Thiel C, Jäger E, Banzer W. Validity of the Six-Minute Walk Test in Cancer Patients. Int J Sports Med. 2013 Feb 26;34(07):631–6.

63. Kasymjanova G, Correa JA, Kreisman H, Dajczman E, Pepe C, Dobson S, et al. Prognostic Value of the Six-Minute Walk in Advanced Non-small Cell Lung Cancer. J Thorac Oncol. 2009 May;4(5):602–7.

64. Spruit MA, Sillen MJH, Groenen MTJ, Wouters EFM, Franssen FME. New Normative Values for Handgrip Strength: Results From the UK Biobank. J Am Med Dir Assoc. 2013 Oct;14(10):775.e5-775.e11.

65. Musalek C, Kirchengast S. Grip Strength as an Indicator of Health-Related Quality of Life in Old Age—A Pilot Study. Int J Environ Res Public Health. 2017 Nov 24;14(12):1447.

66. Celis-Morales C, Welsh P, Lyall D, Steell L, Petermann F, Anderson J, et al. Associations of grip strength with cardiovascular, respiratory, and cancer outcomes and all cause mortality: prospective cohort study of half a million UK Biobank participants. 2018 Aug 5;361:1–10.

67. Bohannon RW. Grip Strength: An Indispensable Biomarker For Older Adults. Clin Interv Aging. 2019 Oct;Volume 14:1681–91.

68. Soysal P, Hurst C, Demurtas J, Firth J, Howden R, Yang L, et al. Handgrip strength and health outcomes: Umbrella review of systematic reviews with meta-analyses of observational studies. J Sport Health Sci. 2021 May;10(3):290–5.

69. Jiang R, Westwater ML, Noble S, Rosenblatt M, Dai W, Qi S, et al. Associations between grip strength, brain structure, and mental health in > 40,000 participants from the UK Biobank. BMC Med. 2022 Sep 9;20(1):286.

70. Cruz-Jentoft AJ, Bahat G, Bauer J, Boirie Y, Bruyère O, Cederholm T, et al. Sarcopenia: revised European consensus on definition and diagnosis. Age Ageing. 2019 Jan 1;48(1):16–31.

71. Saracino R, Kolva E, Rosenfeld B, Breitbart W. Measuring social support in patients with advanced medical illnesses: An analysis of the Duke–UNC Functional Social Support Questionnaire. Palliat Support Care. 2015 Oct;13(5):1153–63.

72. Kallogjeri D, Piccirillo JF, Spitznagel EL, Steyerberg EW. Comparison of scoring methods for ACE-27: Simpler is better. J Geriatr Oncol. 2012 Jul;3(3):238–45.

73. London Cancer Alliance. Guidance for Level Two Healthcare Workers and Level Three/Four Supervisors [Internet]. London Cancer Alliance; 2015. Available from: https://rmpartners.nhs.uk/wp-content/uploads/2017/03/lca-guidance-for-level-2-healthcare-workers-and-level-3_4-supervisors-september-2015.pdf

For peer review only - http://bmjopen.bmj.com/site/about/guidelines.xhtml

Figure legends

Figure 1. Diagrammatic representation of the Adversity, Restoration and Compatibility (ARC) model of the experience of adjusting to living with and beyond cancer. The waves and curves reflect the fluidity and non-linear nature of process, with the sizes reducing as one adjusts to living with and beyond cancer.

Figures 2a, 2b and 2c. Examples of the models used in the personalised prehabilitation programme for psychological wellbeing, nutrition and physical activity.

For peer review only

For peer review only - http://bmjopen.bmj.com/site/about/guidelines.xhtml

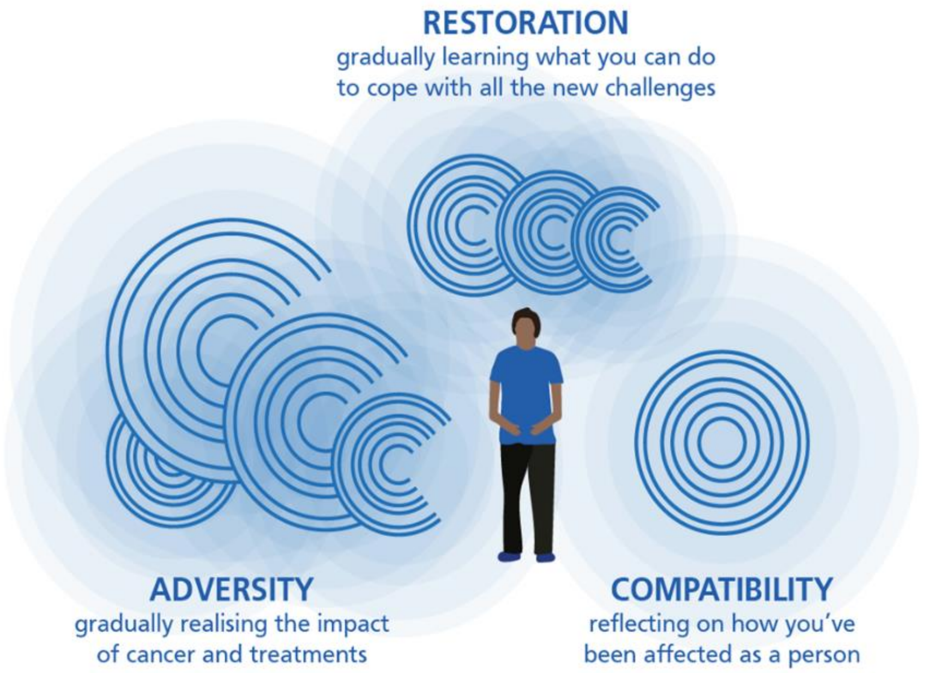

Figure 1. Diagrammatic representation of the Adversity, Restoration and Compatibility (ARC) model of the experience of adjusting to living with and beyond cancer. The waves and curves reflect the fluidity and non-linear nature of process, with the sizes reducing as one adjusts to living with and beyond cancer.

For peer review only

2a) Psychological wellbeing

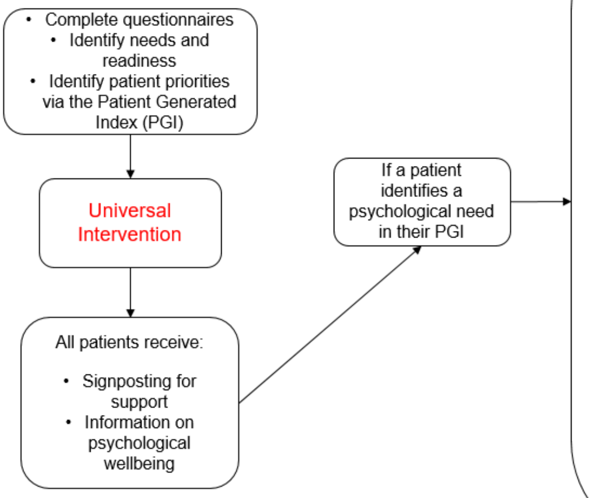

Consider / propose:	LOW NEED<br><5 in any domain on the emotions thermometer<br><4 in need for help on the emotions thermometer	HIGH NEED<br>>5 in any domain on the emotions thermometer<br>>4 in need for help on the emotions thermometer
**LOW READINESS**<br>Patient Activation Measure (PAM) 1-2<br>Cancer Behaviour Inventory (CBI) <50%	Universal Intervention<br>❑ Reinforce one existing psychological wellbeing activity the patient already does	Targeted Intervention – Professional<br>❑ Clinical Nurse Specialist psychological assessment<br>❑ Macmillan Cancer Support care line<br>❑ Referral to the psycho-oncology team if mental health history / risk
**HIGH READINESS**<br>Patient Activation Measure (PAM) 3-4<br>Cancer Behaviour Inventory (CBI) >50%	Targeted Intervention – Self-management<br>❑ Specific group or activity e.g. Maggies (or other)<br>❑ Try an NHS approved app e.g. www.nhs.uk/apps-library/worrytree/	Specialist Intervention<br>❑ Refer to the psycho-oncology team

2b) Nutrition

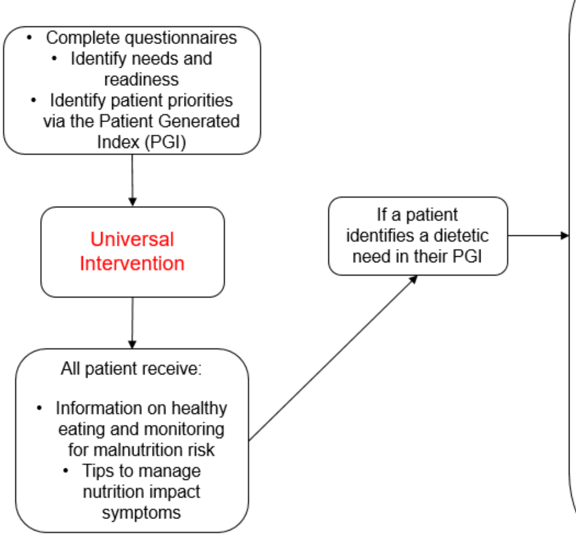

Consider / propose:	LOW NEED<br>Patient Generated Subjective Global Assessment (PG-SGA) <4	HIGH NEED<br>Patient Generated Subjective Global Assessment (PG-SGA) >4<br>>5-10% weight loss over 3-6 months<br>Handgrip < 27kg men, <16kg women
**LOW READINESS**<br>Patient Activation Measure (PAM) 1-2<br>Cancer Behaviour Inventory (CBI) <50%	Universal Intervention<br>❑ Reinforce one existing nutritional benefit that the patient already does	Targeted Intervention – Professional<br>❑ Targeted dietary tips and handouts from the Dietitian<br>❑ Refer to the Dietitian if high risk; PG-SGA > 9
**HIGH READINESS**<br>Patient Activation Measure (PAM) 3-4<br>Cancer Behaviour Inventory (CBI) >50%	Targeted Intervention – Self-management<br>❑ Specific group or activity e.g. Maggies (or other)<br>❑ Monitor weight and nutrition impact symptoms	Specialist Intervention<br>❑ Refer to the Dietitian for a 1:1 consultation

2c) Physical activity

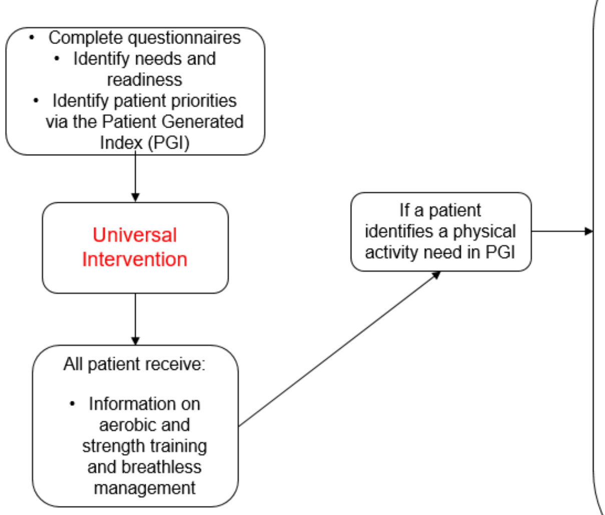

Consider / propose:	LOW NEED<br>>14 on the modified Godin Leisure Time Exercise questionnaire<br>>400m 6 minute walk test	HIGH NEED<br><14 on the modified Godin Leisure Time Exercise questionnaire<br><400m 6 minute walk test
**LOW READINESS**<br>Patient Activation Measure (PAM) 1-2<br>Cancer Behaviour Inventory (CBI) <50%	Universal Intervention<br>❑ Reinforce one existing physical activity the patient already does e.g. housework/walking	Targeted Intervention – Professional<br>❑ Can rehab<br>❑ Exercise on referral e.g. Healthwise in Hammersmith & Fulham
**HIGH READINESS**<br>Patient Activation Measure (PAM) 3-4<br>Cancer Behaviour Inventory (CBI) >50%	Targeted Intervention – Self-management<br>❑ Specific group or activity e.g. Maggies, move against cancer, Move More, public health e.g. walking groups<br>❑ Try an NHS approved app e.g. for CRF: https://untire.me/	Specialist Intervention<br>❑ Refer to a registered physiotherapist for a 1:1 consultation

Figures 2a, 2b and 2c. Examples of the models used in the personalised prehabilitation programme for psychological wellbeing, nutrition and physical activity.

Table 2 | CONSORT checklist of information to include when reporting a pilot trial

Section/topic and item No	Standard checklist item	Extension for pilot trials	Page No where item is reported
**Title and abstract**			
1a	Identification as a randomised trial in the title	Identification as a pilot or feasibility randomised trial in the title	Page 1
1b	Structured summary of trial design, methods, results, and conclusions (for specific guidance see CONSORT for abstracts)	Structured summary of pilot trial design, methods, results, and conclusions (for specific guidance see CONSORT abstract extension for pilot trials)	Page 1
**Introduction**			
Background and objectives:			
2a	Scientific background and explanation of rationale	Scientific background and explanation of rationale for future definitive trial, and reasons for randomised pilot trial	Pages 2-3
2b	Specific objectives or hypotheses	Specific objectives or research questions for pilot trial	Page 3
**Methods**			
Trial design:			
3a	Description of trial design (such as parallel, factorial) including allocation ratio	Description of pilot trial design (such as parallel, factorial) including allocation ratio	Page 3
3b	Important changes to methods after trial commencement (such as eligibility criteria), with reasons	Important changes to methods after pilot trial commencement (such as eligibility criteria), with reasons	N/A
Participants:			
4a	Eligibility criteria for participants		Page 3
4b	Settings and locations where the data were collected		Page 4
4c		How participants were identified and consented	Page 4
Interventions:			
5	The interventions for each group with sufficient details to allow replication, including how and when they were actually administered		Pages 4-5
Outcomes:			
6a	Completely defined prespecified primary and secondary outcome measures, including how and when they were assessed	Completely defined prespecified assessments or measurements to address each pilot trial objective specified in 2b, including how and when they were assessed	Pages 6-7
6b	Any changes to trial outcomes after the trial commenced, with reasons	Any changes to pilot trial assessments or measurements after the pilot trial commenced, with reasons	N/A
6c		If applicable, prespecified criteria used to judge whether, or how, to proceed with future definitive trial	Page 6
Sample size:			
7a	How sample size was determined	Rationale for numbers in the pilot trial	Page 4
7b	When applicable, explanation of any interim analyses and stopping guidelines		N/A
Randomisation:			
Sequence generation:			
8a	Method used to generate the random allocation sequence		N/A
8b	Type of randomisation; details of any restriction (such as blocking and block size)	Type of randomisation(s); details of any restriction (such as blocking and block size)	N/A
Allocation concealment mechanism:			
9	Mechanism used to implement the random allocation sequence (such as sequentially numbered containers), describing any steps taken to conceal the sequence until interventions were assigned		N/A
Implementation:			
10	Who generated the random allocation sequence, enrolled participants, and assigned participants to interventions		N/A
Blinding:			
11a	If done, who was blinded after assignment to interventions (eg, participants, care providers, those assessing outcomes) and how		N/A
11b	If relevant, description of the similarity of interventions		N/A
Analytical methods:			
12a	Statistical methods used to compare groups for primary and secondary outcomes	Methods used to address each pilot trial objective whether qualitative or quantitative	Pages 8-9
12b	Methods for additional analyses, such as subgroup analyses and adjusted analyses	Not applicable	N/A

Table 2 | CONSORT checklist of information to include when reporting a pilot trial

Section/topic and item No	Standard checklist item	Extension for pilot trials	Page No where item is reported
**Results**			
Participant flow (a diagram is strongly recommended):			
13a	For each group, the numbers of participants who were randomly assigned, received intended treatment, and were analysed for the primary outcome	For each group, the numbers of participants who were approached and/or assessed for eligibility, randomly assigned, received intended treatment, and were assessed for each objective	N/A – protocol paper
13b	For each group, losses and exclusions after randomisation, together with reasons		N/A – protocol paper
Recruitment:			
14a	Dates defining the periods of recruitment and follow-up		Page 4
14b	Why the trial ended or was stopped	Why the pilot trial ended or was stopped	N/A
Baseline data:			
15	A table showing baseline demographic and clinical characteristics for each group		N/A – protocol paper
Numbers analysed:			
16	For each group, number of participants (denominator) included in each analysis and whether the analysis was by original assigned groups	For each objective, number of participants (denominator) included in each analysis. If relevant, these numbers should be by randomised group	N/A – protocol paper
Outcomes and estimation:			
17a	For each primary and secondary outcome, results for each group, and the estimated effect size and its precision (such as 95% confidence interval)	For each objective, results including expressions of uncertainty (such as 95% confidence interval) for any estimates. If relevant, these results should be by randomised group	N/A – protocol paper
17b	For binary outcomes, presentation of both absolute and relative effect sizes is recommended	Not applicable	N/A
Ancillary analyses:			
18	Results of any other analyses performed, including subgroup analyses and adjusted analyses, distinguishing prespecified from exploratory	Results of any other analyses performed that could be used to inform the future definitive trial	N/A – protocol paper
Harms:			
19	All important harms or unintended effects in each group (for specific guidance see CONSORT for harms)		N/A – protocol paper
19a		If relevant, other important unintended consequences	N/A – protocol paper
**Discussion**			
Limitations:			
20	Trial limitations, addressing sources of potential bias, imprecision, and, if relevant, multiplicity of analyses	Pilot trial limitations, addressing sources of potential bias and remaining uncertainty about feasibility	Page 9
Generalisability:			
21	Generalisability (external validity, applicability) of the trial findings	Generalisability (applicability) of pilot trial methods and findings to future definitive trial and other studies	N/A – protocol paper
Interpretation:			
22	Interpretation consistent with results, balancing benefits and harms, and considering other relevant evidence	Interpretation consistent with pilot trial objectives and findings, balancing potential benefits and harms, and considering other relevant evidence	N/A – protocol paper
22a		Implications for progression from pilot to future definitive trial, including any proposed amendments	N/A – protocol paper
**Other information**			
Registration:			
23	Registration number and name of trial registry	Registration number for pilot trial and name of trial registry	Page 9
Protocol:			
24	Where the full trial protocol can be accessed, if available	Where the pilot trial protocol can be accessed, if available	N/A
Funding:			
25	Sources of funding and other support (such as supply of drugs), role of funders		Page 10
26		Ethical approval or approval by research review committee, confirmed with reference number	Page 9

Co-design approach used for the development of the personalised prehabilitation programme.

A participatory action research approach called experience-based co-design (EBCD) was undertaken in partnership with a single London teaching hospital and Maggie's cancer centre (an independent charity that provides free cancer support, workshops and courses to help anyone affected by cancer).

Participants who had completed oncological treatment for lung cancer were invited to an informal face-to-face group discussion. Participants were invited either in person at the monthly lung cancer support group held at Maggie's cancer centre or by their clinical oncologist or clinical nurse specialist at a follow up outpatient appointment.

Nine participants were invited to the informal group discussion and a total of six participants attended. The main reason for non-attendance was a lack of time. Participants were invited to share their thoughts on what they would want from a prehabilitation programme and through facilitated discussion, participant priorities were agreed. Many participants remarked on the importance of individualisation, being seeing face-to-face and the programme being endorsed by all members of the team.

Separately, five healthcare professionals from a range of different specialties including clinical psychology, physiotherapy, dietetics and cancer services met to discuss their ideas about what a prehabilitation programme for people with lung cancer undergoing chemotherapy or radiotherapy treatment should look like.

A joint meeting was then held with both participants and healthcare professionals to share thoughts and ideas and an agreement was sought on what an ideal prehabilitation programme should look like.

only

SPIRIT

STANDARD PROTOCOL ITEMS: RECOMMENDATIONS FOR INTERVENTIONAL TRIALS

SPIRIT 2013 Checklist: Recommended items to address in a clinical trial protocol and related documents*

Section/item	Item No	Description	Page / line number in main document
**Administrative information**			
Title	1	Descriptive title identifying the study design, population, interventions, and, if applicable, trial acronym	Page 1
Trial registration	2a	Trial identifier and registry name. If not yet registered, name of intended registry	Page 9
	2b	All items from the World Health Organization Trial Registration Data Set	N/A
Protocol version	3	Date and version identifier	Page 1
Funding	4	Sources and types of financial, material, and other support	Page 10
Roles and responsibilities	5a	Names, affiliations, and roles of protocol contributors	Pages 1 and 9
	5b	Name and contact information for the trial sponsor	N/A
	5c	Role of study sponsor and funders, if any, in study design; collection, management, analysis, and interpretation of data; writing of the report; and the decision to submit the report for publication, including whether they will have ultimate authority over any of these activities	N/A
	5d	Composition, roles, and responsibilities of the coordinating centre, steering committee, endpoint adjudication committee, data management team, and other individuals or groups overseeing the trial, if applicable (see Item 21a for data monitoring committee)	N/A

For peer review only

**Introduction**			
Background and rationale	6a	Description of research question and justification for undertaking the trial, including summary of relevant studies (published and unpublished) examining benefits and harms for each intervention	Pages 2-3
	6b	Explanation for choice of comparators	N/A
Objectives	7	Specific objectives or hypotheses	Page 3
Trial design	8	Description of trial design including type of trial (eg, parallel group, crossover, factorial, single group), allocation ratio, and framework (eg, superiority, equivalence, noninferiority, exploratory)	Page 3
**Methods: Participants, interventions, and outcomes**			
Study setting	9	Description of study settings (eg, community clinic, academic hospital) and list of countries where data will be collected. Reference to where list of study sites can be obtained	Page 4
Eligibility criteria	10	Inclusion and exclusion criteria for participants. If applicable, eligibility criteria for study centres and individuals who will perform the interventions (eg, surgeons, psychotherapists)	Page 3
Interventions	11a	Interventions for each group with sufficient detail to allow replication, including how and when they will be administered	Pages 4-5
	11b	Criteria for discontinuing or modifying allocated interventions for a given trial participant (eg, drug dose change in response to harms, participant request, or improving/worsening disease)	See page 3 of the participant information sheet at the end of this checklist
	11c	Strategies to improve adherence to intervention protocols, and any procedures for monitoring adherence (eg, drug tablet return, laboratory tests)	See page 7 of the participant information sheet at the end of this checklist
	11d	Relevant concomitant care and interventions that are permitted or prohibited during the trial	N/A

For peer review only - http://bmjopen.bmj.com/site/about/guidelines.xhtml

Outcomes	12	Primary, secondary, and other outcomes, including the specific measurement variable (eg, systolic blood pressure), analysis metric (eg, change from baseline, final value, time to event), method of aggregation (eg, median, proportion), and time point for each outcome. Explanation of the clinical relevance of chosen efficacy and harm outcomes is strongly recommended	Pages 6-7
Participant timeline	13	Time schedule of enrolment, interventions (including any run-ins and washouts), assessments, and visits for participants. A schematic diagram is highly recommended (see Figure)	See page 6 of the participant information sheet at the end of this checklist
Sample size	14	Estimated number of participants needed to achieve study objectives and how it was determined, including clinical and statistical assumptions supporting any sample size calculations	Page 3
Recruitment	15	Strategies for achieving adequate participant enrolment to reach target sample size	Page 3
**Methods: Assignment of interventions (for controlled trials)**			
Allocation:			
Sequence generation	16a	Method of generating the allocation sequence (eg, computer-generated random numbers), and list of any factors for stratification. To reduce predictability of a random sequence, details of any planned restriction (eg, blocking) should be provided in a separate document that is unavailable to those who enrol participants or assign interventions	N/A
Allocation concealmen t mechanism	16b	Mechanism of implementing the allocation sequence (eg, central telephone; sequentially numbered, opaque, sealed envelopes), describing any steps to conceal the sequence until interventions are assigned	N/A

Implementation	16c	Who will generate the allocation sequence, who will enrol participants, and who will assign participants to interventions	N/A
Blinding (masking)	17a	Who will be blinded after assignment to interventions (eg, trial participants, care providers, outcome assessors, data analysts), and how	N/A
	17b	If blinded, circumstances under which unblinding is permissible, and procedure for revealing a participant's allocated intervention during the trial	N/A
**Methods: Data collection, management, and analysis**			
Data collection methods	18a	Plans for assessment and collection of outcome, baseline, and other trial data, including any related processes to promote data quality (eg, duplicate measurements, training of assessors) and a description of study instruments (eg, questionnaires, laboratory tests) along with their reliability and validity, if known. Reference to where data collection forms can be found, if not in the protocol	Pages 4-8
	18b	Plans to promote participant retention and complete follow-up, including list of any outcome data to be collected for participants who discontinue or deviate from intervention protocols	See page 3 of the participant information sheet at the end of this checklist
Data management	19	Plans for data entry, coding, security, and storage, including any related processes to promote data quality (eg, double data entry; range checks for data values). Reference to where details of data management procedures can be found, if not in the protocol	Page 8 and page 3 of the participant information sheet at the end of this checklist
Statistical methods	20a	Statistical methods for analysing primary and secondary outcomes. Reference to where other details of the statistical analysis plan can be found, if not in the protocol	Page 8

	20b	Methods for any additional analyses (eg, subgroup and adjusted analyses)	N/A
	20c	Definition of analysis population relating to protocol non-adherence (eg, as randomised analysis), and any statistical methods to handle missing data (eg, multiple imputation)	Pages 8-9
**Methods: Monitoring**			
Data monitoring	21a	Composition of data monitoring committee (DMC); summary of its role and reporting structure; statement of whether it is independent from the sponsor and competing interests; and reference to where further details about its charter can be found, if not in the protocol. Alternatively, an explanation of why a DMC is not needed	A DMC is not needed as this is a small, short-term feasibility pilot study with low risk
	21b	Description of any interim analyses and stopping guidelines, including who will have access to these interim results and make the final decision to terminate the trial	N/A
Harms	22	Plans for collecting, assessing, reporting, and managing solicited and spontaneously reported adverse events and other unintended effects of trial interventions or trial conduct	See reporting of adverse events from study protocol at the end of this checklist
Auditing	23	Frequency and procedures for auditing trial conduct, if any, and whether the process will be independent from investigators and the sponsor	N/A
**Ethics and dissemination**			
Research ethics approval	24	Plans for seeking research ethics committee/institutional review board (REC/IRB) approval	Page 9
Protocol amendments	25	Plans for communicating important protocol modifications (eg, changes to eligibility criteria, outcomes, analyses) to relevant parties (eg, investigators, REC/IRBs, trial participants, trial registries, journals, regulators)	Page 9

For peer review only - http://bmjopen.bmj.com/site/about/guidelines.xhtml

Consent or assent	26a	Who will obtain informed consent or assent from potential trial participants or authorised surrogates, and how (see Item 32)	The lead researcher. An example of the participant consent form can be found at the end of this checklist
	26b	Additional consent provisions for collection and use of participant data and biological specimens in ancillary studies, if applicable	N/A
Confidentiality	27	How personal information about potential and enrolled participants will be collected, shared, and maintained in order to protect confidentiality before, during, and after the trial	See pages 3-4 of the participant information sheet at the end of this checklist
Declaration of interests	28	Financial and other competing interests for principal investigators for the overall trial and each study site	Page 10
Access to data	29	Statement of who will have access to the final trial dataset, and disclosure of contractual agreements that limit such access for investigators	See page 3 of the participant information sheet at the end of this checklist
Ancillary and post-trial care	30	Provisions, if any, for ancillary and post-trial care, and for compensation to those who suffer harm from trial participation	N/A
Dissemination policy	31a	Plans for investigators and sponsor to communicate trial results to participants, healthcare professionals, the public, and other relevant groups (eg, via publication, reporting in results databases, or other data sharing arrangements), including any publication restrictions	Page 9
	31b	Authorship eligibility guidelines and any intended use of professional writers	N/A
	31c	Plans, if any, for granting public access to the full protocol, participant-level dataset, and statistical code	N/A

Appendices

For peer review only

Informed consent materials	32	Model consent form and other related documentation given to participants and authorised surrogates	See participant information sheet and example of the participant consent form at the end of this checklist
Biological specimens	33	Plans for collection, laboratory evaluation, and storage of biological specimens for genetic or molecular analysis in the current trial and for future use in ancillary studies, if applicable	N/A

*It is strongly recommended that this checklist be read in conjunction with the SPIRIT 2013 Explanation & Elaboration for important clarification on the items. Amendments to the protocol should be tracked and dated. The SPIRIT checklist is copyrighted by the SPIRIT Group under the Creative Commons "Attribution-NonCommercial-NoDerivs 3.0 Unported" license.

Imperial College Healthcare
NHS Trust

Kelly Wade-Mcbane

Imperial-lung-prehab@nhs.net

PATIENT INFORMATION SHEET

Study title

A personalised plan of diet, exercise and emotional support for patients having chemotherapy, radiotherapy or immunotherapy treatment for lung cancer.

Reviewed by: The London Bridge Research Ethics Committee

Invitation

We would like to invite you to take part in our research study. Before you decide, it is important that you understand why the research is being done and what it will involve. Please take time to read and consider this information. If anything is not clear, please ask one of the research team.

Purpose of the study

The term 'prehabilitation' refers to a period of time before treatment and often includes a combination of diet, exercise and/or wellbeing plans to help patients through their cancer treatment. The benefits of prehabilitation have been shown in patients having surgery, but there is little research into its use for patients having chemotherapy, radiotherapy or immunotherapy treatment.

This study will see if a personalised plan of diet, exercise and emotional support can be used in practice for patients having chemotherapy, radiotherapy or immunotherapy treatment for lung cancer.

This study is part of the researcher's educational higher degree.

Why have I been invited?

You have been invited to take part as you have been diagnosed with lung cancer and are about to start chemotherapy, radiotherapy or immunotherapy treatment.

Do I have to take part?

No, you do not have to take part. Your choice to take part in the study is entirely voluntary. To help with the development of the service, a member of your clinical team may ask you why you have decided not to participate. This is entirely optional and you do not have to provide a reason. Your decision about the research will not affect the medical care you receive.

For peer review only

What will happen to me if I decide to take part?

Firstly, you will be asked to provide written consent either in person or online using your personal computer. Following consent, you will be asked to complete five questionnaires on wellbeing, physical activity and diet. These questionnaires can be completed either on paper or online. These questionnaires will take approximately 30 minutes to complete.

You will then be invited to a face to face appointment which will last approximately one hour at Charing Cross Hospital. This appointment will be with the lead researcher, who is a specialist dietitian at Imperial College Healthcare NHS Trust. At this appointment, we will:

- Review your questionnaires
- Ask you to complete some further questionnaires related to quality of life, diet and activity
- Do an assessment of your walking. This will involve you walking at your own pace as far as you can in 6 minutes
- Measure your grip strength

We will then agree your personalised plan, taking into account your level of need and readiness; identified from the questionnaires and the goals you want to achieve. If your personalised plan identifies an emotional, diet or activity need, you will receive an intervention that is suited to you.

After this appointment, we will ask you to keep a daily diary to rate your symptoms, appetite, mobility and mood throughout your treatment by providing a score each day using a scale from 0-10. You can use this diary when we phone you each week to let us know how you are getting on and to talk about any days you have found particularly good or bad.

We will invite you for two further face to face appointments at the week three and week six of your chemotherapy, radiotherapy or immunotherapy treatment. At these appointments, we will repeat the questionnaires, assess your walking and measure your handgrip strength. Personalised goals will be agreed at each stage.

Please see the diagram on page six for further information.

What are the possible benefits of taking part?

We do not know if there will be any individual benefits, but the information we get might help inform future research to help improve the lives of lung cancer patients.

Are there any possible disadvantages or risks from taking part?

There are no risks from taking part in this study.

You will be asked to complete a walking test, questionnaires and keep a daily diary, which could be a burden.
The walking test involves you walking at your own pace over a 6 minute period. You can stop when you need to. Physical activity has shown to be safe in lung cancer patients and the walking test is widely used in cancer research.

For peer review only

The questionnaires are quick to complete (about 30 minutes) and will be used to personalise your care.

The daily diary is simple to use. You will be asked to provide a score each day using a scale to rate your concerns. Please see page seven for an example of the daily diary.

All research appointments will be scheduled for the same days as your clinical appointments to prevent you having to make additional trips to the hospital. Your clinical visits may be extended by approximately one hour for the research.

Will my General Practitioner (GP) be informed of my participation?

This is optional and will not affect your participation in the study. We can let your GP know if you wish and you can tell us about this in the consent form. If you wish for your GP to be informed, we will send them a written letter accompanied with a study information sheet.

Will I be reimbursed for taking part?

Yes, we offer up to £10 towards the cost of your travel if you are required to come into hospital on a day outside your usual clinical visits. Please retain your receipts. We will provide you with a travel expenses form. Once completed, you can take this to the cashier's desk at Charing Cross Hospital to be reimbursed.

What will happen if I don't want to carry on with the study?

Participation in the study is entirely voluntary and you can withdraw at any point. Withdrawal will not affect any medical care that you are receiving. If you no longer want to carry on with the research study, please email us at Imperial-lung-prehab@nhs.net. We will retain and use the information collected up to that point, but we will not collect any further information.

What happens at the end of the study?

If the results are positive, we will plan to do more testing and offer this to more people. We will provide a written summary of the findings to all participants. We will share the results in scientific journals and conferences, so that others can learn from this as well. Patients will not be identifiable from any report or publication.

Will my taking part in the study be kept confidential and what will happen to my data?

To ensure the confidentiality of all personal data, all members of the research team will follow the Imperial College Healthcare NHS Trust confidentiality policy. Any identifiable data gathered will be stored securely in accordance with the Trust policy on either a password protected Trust computer or under lock and key.

You will be given a unique study number that will be used from the start of the study. Files such as paper consent forms and questionnaires will be stored in research offices located within the clinical areas and will be secured either by lock and key or key code and accessible by only departmental staff or the Trust's security team. All paper documentation kept in these will be locked.

For peer review only

Your data will not be shared with anyone outside of your hospital treating team or local research team. Any data containing any identifiable information will only be available to the chief investigator and appropriate members of the study team on a delegation log. Your medical record will not be removed from the clinical areas.

All data will be collected according to the Data Protection Act (2018) and in line with General Data Protection Regulations (GDPR).

If you lose capacity to consent during the study, you will be withdrawn from the study. Identifiable data already collected with consent would be retained and used in the study. No further data would be collected or any other research procedures carried out on or in relation to you.

Once we have finished the study, we will keep some of the data so that we can check the results. We will write our reports in a way that no-one can work out that you took part in the study. You can find out more about how we use your information by contacting the research team at Imperial-lung-prehab@nhs.net.

How will we use information about you?

We will need to use information from you and from your medical records for this research project.

This information will include name, contact number, date of birth, contact details and proposed treatment plan held by the site and/or sponsor for the research. People will use this information to do the research or to check your records to make sure that the research is being done properly.

People who do not need to know who you are will not be able to see your name or contact details. Your data will have a code number instead.

We will keep all information about you safe and secure.

Once we have finished the study, we will keep some of the data so we can check the results. We will write our reports in a way that no-one can work out that you took part in the study.

What are your choices about how your information is used?

You can stop being part of the study at any time, without giving a reason, but we will keep information about you that we already have.

We need to manage your records in specific ways for the research to be reliable. This means that we won't be able to let you see or change the data we hold about you.

If you agree to take part in this study, you will have the option to take part in future research using your data saved from this study.

How do I find out more about how my information is used?

You can find out more about how we use your information at www.hra.nhs.uk/information-about-patients/

For peer review only

Alternatively you can:

- Ask one of the research team
- Send an email to Imperial-lung-prehab@nhs.net.
- Call us on 0203 311 7135
- Contact the Imperial Academic Health Science Centre Data Protection Officer by calling 020 7594 1862

What if we find something unexpected?

If anything of concern regarding your health comes to light during the study, your clinical team and GP will be notified.

What if something goes wrong?

Imperial College Healthcare NHS Trust holds standard NHS Hospital indemnity and insurance cover with NHS resolution for NHS Trusts in England, which apply to this study. This does not affect your legal rights to seek compensation.

If you are harmed due to someone's negligence, then you may have grounds for a legal action. Regardless of this, if you wish to complain, or have any concerns about any aspect of the way you have been treated during the course of this study then you should immediately inform the Investigator.

The normal National Health Service complaints mechanisms are also available to you. If you are still not satisfied with the response, you may contact the Imperial Academic Health Science Centre Research Governance and Integrity Team.

What if there is a problem?

If you wish to make a complaint or if you have any concerns about any aspect of the way you have been approached or treated during the course of this study, please contact the research team at Imperial-lung-prehab@nhs.net.
You can also contact the Patient Advice and Liaison Service (PALS) on 020 331 27777.
If you are still not satisfied with the response, you may contact the Imperial Academic Health Science Centre Research Governance and Integrity Team on 020 7594 1862.

Who has reviewed the study?

All research in the NHS is looked at by an independent group of people, called a Research Ethics Committee, to protect participants' interests. This study has been reviewed and given favourable ethical opinion for conduct in the NHS by The London Bridge Research Ethics Committee.

Thank you for considering to take part in this study

For peer review only

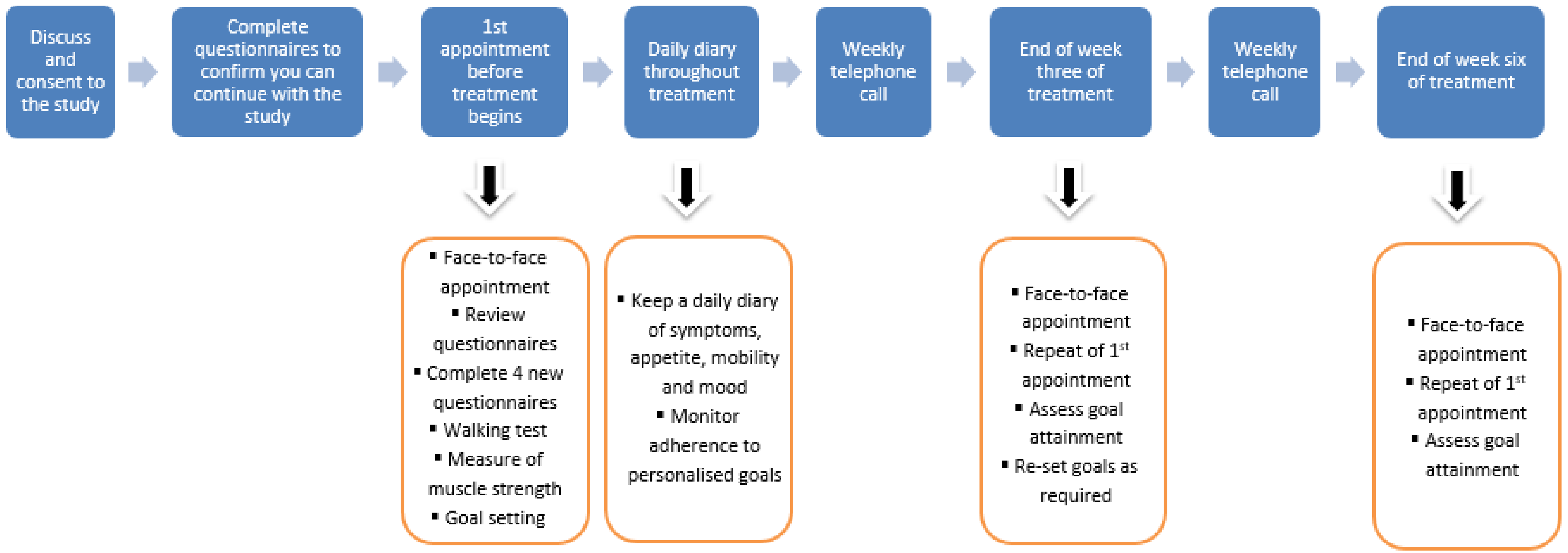
Discuss and consent to the study
Complete questionnaires to confirm you can continue with the study
1st appointment before treatment begins
Daily diary throughout treatment
Weekly telephone call
End of week three of treatment
Weekly telephone call
End of week six of treatment
▪ Face-to-face appointment
▪ Review questionnaires
▪ Complete 4 new questionnaires
▪ Walking test
▪ Measure of muscle strength
▪ Goal setting
▪ Keep a daily diary of symptoms, appetite, mobility and mood
▪ Monitor adherence to personalised goals
▪ Face-to-face appointment
▪ Repeat of 1st appointment
▪ Assess goal attainment
▪ Re-set goals as required
▪ Face-to-face appointment
▪ Repeat of 1st appointment
▪ Assess goal attainment

DIARY

Using this scale from 0-10 with 0 being no concerns and 10 being the worst it has been, please rate your symptoms, appetite, mobility and level of distress each day.

0 1 2 3 4 5 6 7 8 9 10

Cycle 1

	Monday	Tuesday	Wednesday	Thursday	Friday	Saturday	Sunday
Symptoms							
Appetite							
Mobility							
Distress							

At the end of the week, please tell us about any days you found particularly good or difficult. Highlight these and we will talk about them.

Imperial College Healthcare
NHS Trust

Kelly Wade-Mcbane

Imperial-lung-prehab@nhs.net

Participant identification number | | | | | |

CONSENT FORM

Study Title

A personalised prehabilitation model for patients undergoing chemotherapy, radiotherapy or immunotherapy treatment for lung cancer: A feasibility pilot study.

Name of Researcher: Kelly Wade-Mcbane

If you agree, please initial the box

1. I confirm that I have received a copy of the patient information sheet (version 7, 06/01/2023) for this study. I have read and considered the information and have had the opportunity to ask questions and have had these answered satisfactorily	
2. I understand that my participation is voluntary. I am free to withdraw at any time without having to explain and my medical care or legal rights will not be affected	
3. I understand that relevant sections of my medical data may be looked at by responsible individuals from Imperial College Healthcare NHS Trust or by regulatory authorities where it is relevant to my taking part in this research. I give permission for these individuals to have access to my records	
4. I agree / do not agree (delete as applicable) to my general practitioner (GP) being informed of my participation in the study. This is optional and will not affect your participation in the study	
5. I understand that in any report or publication on the results of this study, my identity will remain anonymous	
6. I give / do not give (delete as applicable) consent for information collected about me to be used to support other research in the future. This is optional and will not affect your participation in the study	
7. I agree to take part in this study	

_________________________ _______________ _________________________

Name of participant Date Signature

_________________________ _______________ _________________________

Name of person taking consent Date Signature

** 1 copy for participant; 1 copy for researcher's log; 1 copy to be uploaded into patient's* electronic medical notes

For peer review only - http://bmjopen.bmj.com/site/about/guidelines.xhtml

ADVERSE EVENTS

1.1. DEFINITIONS

Adverse Event (AE): any untoward medical occurrence in a patient or clinical study subject.

Serious Adverse Event (SAE): any untoward and unexpected medical occurrence or effect that:

- **Results in death**
- **Is life-threatening** – *refers to an event in which the subject was at risk of death at the time of the event; it does not refer to an event which hypothetically might have caused death if it were more severe*

- **Requires hospitalisation, or prolongation of existing inpatients' hospitalisation**
- **Results in persistent or significant disability or incapacity**
- **Is a congenital anomaly or birth defect**

Medical judgement should be exercised in deciding whether an AE is serious in other situations. Important AEs that are not immediately life-threatening or do not result in death or hospitalisation but may jeopardise the subject or may require intervention to prevent one of the other outcomes listed in the definition above, should also be considered serious.

1.2. REPORTING PROCEDURES

All adverse events should be reported. Depending on the nature of the event the reporting procedures below should be followed. Any questions concerning adverse event reporting should be directed to the Chief Investigator in the first instance.

5.3.1 Non serious AEs

All such events, whether expected or not, should be recorded- it should be specified if only some non-serious AEs will be recorded, any reporting should be consistent with the purpose of the trial end points.

5.3.2 Serious AEs

An SAE form should be completed and emailed to the Chief Investigator within 24 hours. However, relapse and death due to lung cancer, and hospitalisations for elective treatment of a pre-existing condition do not need reporting as SAEs.

All SAEs should be reported to the London Bridge Research Ethics Committee where in the opinion of the Chief Investigator, the event was:

- 'related', ie resulted from the administration of any of the research procedures; and
- 'unexpected', ie an event that is not listed in the protocol as an expected occurrence

Reports of related and unexpected SAEs should be submitted within 15 days of the Chief Investigator becoming aware of the event, using the NRES SAE form for non-IMP studies. The Chief Investigator must also notify the Sponsor of all related and unexpected SAEs.

Local investigators should report any SAEs as required by their Local Research Ethics Committee, Sponsor and/or Research & Development Office.

Contact details for reporting SAEs
RGIT@imperial.ac.uk

For peer review only - http://bmjopen.bmj.com/site/about/guidelines.xhtml

BMJ Open

Is personalised prehabilitation feasible to implement for patients undergoing oncological treatment for lung cancer at a London teaching hospital? Protocol of a feasibility trial.

Journal:	*BMJ Open*
Manuscript ID	bmjopen-2023-072367.R2
Article Type:	Protocol
Date Submitted by the Author:	28-Jun-2023
Complete List of Authors:	Wade-Mcbane, Kelly; Imperial College Healthcare NHS Trust<br>King, Alex; Imperial College Healthcare NHS Trust<br>Urch, Catherine; Imperial College Healthcare NHS Trust<br>Johansson, Lina; Imperial College Healthcare NHS Trust<br>Wells, Mary; Imperial College Healthcare NHS Trust
**Primary Subject Heading**:	Oncology
Secondary Subject Heading:	Patient-centred medicine
Keywords:	CHEMOTHERAPY, RADIOTHERAPY, Respiratory tract tumours < ONCOLOGY

SCHOLARONE™ Manuscripts

Is personalised prehabilitation feasible to implement for patients undergoing oncological treatment for lung cancer at a London teaching hospital? Protocol of a feasibility trial.

Kelly Wade-Mcbane[1], Alex King[1], Catherine Urch[1], Lina Johansson[1], Mary Wells[1]

[1]Imperial College Healthcare NHS Trust, London, UK

Correspondence: Kelly Wade-Mcbane, Nutrition and Dietetics Department, Charing Cross Hospital, Imperial College Healthcare NHS Trust, London, UK. Email: k.wade-mcbane@nhs.net

Abstract

Introduction
There is significant potential to improve outcomes for patients with lung cancer in terms of quality of life and survival. There is some evidence that prehabilitation can help, but, to date, this has only been tested in surgical populations, despite 70-80% of lung cancer patients in the UK receiving non-surgical treatment. The physiological and psychological benefits of prehabilitation seen in surgical patients could be extrapolated to those receiving non-surgical treatment, particularly in such a poor prognosis group.
With patients and healthcare professionals, we have co-designed a personalised and evidence-based prehabilitation programme. This draws on a conceptual framework that aligns with patient values and needs as well as functional goals. We aim to investigate whether this programme is feasible to implement and evaluate in clinical practice.

Methods and analysis
An open-label, single-group feasibility study incorporating quantitative assessments, a qualitative free text questionnaire and reflective field notes. Thirty participants will be recruited over an eight-month period from a single London teaching hospital. All recruited participants will receive a personalised prehabilitation programme during their oncological treatment. This includes a one-hour face-to-face appointment prior to, at week three and at week six of their treatment regimen as well as a weekly telephone call. Interventions including nutrition, physical activity and psychological wellbeing are stratified according to a patient's priorities, level of readiness and expressed needs. The primary outcome will be feasibility of the personalised prehabilitation programme in clinical practice by investigating areas of uncertainty regarding patient recruitment, attrition, treatment fidelity, intervention adherence and acceptability of study outcome measures. Secondary outcomes will include quality of life, functional capacity and grip strength.

Ethics and dissemination
Ethical approval has been obtained from the Health Research Authority (HRA) (reference number 22/PR/0390). Results of this study will be disseminated through publication in peer-reviewed articles, presentations at scientific conferences and in collaboration with patient and public involvement representatives.

Keywords: prehabilitation, physical activity, nutrition, wellbeing, chemotherapy, radiotherapy, immunotherapy, conceptual framework, psychological adjustment theory

For peer review only - http://bmjopen.bmj.com/site/about/guidelines.xhtml

Strengths and limitations of this study

- Feasibility study to assess whether a future definitive study can or should be done.
- Inclusive research approach, tailoring interventions to a participant's capabilities.
- Use of a stratified person-centred prehabilitation intervention co-designed with patient partners.
- Risk of potential bias owing to the lead researcher also being the clinician delivering the interventions.

Introduction

Multi-modal prehabilitation programmes incorporating nutrition, physical activity and psychological wellbeing are increasingly being used in surgical cancer pathways. This is in order to optimise health outcomes and enhance recovery by reducing post-operative complications (1–5) and hospital length of stay (4,6–8) as well as improving health reported quality of life (9–12). Few prehabilitation pathways exist for people who do not have surgery, despite the fact that 50-60% of people with cancer in the UK are treated with primary, neo-adjuvant or palliative chemotherapy and/or radiotherapy treatment (13). A small number of studies have investigated the use of prehabilitation in the neo-adjuvant setting and have shown benefit in terms of post-surgical outcomes, (14–18), but only one of these have been in lung cancer (19).

Only a minority of people with lung cancer are eligible for surgery, with 70-80% receiving non-surgical treatment within the UK (20) without any prehabilitation. UK deaths from lung cancer are higher than any other cancer and treatment outcomes are poor (21). There is significant potential to improve outcomes for people with lung cancer and there is some evidence that prehabilitation can help. However, this has only been tested in surgical populations. A small number of studies have shown that prehabilitation before lung cancer surgery is feasible and can improve functional capacity and reduce post-operative complications. However, most of these studies focused on the benefits of exercise prehabilitation as opposed to a multi-modal approach (22–30,19).

Prehabilitation during chemotherapy, radiotherapy or immunotherapy treatment for lung cancer poses significant challenges. Treatment regimes are often prolonged and people may experience a range of toxicities, which could limit their ability to engage in prehabilitation interventions. Personalisation to person-led values and needs is therefore important, as initiation and adherence to any intervention is determined by behavioural, psychological, physiological, environmental and social factors (31–33). This is further supported by Bayly et al's research in thoracic lung cancer (34,35) which found that participants had varied needs, highlighting the importance of tailored support to optimise symptom control, treatment tolerance and independence.

We have co-designed a multi-modal personalised prehabilitation programme with healthcare professionals and patients using a three-step approach. See supplemental material file 1. The multi-modal structure of the programme is informed by the Macmillan, Royal College of Anaesthetists and National Institute of Health Cancer and Nutrition Collaboration, Research Principles and Guidance for Prehabilitation within the Management and Support of People with Cancer (36). This guidance incorporates nutrition, physical activity and psychological

For peer review only - http://bmjopen.bmj.com/site/about/guidelines.xhtml

wellbeing using a tiered approach, tailored to whether patients need universal (applicable to anyone with cancer), targeted (applicable to those at risk of acute, chronic or latent effects of disease or treatment) or specialist (applicable to those with complex needs, severe impairment and/or disability) intervention (36). The first level of intervention – universal – comprises of written information and relevant signposting to support resources for all patients. If psychological, nutrition and/or physical activity needs are identified, patients then receive the appropriate intervention (targeted or specialist) according to their level of need and readiness.

The personalised prehabilitation programme uses the Adversity, Restoration and Compatibility (ARC) framework (32) to underpin a tailored and collaborative approach. The ARC framework provides a synthesised view of how people conceptualise the personal experience of living with and beyond cancer. Consistent with psychological adjustment theory (37), it frames this experience as an ongoing process of learning about the evolving challenge (Adversity), learning how to cope effectively (Restoration) and adapting one's identity (Compatibility), in parallel (see figure 1). Our programme provides a structure and process for personalising prehabilitation, which is person-centred rather than providing a purely logistical or psychometric approach. As recommend by Hickmann et al (38), such an approach could be key to empower patients to maintain progress through the prolonged, more variable context of peri-oncological prehabilitation.

This proposed feasibility study aims to investigate whether a personalised prehabilitation programme co-designed with healthcare professionals and patients and informed by theory and evidence is feasible to implement and evaluate in clinical practice and could thereby support a future definitive study.

Methods and analysis

Feasibility studies are particularly relevant to assess whether a future definitive study can or should be done (39–41). Using the Orsmond & Cohn framework for feasibility studies (42), this open-label, single-group study will use a mix of methods to understand the different aspects of feasibility. This will be through the inclusion of quantitative assessments, a qualitative free text questionnaire and reflective field notes to investigate areas of uncertainty regarding patient recruitment, attrition, treatment fidelity, intervention adherence and acceptability of study outcome measures. This is situated within the feasibility stage of the Medical Research Council (MRC) framework for developing and evaluating complex interventions (43). It will help inform if the personalised prehabilitation programme can be implemented and evaluated in clinical practice and will provide data to estimate the parameters required to design a future definitive study. The Consolidated Standards of Reporting Trials (CONSORT) reporting guideline for pilot and feasibility studies (39) has been used for this protocol. See supplemental material file 2.

Eligibility criteria

Inclusion criteria: Adults (≥18 years) with a diagnosis of lung cancer who are due to commence chemotherapy, radiotherapy or immunotherapy treatment. This study includes all lung cancer types and stages. People with co-morbidities and those on medication will be considered eligible. Interventions will be tailored to a participant's capabilities, thereby promoting inclusivity and optimisation of participant recruitment.

For peer review only - http://bmjopen.bmj.com/site/about/guidelines.xhtml

Exclusion criteria: Participants who are due to undergo lung cancer surgery, have had previous lung cancer surgery or unable to give informed consent will not be eligible for this study.

Recruitment

Participants will be recruited from the lung cancer clinic at a single London teaching hospital via referral from the Lung Cancer Multi-Disciplinary Team (LCMDT). Recruitment will continue until 30 participants have been recruited or until eight months have elapsed. A pragmatic sample size of 30 participants over an eight-month recruitment period from November 2022 – June 2023 has been selected, which is feasible within the time frame of this study. This is based on 144 - 240 new lung cancer diagnoses per year at the study hospital, with an assumption of a 50% uptake over an eight-month period. Furthermore, it is in accordance with published guidance on recommended sample sizes for feasibility studies (44,45).

The personalised prehabilitation programme

All participants will be required to attend three face-to-face appointments at the study hospital for study assessments, but the interventions will be delivered using a hybrid approach; either face-to-face at the study hospital or remotely via telephone or video conferencing depending on participant preference.

The three face-to-face sessions with the lead researcher will be prior to, at week three and at week six of a participant's oncological treatment regimen.
Following consent, participants will be guided through a set of questionnaires (as detailed below) to complete prior to their first face-to-face appointment. These questionnaires will be reviewed during the initial session, enabling the nutrition, physical activity and/or psychological wellbeing interventions to be stratified according to a participant's priorities, level of readiness and expressed needs:

- Cancer Behaviour Inventory (CBI) brief form - to measure self-efficacy for coping with cancer. A score of less than 50% indicates a low level of readiness, whereas a score of 50% or greater indicates a high level of readiness (46).
- Patient Activation Measure (PAM) - to measure a person's knowledge, skills and confidence in managing their own health and wellbeing. A score of one-two indicates a low level of readiness, whereas a score of three-four indicates a high level of readiness (47).
- Modified Godin leisure time exercise questionnaire - to measure activity. A score of 14 or greater demonstrates that a person is active, thereby indicating a low level of need for physical activity. Conversely, a score less than 14 demonstrates that a person is insufficiently active, thereby indicating a high level of need to optimise activity levels (48).
- Patient Generated Subjective Global Assessment (PG-SGA) short form - to identify malnutrition risk. A score less than four demonstrates no or minimal risk for malnutrition, thereby indicating a low level of need for nutritional support. Conversely, a score of four or greater demonstrates that a person is at risk of malnutrition or is malnourished, thereby indicating a high level of need for nutrition support (49).
- Emotions Thermometer (ET) - to evaluate levels of emotional distress. A score in any domain less than five or a score of less than four in need for help demonstrates low

For peer review only - http://bmjopen.bmj.com/site/about/guidelines.xhtml

levels of emotional distress, thereby indicating a low level of need. Conversely, a score of five or greater in any domain or a score of four or greater in need for help demonstrates high levels of emotional distress, thereby indicating a high level of need (50).

- Patient Generated Index (PGI) - to identify participant priorities (51).

Participant needs will also be determined objectively using the six-minute walk test (6MWT) to assess functional capacity and handgrip strength to assess upper body strength. Participants will undertake these functional assessments during the face-to-face sessions.

Questionnaire responses and outcomes from the functional measurements will inform the conversation with the participant about their priorities and enable interventions to be stratified according to a participant's level of readiness and need. If a subjective (questionnaire) or objective measurement (6MWT or handgrip strength) indicates a low or high level of readiness or need, the intervention will be tailored to the individual accordingly. See figures 2a, 2b and 2c.

The personalised prehabilitation programme uses a universal, targeted and specialist approach to prehabilitation interventions, in line with current guidance (36). All participants will receive a universal intervention comprising of written information and relevant signposting to cancer specific nutrition, physical activity and/or psychosocial wellbeing support resources. If a participant identifies a psychological, nutrition and/or physical activity need, they will then receive the appropriate intervention according to their level of need and readiness (see figures 2a, 2b and 2c). Targeted interventions will be delivered via the use of certified apps and/or specific group activities at Maggie's cancer centre (an independent charity that provides free cancer support, workshops and courses to help anyone affected by cancer). For example, exercise classes or nutritional educational sessions for participants undergoing treatment as well as the opportunity to attend a lung cancer support group. These group activities are run by cancer support workers or qualified instructors. Specialist interventions involve a 1:1 assessment with a registered healthcare professional such as a physiotherapist, dietitian and/or a clinical psychologist depending on a participant's needs. The objectives of specialist intervention with the physiotherapist and dietitian are to increase physical activity and optimise dietary intake to minimise loss of body weight and muscle mass, respectively. Specialist intervention with the physiotherapist will include tailored advice regarding resistance and aerobic exercise. In comparison, specialist intervention with the dietitian will include a full individualised nutritional assessment, taking into account anthropometric, biochemical and clinical parameters as well as undertaking a comprehensive diet history and calculation of nutritional requirements to minimise further malnutrition.

Owing to the prolonged nature of chemotherapy, radiotherapy or immunotherapy treatment in contrast to a defined one-off target such as surgery, person-led values and needs are likely to be more variable within the context of peri-oncological prehabilitation. Throughout treatment, participants will be asked to keep a daily diary to record their symptoms, appetite, mobility and level of distress each day using a Likert scale. This will provide participants with a visual representation of their progress and help identify any adverse trends.
Participants will also receive a weekly telephone call from the lead researcher (a specialist oncology dietitian). Participants will be able to use the diary to highlight any days they found

For peer review only - http://bmjopen.bmj.com/site/about/guidelines.xhtml

particularly good or difficult and have the opportunity to discuss these. Personalisation is central to this study and the language used during the face-to-face appointments and the weekly telephone calls has validation and compassion at its core to help build self-efficacy and sustain behaviour change (52). The use of the PGI to understand personal priorities is fundamental to this personalised approach. A systematic review (53) demonstrated the large potential value of using the PGI by enabling people with cancer to participate in the decision-making process. This is key to empower people to take ownership of their own health and for the sustainability of long-term positive behaviour change and self-care.

A traffic light system with varying levels of acceptability has often been cited as a means of judging feasibility (39,41,54–57). Following consensus amongst the research team, a priori feasibility endpoints were defined using a RAG criteria as shown in table 1.

Table 1. A priori feasibility endpoints using a RAG criteria.

Parameter	Red (don't proceed to a further study)	Amber (modifications +/- further study required)	Green (proceed to a larger study)
Recruitment rate	<30%	30-60%	>60%
Refusal rate	>50%	30-50%	<30%
Attrition rate	>80%	20-80%	<20%
Intervention adherence	<30%	30-60%	>60%

Primary objective and outcome measure

The primary objective is to assess the feasibility of the personalised prehabilitation programme in clinical practice by gathering data on how accessible and relevant it is for lung cancer patients undergoing chemotherapy, radiotherapy or immunotherapy treatment. This will be addressed using the research objectives and methods of assessment outlined in table 2 below.

Table 2. Research objectives and methods of assessment to investigate the feasibility of the personalised prehabilitation programme in clinical practice.

Research objective	Method of assessment(s)
To estimate eligibility, recruitment, refusal and attrition rates (including the reasons why)	Descriptive statistics – number approached, eligible, recruited, refused and number who drop out and at what stage<br>Field notes taken during the Lung Cancer Multi-Disciplinary Team (LCMDT)<br>Participant discussion
To assess adherence to the personalised prehabilitation interventions	Weekly telephone call – adherence to goals set
To assess the barriers and facilitators to adherence and intervention fidelity	Daily diary – symptom log<br>Weekly telephone call<br>Short free text questionnaire at the end of the study, analysed thematically
To evaluate acceptability of the study processes and outcome measures	Completion rates<br>Weekly telephone call<br>Short free text questionnaire at the end of the study, analysed thematically

For peer review only - http://bmjopen.bmj.com/site/about/guidelines.xhtml

To determine the 'prehabilitation window' (length of time between diagnosis and the start of treatment)	Descriptive statistics – date of diagnosis, date of start of treatment, date of start and finish of prehabilitation
To determine the extent to which the intervention/s are likely to have an impact on psychological wellbeing, nutrition and/or physical activity and quality of life	Questionnaires – change between baseline and follow up<br>Daily diary – symptom log<br>Weekly telephone call<br>Short free text questionnaire at the end of the study, analysed thematically]<br>Functional objective assessments
To determine the number of participants who require universal, targeted and specialist intervention	Questionnaires – level of need and readiness<br>Descriptive statistics

Heterogeneity of participant characteristics is expected within the study. This will provide valuable insights into how different people experience the personalised prehabilitation programme.

Secondary objectives and outcome measures

Functional benefits of prehabilitation can be seen in as little as two weeks (36,58). The secondary objectives will assess the impact of the personalised prehabilitation programme on:

- Overall quality of life as measured by:
  - European Organisation for Research and Treatment of Cancer (EORTC) QLQ-C30; a cancer specific assessment of quality of life, sensitive to changes over time (59)
  - EuroQOL group EQ-5D-5L for a descriptive overview of quality of life, which can be used for economic evaluation (60).
- Functional capacity as measured by the six-minute walk test (6MWT) (61). The 6MWT is a safe, valid and reliable measure of functional capacity in cancer populations (62). All participants will perform a standardised, self-paced 6MWT in a 30-metre long corridor. Each participant will be instructed to walk as far as they can for six minutes. Participants will be allowed to stop at any time. The distance covered (to the nearest metre) after the six minutes will be measured. A cut off value of 400m has been used to determine a participant's level of need for physical activity prehabilitation intervention. This is in line with a study by Kasymjanova et al (63) on the prognostic value of the 6MWT in advanced non-small cell lung cancer in patients undergoing chemotherapy.
- Handgrip strength as measured by a handgrip dynamometer (64). Grip strength has shown to be correlated with overall health status (65–69). Each participant will be instructed to sit in a chair and bend the elbow of their non-dominant arm at a 90-degree angle. The lead researcher will ask the participant to squeeze the device as hard as they can. Each participant will repeat this three times and the average will be recorded. A cut off value of 27kg for men and 16kg for women has been used to determine a participant's level of need for nutritional prehabilitation intervention. This is based on the revised European consensus guidelines on the definition and diagnosis of sarcopenia (70).

To tailor the intervention, the above outcome measures will be assessed prior to starting treatment and before the personalised prehabilitation programme begins (baseline), at week three and at week six of a participant's oncological treatment (end point).

For peer review only - http://bmjopen.bmj.com/site/about/guidelines.xhtml

For peer review only

Demographic and clinical data including age, gender, level of education achieved, ethnicity, employment status, level of social support (measured using the Duke-University of North Carolina Functional Social Support Questionnaire; DUFSSQ) (71), diagnosis, staging, treatment and co-morbidities (measured using the Adult Co-morbidity Evaluation (ACE) 27 score) (72) will be collected at baseline.

Training

The lead researcher is a highly specialised oncology dietitian trained in advanced communication and psychological skills (73) to manage complex conversations with patients and help recognise and respond to psychological distress. The lead researcher is familiar with using a handgrip dynamometer from previous experience in clinical practice. Training in conducting the 6MWT has been provided by a specialist oncology physiotherapist.

Patient and public involvement (PPI)

Four patients (and a spouse) who had recently completed chemotherapy or radiotherapy treatment for lung cancer were consulted on the personalised prehabilitation programme via a focus group. This was separate to focus groups used in the initial co-design process to develop the personalised prehabilitation programme, which used a three-step approach. See supplemental material file 1. Members of the focus group were very positive about the programme, with many patients reporting that they found having a strong mind-set, staying active and eating well helped them get through treatment. These are key components of the personalised prehabilitation programme. The focus group also reviewed the study questionnaires and patient documentation. Patients and their relatives expressed the benefits of having a weekly telephone call to discuss their concerns and reported that the questionnaires and daily diary would make them feel supported, as the information would be tailored to them individually.

All participants who participate in the study will be offered the opportunity to provide feedback on their experience of the personalised prehabilitation programme via the use of a short free text questionnaire. This feedback will help inform future programme adjustments and have the potential to improve patient experience.

Data collection and management

Data will be collected and stored according to the Data Protection Act (2018) and in line with General Data Protection Regulation (GDPR).

All data included in this study will be kept as part of the participants clinical care record on a secure password protected electronic patient record system at the study hospital.

A subset of this data will be extracted and entered into a case report form, which will be kept on a secure password protected database at the study hospital and only accessed by the research team. This data will be pseudonymised and all participants will be given a unique study number.

Statistical analysis plan

This study includes a small cohort of lung cancer participants from a single London teaching hospital. There are no groups to detect differences between, therefore, no formal tests of statistical significance will be undertaken.

Descriptive statistics will be used to analyse baseline demographic and clinical data.

Continuous measures will be reported as means, standard deviations and/or medians along

For peer review only - http://bmjopen.bmj.com/site/about/guidelines.xhtml

with confidence intervals, while categorical data will be reported as percentages. Missing data will be reported where applicable.

Inductive thematic analysis will be used to analyse qualitative data from the short free text questionnaire regarding patient experience.

Feasibility will be determined if the a priori thresholds for feasibility have been met.

The data collected will be used to inform the design of a larger randomised pilot study.

Strengths and limitations

This study uses a novel way of delivering a multi-modal prehabilitation programme through the use of a personalised, inclusive research approach that aligns with patient values, needs and functional goals, with interventions tailored to a participant's capabilities. The personalised prehabilitation programme provides a structure and process for personalising prehabilitation that is person-centred and underpinned by a conceptual framework, which is consistent with psychological adjustment theory. This could be key to empower patients to maintain activity through the longer, more variable context of peri-oncological prehabilitation. This study will help address key uncertainties, which need to be resolved before implementation, including engagement and adherence to prehabilitation interventions in lung cancer patients receiving chemotherapy, radiotherapy or immunotherapy treatment.

This study is limited by (a) lack of a comparison group (b) the small sample size used (c) self-reported adherence to the prehabilitation interventions and (d) the lead researcher also being the clinician delivering the interventions and performing assessments. In order to mitigate potential bias regarding the latter, where possible, aspects of the programme have been pre-defined.

Ethics and dissemination

Ethical approval was obtained from the Health Research Authority (HRA) on 13th April 2022 (reference number 22/PR/0390) prior to the start of the study. Any modifications will be approved by the HRA. This study has been registered with ClinicalTrials.gov (NCT: NCT05318807). Imperial College Healthcare NHS Trust is the sponsor of the study.

Monitoring and auditing will be conducted in accordance with the sponsor's policies and procedures. Results of this study will be disseminated to academics, stakeholders, cancer alliances, relevant charities and to the public and people with cancer. This will be through publication in peer-reviewed articles, presentations at scientific conferences, social media and collaboration with patient and public involvement representatives.

Author contributions

KWM is the principal investigator. KWM devised the clinical question, applied for funding and research ethics and was involved in the conception and design of the study and protocol. AK contributed to the study design and methods. AK and CU were involved in the conception and design of the study. LJ and MW contributed to all versions of the protocol. AK, CU and MW provided their expertise in cancer care and contributed to the knowledge needed for prehabilitation. CU, LJ and MW provided supervision and mentorship. All authors read and approved the final protocol.

For peer review only - http://bmjopen.bmj.com/site/about/guidelines.xhtml

Funding

This work was supported by Health Education England and the National Institute for Health and Care Research as part of an ICA Pre-doctoral Clinical and Practitioner Academic Fellowship (Grant award number: NIHR302680).

Competing interests

The authors declare that they have no competing interests.

References

1. Berkel AEM, Bongers BC, van Kamp MJS, Kotte H, Weltevreden P, de Jongh FHC, et al. The effects of prehabilitation versus usual care to reduce postoperative complications in high-risk patients with colorectal cancer or dysplasia scheduled for elective colorectal resection: study protocol of a randomized controlled trial. BMC Gastroenterol. 2018 Dec;18(1):29.

2. van Rooijen S, Carli F, Dalton S, Thomas G, Bojesen R, Le Guen M, et al. Multimodal prehabilitation in colorectal cancer patients to improve functional capacity and reduce postoperative complications: the first international randomized controlled trial for multimodal prehabilitation. BMC Cancer. 2019 Dec;19(1):98.

3. Moore J, Merchant Z, Rowlinson K, McEwan K, Evison M, Faulkner G, et al. Implementing a system-wide cancer prehabilitation programme: The journey of Greater Manchester's 'Prehab4cancer.' Eur J Surg Oncol. 2021 Mar;47(3):524–32.

4. Perry R, Herbert G, Atkinson C, England C, Northstone K, Baos S, et al. Pre-admission interventions (prehabilitation) to improve outcome after major elective surgery: a systematic review and meta-analysis. BMJ Open. 2021 Sep;11(9):e050806.

5. Molenaar CJ, van Rooijen SJ, Fokkenrood HJ, Roumen RM, Janssen L, Slooter GD. Prehabilitation versus no prehabilitation to improve functional capacity, reduce postoperative complications and improve quality of life in colorectal cancer surgery. Cochrane Colorectal Group, editor. Cochrane Database Syst Rev [Internet]. 2022 May 19 [cited 2023 Jan 26];2022(5). Available from: http://doi.wiley.com/10.1002/14651858.CD013259.pub2

6. Gillis C, Buhler K, Bresee L, Carli F, Gramlich L, Culos-Reed N, et al. Effects of Nutritional Prehabilitation, With and Without Exercise, on Outcomes of Patients Who Undergo Colorectal Surgery: A Systematic Review and Meta-analysis. Gastroenterology. 2018 Aug;155(2):391-410.e4.

7. Lambert JE, Hayes LD, Keegan TJ, Subar DA, Gaffney CJ. The Impact of Prehabilitation on Patient Outcomes in Hepatobiliary, Colorectal, and Upper Gastrointestinal Cancer Surgery: A PRISMA-Accordant Meta-analysis. Ann Surg. 2021 Jul;274(1):70–7.

8. Waterland JL, McCourt O, Edbrooke L, Granger CL, Ismail H, Riedel B, et al. Efficacy of Prehabilitation Including Exercise on Postoperative Outcomes Following Abdominal Cancer Surgery: A Systematic Review and Meta-Analysis. Front Surg. 2021 Mar 19;8:628848.

For peer review only

For peer review only - http://bmjopen.bmj.com/site/about/guidelines.xhtml

9. Treanor C, Kyaw T, Donnelly M. An international review and meta-analysis of prehabilitation compared to usual care for cancer patients. J Cancer Surviv. 2018 Feb;12(1):64–73.

10. Fulop A, Lakatos L, Susztak N, Szijarto A, Banky B. The effect of trimodal prehabilitation on the physical and psychological health of patients undergoing colorectal surgery: a randomised clinical trial. Anaesthesia. 2021 Jan;76(1):82–90.

11. Wu F, Rotimi O, Laza-Cagigas R, Rampal T. The Feasibility and Effects of a Telehealth-Delivered Home-Based Prehabilitation Program for Cancer Patients during the Pandemic. Curr Oncol. 2021 Jun 17;28(3):2248–59.

12. Allen SK, Brown V, White D, King D, Hunt J, Wainwright J, et al. Multimodal Prehabilitation During Neoadjuvant Therapy Prior to Esophagogastric Cancer Resection: Effect on Cardiopulmonary Exercise Test Performance, Muscle Mass and Quality of Life—A Pilot Randomized Clinical Trial. Ann Surg Oncol. 2022 Mar;29(3):1839–50.

13. Cancer Research UK. Cancer Research UK. Cancer treatment statistics 2013-2016 [Internet]. 2013 2016 [cited 2022 Oct 3]. Available from: https://www.cancerresearchuk.org/health-professional/cancer-statistics/treatment

14. West MA, Loughney L, Lythgoe D, Barben CP, Sripadam R, Kemp GJ, et al. Effect of prehabilitation on objectively measured physical fitness after neoadjuvant treatment in preoperative rectal cancer patients: a blinded interventional pilot study. Br J Anaesth. 2015 Feb;114(2):244–51.

15. Christensen JF, Simonsen C, Banck-Petersen A, Thorsen-Streit S, Herrstedt A, Djurhuus SS, et al. Safety and feasibility of preoperative exercise training during neoadjuvant treatment before surgery for adenocarcinoma of the gastro-oesophageal junction: Preoperative exercise training during neoadjuvant treatment for gastro-oesophageal junction adenocarcinoma. BJS Open. 2019 Feb;3(1):74–84.

16. Dewberry LC, Wingrove LJ, Marsh MD, Glode AE, Schefter TE, Leong S, et al. Pilot Prehabilitation Program for Patients With Esophageal Cancer During Neoadjuvant Therapy and Surgery. J Surg Res. 2019 Mar;235:66–72.

17. Halliday LJ, Doganay E, Wynter-Blyth V, Osborn H, Buckley J, Moorthy K. Adherence to Pre-operative Exercise and the Response to Prehabilitation in Oesophageal Cancer Patients. J Gastrointest Surg. 2021 Apr;25(4):890–9.

18. Ligthart-Melis GC, Weijs PJM, te Boveldt ND, Buskermolen S, Earthman CP, Verheul HMW, et al. Dietician-delivered intensive nutritional support is associated with a decrease in severe postoperative complications after surgery in patients with esophageal cancer: INS limits postoperative complications. Dis Esophagus. 2013 Aug;26(6):587–93.

19. Schmid S, Minnella EM, Pilon Y, Rokah M, Rayes R, Najmeh S, et al. Neoadjuvant Prehabilitation Therapy for Locally Advanced Non–Small-Cell Lung Cancer: Optimizing Outcomes Throughout the Trajectory of Care. Clin Lung Cancer. 2022 Nov;23(7):593–9.

20. Cancer Research UK. Cancer Research UK. Lung cancer treatment statistics 2013-2014 [Internet]. 2013 2014 [cited 2022 Oct 17]. Available from: https://www.cancerresearchuk.org/health-professional/cancer-statistics/statistics-by-cancer-type/lung-cancer/diagnosis-and-treatment#heading-Three

For peer review only - http://bmjopen.bmj.com/site/about/guidelines.xhtml

21. Cancer Research UK. Cancer Research UK. Lung cancer mortality statistics 2017-2019 [Internet]. 2017 2019 [cited 2022 Oct 17]. Available from: https://www.cancerresearchuk.org/health-professional/cancer-statistics/statistics-by-cancer-type/lung-cancer#heading-Five

22. Laurent H, Aubreton S, Galvaing G, Pereira B, Merle P, Richard R, et al. Preoperative respiratory muscle endurance training improves ventilatory capacity and prevents pulmonary postoperative complications after lung surgery. Eur J Phys Rehabil Med [Internet]. 2020 Feb [cited 2022 Oct 17];56(1). Available from: https://www.minervamedica.it/index2.php?show=R33Y2020N01A0073

23. Liu Z, Qiu T, Pei L, Zhang Y, Xu L, Cui Y, et al. Two-Week Multimodal Prehabilitation Program Improves Perioperative Functional Capability in Patients Undergoing Thoracoscopic Lobectomy for Lung Cancer: A Randomized Controlled Trial. Anesth Analg. 2020 Sep;131(3):840–9.

24. Ricketts WM, Bollard K, Streets E, Hutton K, Hornby C, Lau K. Feasibility of setting up a pre-operative optimisation 'pre-hab' service for lung cancer surgery in the UK. Perioper Med. 2020 Dec;9(1):14.

25. Avancini A, Cavallo A, Trestini I, Tregnago D, Belluomini L, Crisafulli E, et al. Exercise prehabilitation in lung cancer: Getting stronger to recover faster. Eur J Surg Oncol. 2021 Aug;47(8):1847–55.

26. Bradley P, Merchant Z, Rowlinson-Groves K, Grundy S, Al-Najjar H, Brown L, et al. P212 Prehab4Cancer: an innovative regional lung cancer prehabilitation service. In: The lung cancer diagnostic journey [Internet]. BMJ Publishing Group Ltd and British Thoracic Society; 2021 [cited 2022 Oct 17]. p. A204.2-A205. Available from: https://thorax.bmj.com/lookup/doi/10.1136/thorax-2020-BTSabstracts.357

27. Ferreira V, Minnella EM, Awasthi R, Gamsa A, Ferri L, Mulder D, et al. Multimodal Prehabilitation for Lung Cancer Surgery: A Randomized Controlled Trial. Ann Thorac Surg. 2021 Nov;112(5):1600–8.

28. Goldsmith I, Chesterfield-Thomas G, Toghill H. Pre-treatment optimization with pulmonary rehabilitation in lung cancer: Making the inoperable patients operable. EClinicalMedicine. 2021 Jan;31:100663.

29. Bradley P, Merchant Z, Rowlinson-Groves K, Taylor M, Moore J, Evison M. Feasibility and outcomes of a real-world regional lung cancer prehabilitation programme in the UK. Br J Anaesth. 2022 Jul;S0007091222002951.

30. Granger C, Cavalheri V. Preoperative exercise training for people with non-small cell lung cancer. Cochrane Lung Cancer Group, editor. Cochrane Database Syst Rev [Internet]. 2022 Sep 28 [cited 2023 Jan 11];2022(9). Available from: http://doi.wiley.com/10.1002/14651858.CD012020.pub3

31. Ferreira V, Agnihotram RV, Bergdahl A, van Rooijen SJ, Awasthi R, Carli F, et al. Maximizing patient adherence to prehabilitation: what do the patients say? Support Care Cancer. 2018 Aug;26(8):2717–23.

32. Boutillier CL, Archer S, Barry C, King A, Mansfield L, Urch C. Conceptual framework for living with and beyond cancer: A systematic review and narrative synthesis. Psychooncology. 2019;28(5):948–59.

For peer review only - http://bmjopen.bmj.com/site/about/guidelines.xhtml

33. Grimmett C, Bradbury K, Dalton SO, Fecher-Jones I, Hoedjes M, Varkonyi-Sepp J, et al. The Role of Behavioral Science in Personalized Multimodal Prehabilitation in Cancer. Front Psychol. 2021;12:261.

34. Bayly J, Edwards BM, Peat N, Warwick G, Hennig IM, Arora A, et al. Developing an integrated rehabilitation model for thoracic cancer services: views of patients, informal carers and clinicians. Pilot Feasibility Stud. 2018 Dec;4(1):160.

35. Bayly J, Fettes L, Douglas E, Teixiera MJ, Peat N, Tunnard I, et al. Short-term integrated rehabilitation for people with newly diagnosed thoracic cancer: a multi-centre randomized controlled feasibility trial. Clin Rehabil. 2020 Feb;34(2):205–19.

36. Macmillan Cancer Support, NIHR Cancer and Nutrition Collaboration, Royal College of Anaesthetists. Principles and guidance for prehabilitation within the management and support of people with cancer [Internet]. Macmillan Cancer Support; 2019 [cited 2021 Oct 27]. Available from: https://www.macmillan.org.uk/healthcare-professionals/news-and-resources/guides/principles-and-guidance-for-prehabilitation

37. Brennan J. Adjustment to cancer?coping or personal transition? Psychooncology. 2001 Jan;10(1):1–18.

38. Hickmann E, Richter P, Schlieter H. All together now – patient engagement, patient empowerment, and associated terms in personal healthcare. BMC Health Serv Res. 2022 Sep 2;22(1):1116.

39. Eldridge SM, Chan CL, Campbell MJ, Bond CM, Hopewell S, Thabane L, et al. CONSORT 2010 statement: extension to randomised pilot and feasibility trials. BMJ. 2016 Oct 24;i5239.

40. Eldridge SM, Lancaster GA, Campbell MJ, Thabane L, Hopewell S, Coleman CL, et al. Defining Feasibility and Pilot Studies in Preparation for Randomised Controlled Trials: Development of a Conceptual Framework. Lazzeri C, editor. PLOS ONE. 2016 Mar 15;11(3):e0150205.

41. Pearson N, Naylor PJ, Ashe MC, Fernandez M, Yoong SL, Wolfenden L. Guidance for conducting feasibility and pilot studies for implementation trials. Pilot Feasibility Stud. 2020 Dec;6(1):167.

42. Orsmond GI, Cohn ES. The Distinctive Features of a Feasibility Study: Objectives and Guiding Questions. OTJR Occup Particip Health. 2015 Jul;35(3):169–77.

43. Skivington K, Matthews L, Simpson SA, Craig P, Baird J, Blazeby JM, et al. A new framework for developing and evaluating complex interventions: update of Medical Research Council guidance. BMJ. 2021 Sep 30;n2061.

44. Julious SA. Sample size of 12 per group rule of thumb for a pilot study. Pharm Stat. 2005 Oct;4(4):287–91.

45. Sim J, Lewis M. The size of a pilot study for a clinical trial should be calculated in relation to considerations of precision and efficiency. J Clin Epidemiol. 2012 Mar;65(3):301–8.

46. Heitzmann CA, Merluzzi TV, Jean-Pierre P, Roscoe JA, Kirsh KL, Passik SD. Assessing self-efficacy for coping with cancer: development and psychometric analysis of the brief version of the Cancer Behavior Inventory (CBI-B). Psychooncology. 2011 Mar;20(3):302–12.

For peer review only - http://bmjopen.bmj.com/site/about/guidelines.xhtml

47. Hibbard JH, Mahoney ER, Stockard J, Tusler M. Development and Testing of a Short Form of the Patient Activation Measure. Health Serv Res. 2005 Dec;40(6p1):1918–30.

48. Godin G. The Godin-Shephard Leisure-Time Physical Activity Questionnaire. Health Fit J Can. 2011 Jan 30;18-22 Pages.

49. Ottery F. Definition of standardized nutritional assessment and interventional pathways in oncology. Nutrition. 1996 Jan;12:S15–9.

50. Harju E, Michel G, Roser K. A systematic review on the use of the emotion thermometer in individuals diagnosed with cancer. Psychooncology. 2019 Sep;28(9):1803–18.

51. Ruta DA, Garratt AM, Leng M, Russell IT, MacDonald LM. A New Approach to the Measurement of Quality of Life: The Patient-Generated Index. Med Care. 1994 Nov;32(11):1109–26.

52. Kwasnicka D, Dombrowski SU, White M, Sniehotta F. Theoretical explanations for maintenance of behaviour change: a systematic review of behaviour theories. Health Psychol Rev. 2016 Jul 2;10(3):277–96.

53. Tang JA, Oh T, Scheer JK, Parsa AT. The current trend of administering a patient-generated index in the oncological setting: a systematic review. Oncol Rev [Internet]. 2014 Jun 30 [cited 2022 Oct 17]; Available from: https://www.frontierspartnerships.org/articles/10.4081/oncol.2014.245

54. Thabane L, Ma J, Chu R, Cheng J, Ismaila A, Rios LP, et al. A tutorial on pilot studies: the what, why and how. BMC Med Res Methodol. 2010 Jan 6;10(1):1.

55. Avery KNL, Williamson PR, Gamble C, O'Connell Francischetto E, Metcalfe C, Davidson P, et al. Informing efficient randomised controlled trials: exploration of challenges in developing progression criteria for internal pilot studies. BMJ Open. 2017 Feb;7(2):e013537.

56. Hallingberg B, Turley R, Segrott J, Wight D, Craig P, Moore L, et al. Exploratory studies to decide whether and how to proceed with full-scale evaluations of public health interventions: a systematic review of guidance. Pilot Feasibility Stud. 2018 Dec;4(1):104.

57. Mellor K, Albury C, Dutton SJ, Eldridge S, Hopewell S. Recommendations for progression criteria during external randomised pilot trial design, conduct, analysis and reporting. Pilot Feasibility Stud. 2023 Apr 15;9(1):59, s40814-023-01291–5.

58. Faithfull S, Turner L, Poole K, Joy M, Manders R, Weprin J, et al. Prehabilitation for adults diagnosed with cancer: A systematic review of long-term physical function, nutrition and patient-reported outcomes. Eur J Cancer Care (Engl) [Internet]. 2019 Jul [cited 2021 Oct 27];28(4). Available from: https://onlinelibrary.wiley.com/doi/10.1111/ecc.13023

59. Aaronson NK, Ahmedzai S, Bergman B, Bullinger M, Cull A, Duez NJ, et al. The European Organization for Research and Treatment of Cancer QLQ-C30: A Quality-of-Life Instrument for Use in International Clinical Trials in Oncology. JNCI J Natl Cancer Inst. 1993 Mar 3;85(5):365–76.

60. Herdman M, Gudex C, Lloyd A, Janssen Mf, Kind P, Parkin D, et al. Development and preliminary testing of the new five-level version of EQ-5D (EQ-5D-5L). Qual Life Res. 2011 Dec;20(10):1727–36.

For peer review only - http://bmjopen.bmj.com/site/about/guidelines.xhtml

61. ATS Statement: Guidelines for the Six-Minute Walk Test. Am J Respir Crit Care Med. 2002 Jul 1;166(1):111–7.

62. Schmidt K, Vogt L, Thiel C, Jäger E, Banzer W. Validity of the Six-Minute Walk Test in Cancer Patients. Int J Sports Med. 2013 Feb 26;34(07):631–6.

63. Kasymjanova G, Correa JA, Kreisman H, Dajczman E, Pepe C, Dobson S, et al. Prognostic Value of the Six-Minute Walk in Advanced Non-small Cell Lung Cancer. J Thorac Oncol. 2009 May;4(5):602–7.

64. Spruit MA, Sillen MJH, Groenen MTJ, Wouters EFM, Franssen FME. New Normative Values for Handgrip Strength: Results From the UK Biobank. J Am Med Dir Assoc. 2013 Oct;14(10):775.e5-775.e11.

65. Musalek C, Kirchengast S. Grip Strength as an Indicator of Health-Related Quality of Life in Old Age—A Pilot Study. Int J Environ Res Public Health. 2017 Nov 24;14(12):1447.

66. Celis-Morales C, Welsh P, Lyall D, Steell L, Petermann F, Anderson J, et al. Associations of grip strength with cardiovascular, respiratory, and cancer outcomes and all cause mortality: prospective cohort study of half a million UK Biobank participants. 2018 Aug 5;361:1–10.

67. Bohannon RW. Grip Strength: An Indispensable Biomarker For Older Adults. Clin Interv Aging. 2019 Oct;Volume 14:1681–91.

68. Soysal P, Hurst C, Demurtas J, Firth J, Howden R, Yang L, et al. Handgrip strength and health outcomes: Umbrella review of systematic reviews with meta-analyses of observational studies. J Sport Health Sci. 2021 May;10(3):290–5.

69. Jiang R, Westwater ML, Noble S, Rosenblatt M, Dai W, Qi S, et al. Associations between grip strength, brain structure, and mental health in > 40,000 participants from the UK Biobank. BMC Med. 2022 Sep 9;20(1):286.

70. Cruz-Jentoft AJ, Bahat G, Bauer J, Boirie Y, Bruyère O, Cederholm T, et al. Sarcopenia: revised European consensus on definition and diagnosis. Age Ageing. 2019 Jan 1;48(1):16–31.

71. Saracino R, Kolva E, Rosenfeld B, Breitbart W. Measuring social support in patients with advanced medical illnesses: An analysis of the Duke–UNC Functional Social Support Questionnaire. Palliat Support Care. 2015 Oct;13(5):1153–63.

72. Kallogjeri D, Piccirillo JF, Spitznagel EL, Steyerberg EW. Comparison of scoring methods for ACE-27: Simpler is better. J Geriatr Oncol. 2012 Jul;3(3):238–45.

73. London Cancer Alliance. Guidance for Level Two Healthcare Workers and Level Three/Four Supervisors [Internet]. London Cancer Alliance; 2015. Available from: https://rmpartners.nhs.uk/wp-content/uploads/2017/03/lca-guidance-for-level-2-healthcare-workers-and-level-3_4-supervisors-september-2015.pdf

For peer review only

For peer review only - http://bmjopen.bmj.com/site/about/guidelines.xhtml

Figure legends

Figure 1. Diagrammatic representation of the Adversity, Restoration and Compatibility (ARC) model of the experience of adjusting to living with and beyond cancer. The waves and curves reflect the fluidity and non-linear nature of process, with the sizes reducing as one adjusts to living with and beyond cancer.

Figures 2a, 2b and 2c. Examples of the models used in the personalised prehabilitation programme for psychological wellbeing, nutrition and physical activity.

For peer review only

For peer review only - http://bmjopen.bmj.com/site/about/guidelines.xhtml

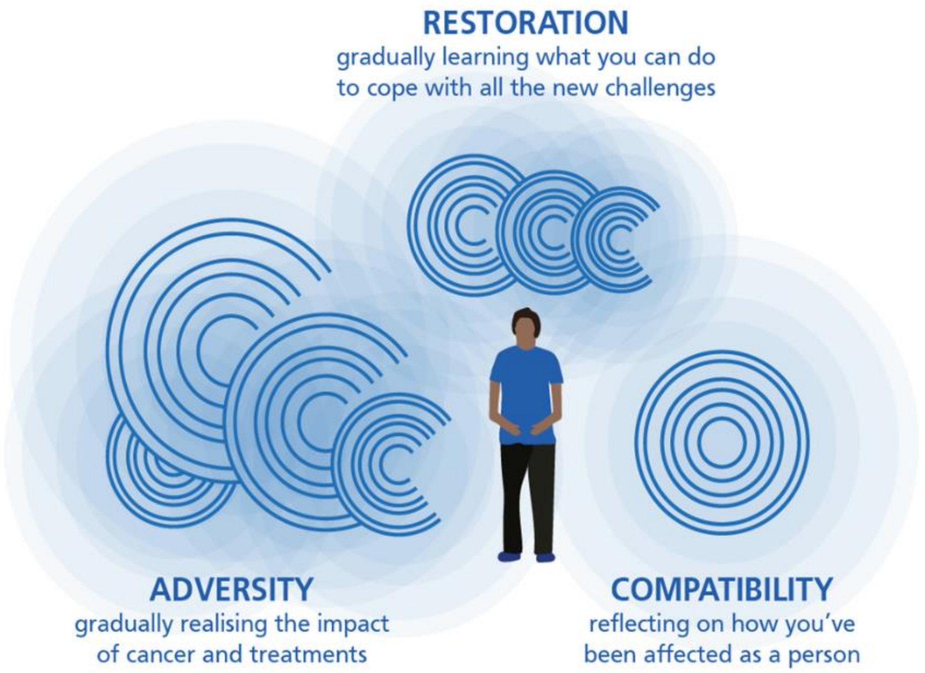

Figure 1. Diagrammatic representation of the Adversity, Restoration and Compatibility (ARC) model of the experience of adjusting to living with and beyond cancer. The waves and curves reflect the fluidity and non-linear nature of process, with the sizes reducing as one adjusts to living with and beyond cancer.

For peer review only

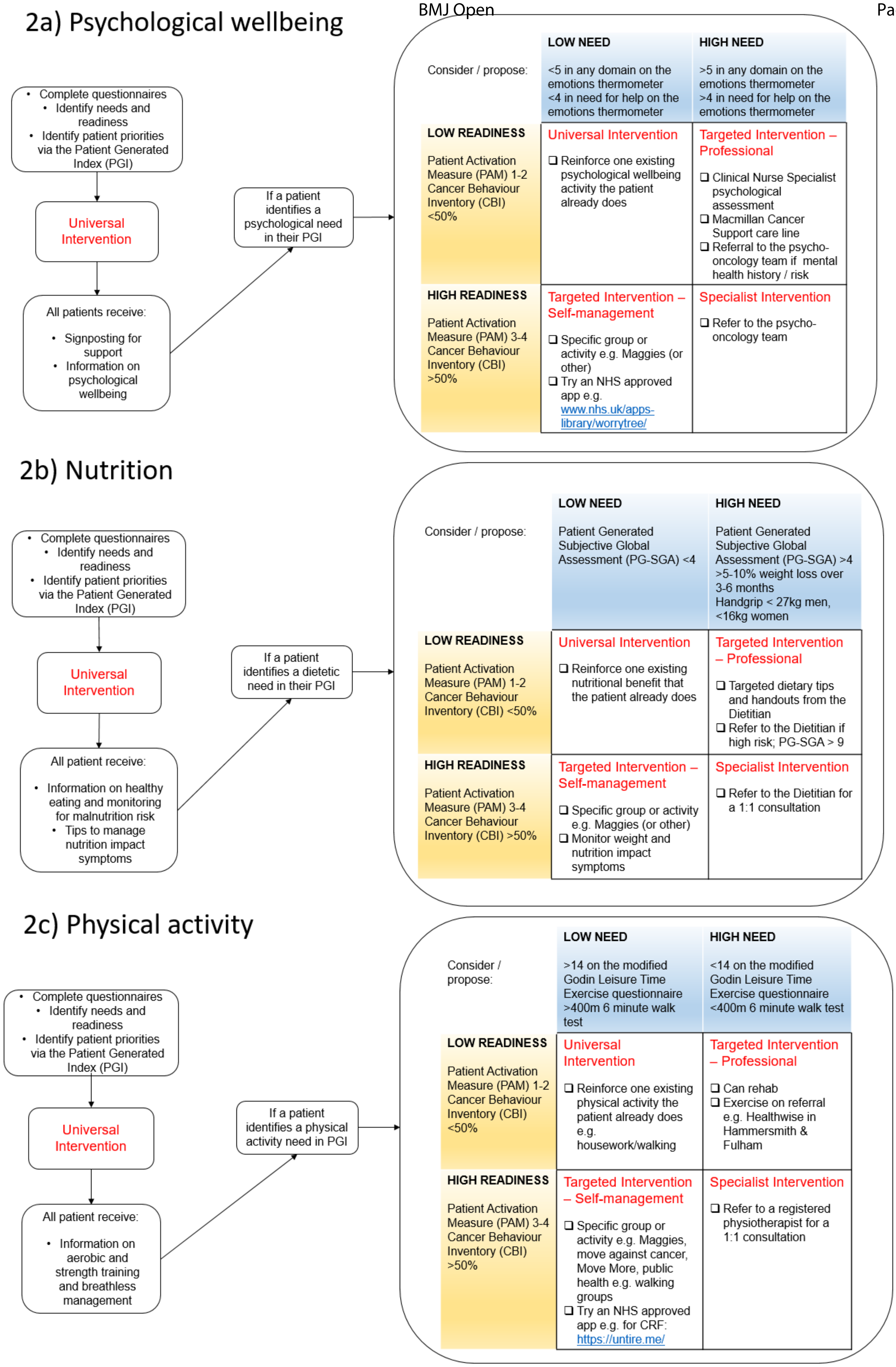

Figures 2a, 2b and 2c. Examples of the models used in the personalised prehabilitation programme for psychological wellbeing, nutrition and physical activity.

Co-design approach used for the development of the personalised prehabilitation programme.

A participatory action research approach using qualitative group interviews to develop the intervention, as guided by the MRC framework was undertaken in partnership with a single London teaching hospital and Maggie's cancer centre (an independent charity that provides free cancer support, workshops and courses to help anyone affected by cancer).

Participants who had completed oncological treatment for lung cancer were invited to an informal face-to-face group discussion. Participants were invited either in person at the monthly lung cancer support group held at Maggie's cancer centre or by their clinical oncologist or clinical nurse specialist at a follow up outpatient appointment.

Nine participants were invited to the informal group discussion and a total of six participants attended. The main reason for non-attendance was a lack of time. Participants were invited to share their thoughts on what they would want from a prehabilitation programme and through facilitated discussion, participant priorities were agreed. Many participants remarked on the importance of individualisation, being seeing face-to-face and the programme being endorsed by all members of the team.

Separately, five healthcare professionals from a range of different specialties including clinical psychology, physiotherapy, dietetics and cancer services met to discuss their ideas about what a prehabilitation programme for people with lung cancer undergoing chemotherapy or radiotherapy treatment should look like.

A joint meeting was then held with both participants and healthcare professionals to share thoughts and ideas and an agreement was sought on what an ideal prehabilitation programme should look like.

Table 2 | CONSORT checklist of information to include when reporting a pilot trial

Section/topic and item No	Standard checklist item	Extension for pilot trials	Page No where item is reported
**Title and abstract**			
1a	Identification as a randomised trial in the title	Identification as a pilot or feasibility randomised trial in the title	Page 1
1b	Structured summary of trial design, methods, results, and conclusions (for specific guidance see CONSORT for abstracts)	Structured summary of pilot trial design, methods, results, and conclusions (for specific guidance see CONSORT abstract extension for pilot trials)	Page 1
**Introduction**			
Background and objectives:			
2a	Scientific background and explanation of rationale	Scientific background and explanation of rationale for future definitive trial, and reasons for randomised pilot trial	Pages 2-3
2b	Specific objectives or hypotheses	Specific objectives or research questions for pilot trial	Page 3
**Methods**			
Trial design:			
3a	Description of trial design (such as parallel, factorial) including allocation ratio	Description of pilot trial design (such as parallel, factorial) including allocation ratio	Page 3
3b	Important changes to methods after trial commencement (such as eligibility criteria), with reasons	Important changes to methods after pilot trial commencement (such as eligibility criteria), with reasons	N/A
Participants:			
4a	Eligibility criteria for participants		Page 3
4b	Settings and locations where the data were collected		Page 4
4c		How participants were identified and consented	Page 4
Interventions:			
5	The interventions for each group with sufficient details to allow replication, including how and when they were actually administered		Pages 4-5
Outcomes:			
6a	Completely defined prespecified primary and secondary outcome measures, including how and when they were assessed	Completely defined prespecified assessments or measurements to address each pilot trial objective specified in 2b, including how and when they were assessed	Pages 6-7
6b	Any changes to trial outcomes after the trial commenced, with reasons	Any changes to pilot trial assessments or measurements after the pilot trial commenced, with reasons	N/A
6c		If applicable, prespecified criteria used to judge whether, or how, to proceed with future definitive trial	Page 6
Sample size:			
7a	How sample size was determined	Rationale for numbers in the pilot trial	Page 4
7b	When applicable, explanation of any interim analyses and stopping guidelines		N/A
Randomisation:			
Sequence generation:			
8a	Method used to generate the random allocation sequence		N/A
8b	Type of randomisation; details of any restriction (such as blocking and block size)	Type of randomisation(s); details of any restriction (such as blocking and block size)	N/A
Allocation concealment mechanism:			
9	Mechanism used to implement the random allocation sequence (such as sequentially numbered containers), describing any steps taken to conceal the sequence until interventions were assigned		N/A
Implementation:			
10	Who generated the random allocation sequence, enrolled participants, and assigned participants to interventions		N/A
Blinding:			
11a	If done, who was blinded after assignment to interventions (eg, participants, care providers, those assessing outcomes) and how		N/A
11b	If relevant, description of the similarity of interventions		N/A
Analytical methods:			
12a	Statistical methods used to compare groups for primary and secondary outcomes	Methods used to address each pilot trial objective whether qualitative or quantitative	Pages 8-9
12b	Methods for additional analyses, such as subgroup analyses and adjusted analyses	Not applicable	N/A

Table 2 | CONSORT checklist of information to include when reporting a pilot trial

Section/topic and item No	Standard checklist item	Extension for pilot trials	Page No where item is reported
**Results**			
Participant flow (a diagram is strongly recommended):			
13a	For each group, the numbers of participants who were randomly assigned, received intended treatment, and were analysed for the primary outcome	For each group, the numbers of participants who were approached and/or assessed for eligibility, randomly assigned, received intended treatment, and were assessed for each objective	N/A – protocol paper
13b	For each group, losses and exclusions after randomisation, together with reasons		N/A – protocol paper
Recruitment:			
14a	Dates defining the periods of recruitment and follow-up		Page 4
14b	Why the trial ended or was stopped	Why the pilot trial ended or was stopped	N/A
Baseline data:			
15	A table showing baseline demographic and clinical characteristics for each group		N/A – protocol paper
Numbers analysed:			
16	For each group, number of participants (denominator) included in each analysis and whether the analysis was by original assigned groups	For each objective, number of participants (denominator) included in each analysis. If relevant, these numbers should be by randomised group	N/A – protocol paper
Outcomes and estimation:			
17a	For each primary and secondary outcome, results for each group, and the estimated effect size and its precision (such as 95% confidence interval)	For each objective, results including expressions of uncertainty (such as 95% confidence interval) for any estimates. If relevant, these results should be by randomised group	N/A – protocol paper
17b	For binary outcomes, presentation of both absolute and relative effect sizes is recommended	Not applicable	N/A
Ancillary analyses:			
18	Results of any other analyses performed, including subgroup analyses and adjusted analyses, distinguishing prespecified from exploratory	Results of any other analyses performed that could be used to inform the future definitive trial	N/A – protocol paper
Harms:			
19	All important harms or unintended effects in each group (for specific guidance see CONSORT for harms)		N/A – protocol paper
19a		If relevant, other important unintended consequences	N/A – protocol paper
**Discussion**			
Limitations:			
20	Trial limitations, addressing sources of potential bias, imprecision, and, if relevant, multiplicity of analyses	Pilot trial limitations, addressing sources of potential bias and remaining uncertainty about feasibility	Page 9
Generalisability:			
21	Generalisability (external validity, applicability) of the trial findings	Generalisability (applicability) of pilot trial methods and findings to future definitive trial and other studies	N/A – protocol paper
Interpretation:			
22	Interpretation consistent with results, balancing benefits and harms, and considering other relevant evidence	Interpretation consistent with pilot trial objectives and findings, balancing potential benefits and harms, and considering other relevant evidence	N/A – protocol paper
22a		Implications for progression from pilot to future definitive trial, including any proposed amendments	N/A – protocol paper
**Other information**			
Registration:			
23	Registration number and name of trial registry	Registration number for pilot trial and name of trial registry	Page 9
Protocol:			
24	Where the full trial protocol can be accessed, if available	Where the pilot trial protocol can be accessed, if available	N/A
Funding:			
25	Sources of funding and other support (such as supply of drugs), role of funders		Page 10
26		Ethical approval or approval by research review committee, confirmed with reference number	Page 9

SPIRIT

STANDARD PROTOCOL ITEMS: RECOMMENDATIONS FOR INTERVENTIONAL TRIALS

SPIRIT 2013 Checklist: Recommended items to address in a clinical trial protocol and related documents*

Section/item	Item No	Description	Page / line number in main document
**Administrative information**			
Title	1	Descriptive title identifying the study design, population, interventions, and, if applicable, trial acronym	Page 1
Trial registration	2a	Trial identifier and registry name. If not yet registered, name of intended registry	Page 9
	2b	All items from the World Health Organization Trial Registration Data Set	N/A
Protocol version	3	Date and version identifier	Page 1
Funding	4	Sources and types of financial, material, and other support	Page 10
Roles and responsibilities	5a	Names, affiliations, and roles of protocol contributors	Pages 1 and 9
	5b	Name and contact information for the trial sponsor	N/A
	5c	Role of study sponsor and funders, if any, in study design; collection, management, analysis, and interpretation of data; writing of the report; and the decision to submit the report for publication, including whether they will have ultimate authority over any of these activities	N/A
	5d	Composition, roles, and responsibilities of the coordinating centre, steering committee, endpoint adjudication committee, data management team, and other individuals or groups overseeing the trial, if applicable (see Item 21a for data monitoring committee)	N/A

For peer review only

**Introduction**			
Background and rationale	6a	Description of research question and justification for undertaking the trial, including summary of relevant studies (published and unpublished) examining benefits and harms for each intervention	Pages 2-3
	6b	Explanation for choice of comparators	N/A
Objectives	7	Specific objectives or hypotheses	Page 3
Trial design	8	Description of trial design including type of trial (eg, parallel group, crossover, factorial, single group), allocation ratio, and framework (eg, superiority, equivalence, noninferiority, exploratory)	Page 3
**Methods: Participants, interventions, and outcomes**			
Study setting	9	Description of study settings (eg, community clinic, academic hospital) and list of countries where data will be collected. Reference to where list of study sites can be obtained	Page 4
Eligibility criteria	10	Inclusion and exclusion criteria for participants. If applicable, eligibility criteria for study centres and individuals who will perform the interventions (eg, surgeons, psychotherapists)	Page 3
Interventions	11a	Interventions for each group with sufficient detail to allow replication, including how and when they will be administered	Pages 4-5
	11b	Criteria for discontinuing or modifying allocated interventions for a given trial participant (eg, drug dose change in response to harms, participant request, or improving/worsening disease)	See page 3 of the participant information sheet at the end of this checklist
	11c	Strategies to improve adherence to intervention protocols, and any procedures for monitoring adherence (eg, drug tablet return, laboratory tests)	See page 7 of the participant information sheet at the end of this checklist
	11d	Relevant concomitant care and interventions that are permitted or prohibited during the trial	N/A

For peer review only - http://bmjopen.bmj.com/site/about/guidelines.xhtml

Outcomes	12	Primary, secondary, and other outcomes, including the specific measurement variable (eg, systolic blood pressure), analysis metric (eg, change from baseline, final value, time to event), method of aggregation (eg, median, proportion), and time point for each outcome. Explanation of the clinical relevance of chosen efficacy and harm outcomes is strongly recommended	Pages 6-7
Participant timeline	13	Time schedule of enrolment, interventions (including any run-ins and washouts), assessments, and visits for participants. A schematic diagram is highly recommended (see Figure)	See page 6 of the participant information sheet at the end of this checklist
Sample size	14	Estimated number of participants needed to achieve study objectives and how it was determined, including clinical and statistical assumptions supporting any sample size calculations	Page 3
Recruitment	15	Strategies for achieving adequate participant enrolment to reach target sample size	Page 3
**Methods: Assignment of interventions (for controlled trials)**			
Allocation:			
Sequence generation	16a	Method of generating the allocation sequence (eg, computer-generated random numbers), and list of any factors for stratification. To reduce predictability of a random sequence, details of any planned restriction (eg, blocking) should be provided in a separate document that is unavailable to those who enrol participants or assign interventions	N/A
Allocation concealmen t mechanism	16b	Mechanism of implementing the allocation sequence (eg, central telephone; sequentially numbered, opaque, sealed envelopes), describing any steps to conceal the sequence until interventions are assigned	N/A

Implementation	16c	Who will generate the allocation sequence, who will enrol participants, and who will assign participants to interventions	N/A
Blinding (masking)	17a	Who will be blinded after assignment to interventions (eg, trial participants, care providers, outcome assessors, data analysts), and how	N/A
	17b	If blinded, circumstances under which unblinding is permissible, and procedure for revealing a participant's allocated intervention during the trial	N/A
**Methods: Data collection, management, and analysis**			
Data collection methods	18a	Plans for assessment and collection of outcome, baseline, and other trial data, including any related processes to promote data quality (eg, duplicate measurements, training of assessors) and a description of study instruments (eg, questionnaires, laboratory tests) along with their reliability and validity, if known. Reference to where data collection forms can be found, if not in the protocol	Pages 4-8
	18b	Plans to promote participant retention and complete follow-up, including list of any outcome data to be collected for participants who discontinue or deviate from intervention protocols	See page 3 of the participant information sheet at the end of this checklist
Data management	19	Plans for data entry, coding, security, and storage, including any related processes to promote data quality (eg, double data entry; range checks for data values). Reference to where details of data management procedures can be found, if not in the protocol	Page 8 and page 3 of the participant information sheet at the end of this checklist
Statistical methods	20a	Statistical methods for analysing primary and secondary outcomes. Reference to where other details of the statistical analysis plan can be found, if not in the protocol	Page 8

For peer review only - http://bmjopen.bmj.com/site/about/guidelines.xhtml

	20b	Methods for any additional analyses (eg, subgroup and adjusted analyses)	N/A
	20c	Definition of analysis population relating to protocol non-adherence (eg, as randomised analysis), and any statistical methods to handle missing data (eg, multiple imputation)	Pages 8-9
**Methods: Monitoring**			
Data monitoring	21a	Composition of data monitoring committee (DMC); summary of its role and reporting structure; statement of whether it is independent from the sponsor and competing interests; and reference to where further details about its charter can be found, if not in the protocol. Alternatively, an explanation of why a DMC is not needed	A DMC is not needed as this is a small, short-term feasibility pilot study with low risk
	21b	Description of any interim analyses and stopping guidelines, including who will have access to these interim results and make the final decision to terminate the trial	N/A
Harms	22	Plans for collecting, assessing, reporting, and managing solicited and spontaneously reported adverse events and other unintended effects of trial interventions or trial conduct	See reporting of adverse events from study protocol at the end of this checklist
Auditing	23	Frequency and procedures for auditing trial conduct, if any, and whether the process will be independent from investigators and the sponsor	N/A
**Ethics and dissemination**			
Research ethics approval	24	Plans for seeking research ethics committee/institutional review board (REC/IRB) approval	Page 9
Protocol amendments	25	Plans for communicating important protocol modifications (eg, changes to eligibility criteria, outcomes, analyses) to relevant parties (eg, investigators, REC/IRBs, trial participants, trial registries, journals, regulators)	Page 9

Consent or assent	26a	Who will obtain informed consent or assent from potential trial participants or authorised surrogates, and how (see Item 32)	The lead researcher. An example of the participant consent form can be found at the end of this checklist
	26b	Additional consent provisions for collection and use of participant data and biological specimens in ancillary studies, if applicable	N/A
Confidentiality	27	How personal information about potential and enrolled participants will be collected, shared, and maintained in order to protect confidentiality before, during, and after the trial	See pages 3-4 of the participant information sheet at the end of this checklist
Declaration of interests	28	Financial and other competing interests for principal investigators for the overall trial and each study site	Page 10
Access to data	29	Statement of who will have access to the final trial dataset, and disclosure of contractual agreements that limit such access for investigators	See page 3 of the participant information sheet at the end of this checklist
Ancillary and post-trial care	30	Provisions, if any, for ancillary and post-trial care, and for compensation to those who suffer harm from trial participation	N/A
Dissemination policy	31a	Plans for investigators and sponsor to communicate trial results to participants, healthcare professionals, the public, and other relevant groups (eg, via publication, reporting in results databases, or other data sharing arrangements), including any publication restrictions	Page 9
	31b	Authorship eligibility guidelines and any intended use of professional writers	N/A
	31c	Plans, if any, for granting public access to the full protocol, participant-level dataset, and statistical code	N/A

Appendices

For peer review only

Informed consent materials	32	Model consent form and other related documentation given to participants and authorised surrogates	See participant information sheet and example of the participant consent form at the end of this checklist
Biological specimens	33	Plans for collection, laboratory evaluation, and storage of biological specimens for genetic or molecular analysis in the current trial and for future use in ancillary studies, if applicable	N/A

*It is strongly recommended that this checklist be read in conjunction with the SPIRIT 2013 Explanation & Elaboration for important clarification on the items. Amendments to the protocol should be tracked and dated. The SPIRIT checklist is copyrighted by the SPIRIT Group under the Creative Commons "Attribution-NonCommercial-NoDerivs 3.0 Unported" license.

Imperial College Healthcare
NHS Trust

Kelly Wade-Mcbane

Imperial-lung-prehab@nhs.net

PATIENT INFORMATION SHEET

Study title

A personalised plan of diet, exercise and emotional support for patients having chemotherapy, radiotherapy or immunotherapy treatment for lung cancer.

Reviewed by: The London Bridge Research Ethics Committee

Invitation

We would like to invite you to take part in our research study. Before you decide, it is important that you understand why the research is being done and what it will involve. Please take time to read and consider this information. If anything is not clear, please ask one of the research team.

Purpose of the study

The term 'prehabilitation' refers to a period of time before treatment and often includes a combination of diet, exercise and/or wellbeing plans to help patients through their cancer treatment. The benefits of prehabilitation have been shown in patients having surgery, but there is little research into its use for patients having chemotherapy, radiotherapy or immunotherapy treatment.

This study will see if a personalised plan of diet, exercise and emotional support can be used in practice for patients having chemotherapy, radiotherapy or immunotherapy treatment for lung cancer.

This study is part of the researcher's educational higher degree.

Why have I been invited?

You have been invited to take part as you have been diagnosed with lung cancer and are about to start chemotherapy, radiotherapy or immunotherapy treatment.

Do I have to take part?

No, you do not have to take part. Your choice to take part in the study is entirely voluntary. To help with the development of the service, a member of your clinical team may ask you why you have decided not to participate. This is entirely optional and you do not have to provide a reason. Your decision about the research will not affect the medical care you receive.

For peer review only

What will happen to me if I decide to take part?

Firstly, you will be asked to provide written consent either in person or online using your personal computer. Following consent, you will be asked to complete five questionnaires on wellbeing, physical activity and diet. These questionnaires can be completed either on paper or online. These questionnaires will take approximately 30 minutes to complete.

You will then be invited to a face to face appointment which will last approximately one hour at Charing Cross Hospital. This appointment will be with the lead researcher, who is a specialist dietitian at Imperial College Healthcare NHS Trust. At this appointment, we will:

- Review your questionnaires
- Ask you to complete some further questionnaires related to quality of life, diet and activity
- Do an assessment of your walking. This will involve you walking at your own pace as far as you can in 6 minutes
- Measure your grip strength

We will then agree your personalised plan, taking into account your level of need and readiness; identified from the questionnaires and the goals you want to achieve. If your personalised plan identifies an emotional, diet or activity need, you will receive an intervention that is suited to you.

After this appointment, we will ask you to keep a daily diary to rate your symptoms, appetite, mobility and mood throughout your treatment by providing a score each day using a scale from 0-10. You can use this diary when we phone you each week to let us know how you are getting on and to talk about any days you have found particularly good or bad.

We will invite you for two further face to face appointments at the week three and week six of your chemotherapy, radiotherapy or immunotherapy treatment. At these appointments, we will repeat the questionnaires, assess your walking and measure your handgrip strength. Personalised goals will be agreed at each stage.

Please see the diagram on page six for further information.

What are the possible benefits of taking part?

We do not know if there will be any individual benefits, but the information we get might help inform future research to help improve the lives of lung cancer patients.

Are there any possible disadvantages or risks from taking part?

There are no risks from taking part in this study.

You will be asked to complete a walking test, questionnaires and keep a daily diary, which could be a burden.
The walking test involves you walking at your own pace over a 6 minute period. You can stop when you need to. Physical activity has shown to be safe in lung cancer patients and the walking test is widely used in cancer research.

For peer review only

The questionnaires are quick to complete (about 30 minutes) and will be used to personalise your care.

The daily diary is simple to use. You will be asked to provide a score each day using a scale to rate your concerns. Please see page seven for an example of the daily diary.

All research appointments will be scheduled for the same days as your clinical appointments to prevent you having to make additional trips to the hospital. Your clinical visits may be extended by approximately one hour for the research.

Will my General Practitioner (GP) be informed of my participation?

This is optional and will not affect your participation in the study. We can let your GP know if you wish and you can tell us about this in the consent form. If you wish for your GP to be informed, we will send them a written letter accompanied with a study information sheet.

Will I be reimbursed for taking part?

Yes, we offer up to £10 towards the cost of your travel if you are required to come into hospital on a day outside your usual clinical visits. Please retain your receipts. We will provide you with a travel expenses form. Once completed, you can take this to the cashier's desk at Charing Cross Hospital to be reimbursed.

What will happen if I don't want to carry on with the study?

Participation in the study is entirely voluntary and you can withdraw at any point. Withdrawal will not affect any medical care that you are receiving. If you no longer want to carry on with the research study, please email us at Imperial-lung-prehab@nhs.net. We will retain and use the information collected up to that point, but we will not collect any further information.

What happens at the end of the study?

If the results are positive, we will plan to do more testing and offer this to more people. We will provide a written summary of the findings to all participants. We will share the results in scientific journals and conferences, so that others can learn from this as well. Patients will not be identifiable from any report or publication.

Will my taking part in the study be kept confidential and what will happen to my data?

To ensure the confidentiality of all personal data, all members of the research team will follow the Imperial College Healthcare NHS Trust confidentiality policy. Any identifiable data gathered will be stored securely in accordance with the Trust policy on either a password protected Trust computer or under lock and key.

You will be given a unique study number that will be used from the start of the study. Files such as paper consent forms and questionnaires will be stored in research offices located within the clinical areas and will be secured either by lock and key or key code and accessible by only departmental staff or the Trust's security team. All paper documentation kept in these will be locked.

For peer review only

Your data will not be shared with anyone outside of your hospital treating team or local research team. Any data containing any identifiable information will only be available to the chief investigator and appropriate members of the study team on a delegation log. Your medical record will not be removed from the clinical areas.

All data will be collected according to the Data Protection Act (2018) and in line with General Data Protection Regulations (GDPR).

If you lose capacity to consent during the study, you will be withdrawn from the study. Identifiable data already collected with consent would be retained and used in the study. No further data would be collected or any other research procedures carried out on or in relation to you.

Once we have finished the study, we will keep some of the data so that we can check the results. We will write our reports in a way that no-one can work out that you took part in the study. You can find out more about how we use your information by contacting the research team at Imperial-lung-prehab@nhs.net.

How will we use information about you?

We will need to use information from you and from your medical records for this research project.

This information will include name, contact number, date of birth, contact details and proposed treatment plan held by the site and/or sponsor for the research. People will use this information to do the research or to check your records to make sure that the research is being done properly.

People who do not need to know who you are will not be able to see your name or contact details. Your data will have a code number instead.

We will keep all information about you safe and secure.

Once we have finished the study, we will keep some of the data so we can check the results. We will write our reports in a way that no-one can work out that you took part in the study.

What are your choices about how your information is used?

You can stop being part of the study at any time, without giving a reason, but we will keep information about you that we already have.

We need to manage your records in specific ways for the research to be reliable. This means that we won't be able to let you see or change the data we hold about you.

If you agree to take part in this study, you will have the option to take part in future research using your data saved from this study.

How do I find out more about how my information is used?

You can find out more about how we use your information at www.hra.nhs.uk/information-about-patients/

For peer review only

Alternatively you can:

- Ask one of the research team
- Send an email to Imperial-lung-prehab@nhs.net.
- Call us on 0203 311 7135
- Contact the Imperial Academic Health Science Centre Data Protection Officer by calling 020 7594 1862

What if we find something unexpected?

If anything of concern regarding your health comes to light during the study, your clinical team and GP will be notified.

What if something goes wrong?

Imperial College Healthcare NHS Trust holds standard NHS Hospital indemnity and insurance cover with NHS resolution for NHS Trusts in England, which apply to this study. This does not affect your legal rights to seek compensation.

If you are harmed due to someone's negligence, then you may have grounds for a legal action. Regardless of this, if you wish to complain, or have any concerns about any aspect of the way you have been treated during the course of this study then you should immediately inform the Investigator.

The normal National Health Service complaints mechanisms are also available to you. If you are still not satisfied with the response, you may contact the Imperial Academic Health Science Centre Research Governance and Integrity Team.

What if there is a problem?

If you wish to make a complaint or if you have any concerns about any aspect of the way you have been approached or treated during the course of this study, please contact the research team at Imperial-lung-prehab@nhs.net.
You can also contact the Patient Advice and Liaison Service (PALS) on 020 331 27777.
If you are still not satisfied with the response, you may contact the Imperial Academic Health Science Centre Research Governance and Integrity Team on 020 7594 1862.

Who has reviewed the study?

All research in the NHS is looked at by an independent group of people, called a Research Ethics Committee, to protect participants' interests. This study has been reviewed and given favourable ethical opinion for conduct in the NHS by The London Bridge Research Ethics Committee.

Thank you for considering to take part in this study

For peer review only

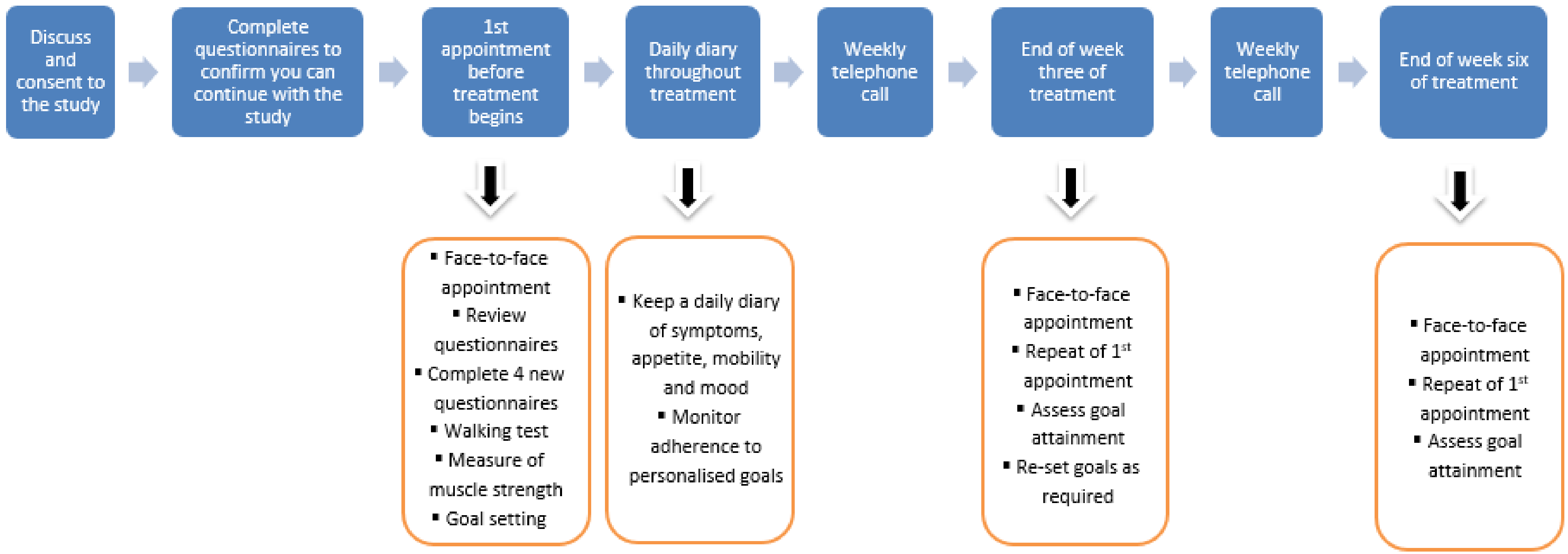
Discuss and consent to the study
Complete questionnaires to confirm you can continue with the study
1st appointment before treatment begins
Daily diary throughout treatment
Weekly telephone call
End of week three of treatment
Weekly telephone call
End of week six of treatment
▪ Face-to-face appointment
▪ Review questionnaires
▪ Complete 4 new questionnaires
▪ Walking test
▪ Measure of muscle strength
▪ Goal setting
▪ Keep a daily diary of symptoms, appetite, mobility and mood
▪ Monitor adherence to personalised goals
▪ Face-to-face appointment
▪ Repeat of 1st appointment
▪ Assess goal attainment
▪ Re-set goals as required
▪ Face-to-face appointment
▪ Repeat of 1st appointment
▪ Assess goal attainment

DIARY

Using this scale from 0-10 with 0 being no concerns and 10 being the worst it has been, please rate your symptoms, appetite, mobility and level of distress each day.

0 1 2 3 4 5 6 7 8 9 10

Cycle 1							
	Monday	Tuesday	Wednesday	Thursday	Friday	Saturday	Sunday
Symptoms							
Appetite							
Mobility							
Distress							

At the end of the week, please tell us about any days you found particularly good or difficult. Highlight these and we will talk about them.

For peer review only - http://bmjopen.bmj.com/site/about/guidelines.xhtml

Imperial College Healthcare
NHS Trust

Kelly Wade-Mcbane

Imperial-lung-prehab@nhs.net

Participant identification number | | | | | |

CONSENT FORM

Study Title

A personalised prehabilitation model for patients undergoing chemotherapy, radiotherapy or immunotherapy treatment for lung cancer: A feasibility pilot study.

Name of Researcher: Kelly Wade-Mcbane

If you agree, please initial the box

1. I confirm that I have received a copy of the patient information sheet (version 7, 06/01/2023) for this study. I have read and considered the information and have had the opportunity to ask questions and have had these answered satisfactorily	
2. I understand that my participation is voluntary. I am free to withdraw at any time without having to explain and my medical care or legal rights will not be affected	
3. I understand that relevant sections of my medical data may be looked at by responsible individuals from Imperial College Healthcare NHS Trust or by regulatory authorities where it is relevant to my taking part in this research. I give permission for these individuals to have access to my records	
4. I agree / do not agree (delete as applicable) to my general practitioner (GP) being informed of my participation in the study. This is optional and will not affect your participation in the study	
5. I understand that in any report or publication on the results of this study, my identity will remain anonymous	
6. I give / do not give (delete as applicable) consent for information collected about me to be used to support other research in the future. This is optional and will not affect your participation in the study	
7. I agree to take part in this study	

_________________________ _______________ _________________________

Name of participant Date Signature

_________________________ _______________ _________________________

Name of person taking consent Date Signature

* *1 copy for participant; 1 copy for researcher's log; 1 copy to be uploaded into patient's* electronic medical notes

For peer review only - http://bmjopen.bmj.com/site/about/guidelines.xhtml

ADVERSE EVENTS

1.1. DEFINITIONS

Adverse Event (AE): any untoward medical occurrence in a patient or clinical study subject.

Serious Adverse Event (SAE): any untoward and unexpected medical occurrence or effect that:

- **Results in death**
- **Is life-threatening** – *refers to an event in which the subject was at risk of death at the time of the event; it does not refer to an event which hypothetically might have caused death if it were more severe*
- **Requires hospitalisation, or prolongation of existing inpatients' hospitalisation**
- **Results in persistent or significant disability or incapacity**
- **Is a congenital anomaly or birth defect**

Medical judgement should be exercised in deciding whether an AE is serious in other situations. Important AEs that are not immediately life-threatening or do not result in death or hospitalisation but may jeopardise the subject or may require intervention to prevent one of the other outcomes listed in the definition above, should also be considered serious.

1.2. REPORTING PROCEDURES

All adverse events should be reported. Depending on the nature of the event the reporting procedures below should be followed. Any questions concerning adverse event reporting should be directed to the Chief Investigator in the first instance.

5.3.1 Non serious AEs

All such events, whether expected or not, should be recorded- it should be specified if only some non-serious AEs will be recorded, any reporting should be consistent with the purpose of the trial end points.

5.3.2 Serious AEs

An SAE form should be completed and emailed to the Chief Investigator within 24 hours. However, relapse and death due to lung cancer, and hospitalisations for elective treatment of a pre-existing condition do not need reporting as SAEs.

All SAEs should be reported to the London Bridge Research Ethics Committee where in the opinion of the Chief Investigator, the event was:

- 'related', ie resulted from the administration of any of the research procedures; and
- 'unexpected', ie an event that is not listed in the protocol as an expected occurrence

Reports of related and unexpected SAEs should be submitted within 15 days of the Chief Investigator becoming aware of the event, using the NRES SAE form for non-IMP studies. The Chief Investigator must also notify the Sponsor of all related and unexpected SAEs.

Local investigators should report any SAEs as required by their Local Research Ethics Committee, Sponsor and/or Research & Development Office.

Contact details for reporting SAEs
RGIT@imperial.ac.uk

For peer review only

BMJ Open

Is personalised prehabilitation feasible to implement for patients undergoing oncological treatment for lung cancer at a London teaching hospital? Protocol of a feasibility trial.

Journal:	*BMJ Open*
Manuscript ID	bmjopen-2023-072367.R3
Article Type:	Protocol
Date Submitted by the Author:	03-Jul-2023

Complete List of Authors:	Wade-Mcbane, Kelly; Imperial College Healthcare NHS Trust<br>King, Alex; Imperial College Healthcare NHS Trust<br>Urch, Catherine; Imperial College Healthcare NHS Trust<br>Johansson, Lina; Imperial College Healthcare NHS Trust<br>Wells, Mary; Imperial College Healthcare NHS Trust

**Primary Subject Heading**:	Oncology
Secondary Subject Heading:	Patient-centred medicine
Keywords:	CHEMOTHERAPY, RADIOTHERAPY, Respiratory tract tumours < ONCOLOGY

SCHOLARONE™
Manuscripts

Is personalised prehabilitation feasible to implement for patients undergoing oncological treatment for lung cancer at a London teaching hospital? Protocol of a feasibility trial.

Kelly Wade-Mcbane[1], Alex King[1], Catherine Urch[1], Lina Johansson[1], Mary Wells[1]

[1]Imperial College Healthcare NHS Trust, London, UK

Correspondence: Kelly Wade-Mcbane, Nutrition and Dietetics Department, Charing Cross Hospital, Imperial College Healthcare NHS Trust, London, UK. Email: k.wade-mcbane@nhs.net

Abstract

Introduction
There is significant potential to improve outcomes for patients with lung cancer in terms of quality of life and survival. There is some evidence that prehabilitation can help, but, to date, this has only been tested in surgical populations, despite 70-80% of lung cancer patients in the UK receiving non-surgical treatment. The physiological and psychological benefits of prehabilitation seen in surgical patients could be extrapolated to those receiving non-surgical treatment, particularly in such a poor prognosis group.
With patients and healthcare professionals, we have co-designed a personalised and evidence-based prehabilitation programme. This draws on a conceptual framework that aligns with patient values and needs as well as functional goals. We aim to investigate whether this programme is feasible to implement and evaluate in clinical practice.

Methods and analysis
An open-label, single-group feasibility study incorporating quantitative assessments, a qualitative free text questionnaire and reflective field notes. Thirty participants will be recruited over an eight-month period from a single London teaching hospital. All recruited participants will receive a personalised prehabilitation programme during their oncological treatment. This includes a one-hour face-to-face appointment prior to, at week three and at week six of their treatment regimen as well as a weekly telephone call. Interventions including nutrition, physical activity and psychological wellbeing are stratified according to a patient's priorities, level of readiness and expressed needs. The primary outcome will be feasibility of the personalised prehabilitation programme in clinical practice by investigating areas of uncertainty regarding patient recruitment, attrition, treatment fidelity, intervention adherence and acceptability of study outcome measures. Secondary outcomes will include quality of life, functional capacity and grip strength.

Ethics and dissemination
Ethical approval has been obtained from the Health Research Authority (HRA) (reference number 22/PR/0390). Results of this study will be disseminated through publication in peer-reviewed articles, presentations at scientific conferences and in collaboration with patient and public involvement representatives.

Keywords: prehabilitation, physical activity, nutrition, wellbeing, chemotherapy, radiotherapy, immunotherapy, conceptual framework, psychological adjustment theory

For peer review only - http://bmjopen.bmj.com/site/about/guidelines.xhtml

Strengths and limitations of this study

- Feasibility study to assess whether a future definitive study can or should be done.
- Inclusive research approach, tailoring interventions to a participant's capabilities.
- Use of a stratified person-centred prehabilitation intervention co-designed with patient partners.
- Risk of potential bias owing to the lead researcher also being the clinician delivering the interventions.

Introduction

Multi-modal prehabilitation programmes incorporating nutrition, physical activity and psychological wellbeing are increasingly being used in surgical cancer pathways. This is in order to optimise health outcomes and enhance recovery by reducing post-operative complications (1–5) and hospital length of stay (4,6–8) as well as improving health reported quality of life (9–12). Few prehabilitation pathways exist for people who do not have surgery, despite the fact that 50-60% of people with cancer in the UK are treated with primary, neo-adjuvant or palliative chemotherapy and/or radiotherapy treatment (13). A small number of studies have investigated the use of prehabilitation in the neo-adjuvant setting and have shown benefit in terms of post-surgical outcomes, (14–18), but only one of these have been in lung cancer (19).

Only a minority of people with lung cancer are eligible for surgery, with 70-80% receiving non-surgical treatment within the UK (20) without any prehabilitation. UK deaths from lung cancer are higher than any other cancer and treatment outcomes are poor (21). There is significant potential to improve outcomes for people with lung cancer and there is some evidence that prehabilitation can help. However, this has only been tested in surgical populations. A small number of studies have shown that prehabilitation before lung cancer surgery is feasible and can improve functional capacity and reduce post-operative complications. However, most of these studies focused on the benefits of exercise prehabilitation as opposed to a multi-modal approach (22–30,19).

Prehabilitation during chemotherapy, radiotherapy or immunotherapy treatment for lung cancer poses significant challenges. Treatment regimes are often prolonged and people may experience a range of toxicities, which could limit their ability to engage in prehabilitation interventions. Personalisation to person-led values and needs is therefore important, as initiation and adherence to any intervention is determined by behavioural, psychological, physiological, environmental and social factors (31–33). This is further supported by Bayly et al's research in thoracic lung cancer (34,35) which found that participants had varied needs, highlighting the importance of tailored support to optimise symptom control, treatment tolerance and independence.

We have co-designed a multi-modal personalised prehabilitation programme with healthcare professionals and patients using a three-step participatory action research approach (36–38). See supplemental material file 1. The multi-modal structure of the programme is informed by the Macmillan, Royal College of Anaesthetists and National Institute of Health Cancer and Nutrition Collaboration, Research Principles and Guidance for Prehabilitation within the Management and Support of People with Cancer (39). This guidance incorporates

For peer review only - http://bmjopen.bmj.com/site/about/guidelines.xhtml

nutrition, physical activity and psychological wellbeing using a tiered approach, tailored to whether patients need universal (applicable to anyone with cancer), targeted (applicable to those at risk of acute, chronic or latent effects of disease or treatment) or specialist (applicable to those with complex needs, severe impairment and/or disability) intervention (39). The first level of intervention – universal – comprises of written information and relevant signposting to support resources for all patients. If psychological, nutrition and/or physical activity needs are identified, patients then receive the appropriate intervention (targeted or specialist) according to their level of need and readiness.

The personalised prehabilitation programme uses the Adversity, Restoration and Compatibility (ARC) framework (32) to underpin a tailored and collaborative approach. The ARC framework provides a synthesised view of how people conceptualise the personal experience of living with and beyond cancer. Consistent with psychological adjustment theory (40), it frames this experience as an ongoing process of learning about the evolving challenge (Adversity), learning how to cope effectively (Restoration) and adapting one's identity (Compatibility), in parallel (see figure 1). Our programme provides a structure and process for personalising prehabilitation, which is person-centred rather than providing a purely logistical or psychometric approach. As recommend by Hickmann et al (41), such an approach could be key to empower patients to maintain progress through the prolonged, more variable context of peri-oncological prehabilitation.

This proposed feasibility study aims to investigate whether a personalised prehabilitation programme co-designed with healthcare professionals and patients and informed by theory and evidence is feasible to implement and evaluate in clinical practice and could thereby support a future definitive study.

Methods and analysis

Feasibility studies are particularly relevant to assess whether a future definitive study can or should be done (42–44). Using the Orsmond & Cohn framework for feasibility studies (45), this open-label, single-group study will use a mix of methods to understand the different aspects of feasibility. This will be through the inclusion of quantitative assessments, a qualitative free text questionnaire and reflective field notes to investigate areas of uncertainty regarding patient recruitment, attrition, treatment fidelity, intervention adherence and acceptability of study outcome measures. This is situated within the feasibility stage of the Medical Research Council (MRC) framework for developing and evaluating complex interventions (46). It will help inform if the personalised prehabilitation programme can be implemented and evaluated in clinical practice and will provide data to estimate the parameters required to design a future definitive study. The Consolidated Standards of Reporting Trials (CONSORT) reporting guideline for pilot and feasibility studies (42) has been used for this protocol. See supplemental material file 2.

Eligibility criteria

Inclusion criteria: Adults (≥18 years) with a diagnosis of lung cancer who are due to commence chemotherapy, radiotherapy or immunotherapy treatment. This study includes all lung cancer types and stages. People with co-morbidities and those on medication will be considered eligible. Interventions will be tailored to a participant's capabilities, thereby promoting inclusivity and optimisation of participant recruitment.

For peer review only - http://bmjopen.bmj.com/site/about/guidelines.xhtml

Exclusion criteria: Participants who are due to undergo lung cancer surgery, have had previous lung cancer surgery or unable to give informed consent will not be eligible for this study.

Recruitment

Participants will be recruited from the lung cancer clinic at a single London teaching hospital via referral from the Lung Cancer Multi-Disciplinary Team (LCMDT). Recruitment will continue until 30 participants have been recruited or until eight months have elapsed. A pragmatic sample size of 30 participants over an eight-month recruitment period from November 2022 – June 2023 has been selected, which is feasible within the time frame of this study. This is based on 144 - 240 new lung cancer diagnoses per year at the study hospital, with an assumption of a 50% uptake over an eight-month period. Furthermore, it is in accordance with published guidance on recommended sample sizes for feasibility studies (47,48).

The personalised prehabilitation programme

All participants will be required to attend three face-to-face appointments at the study hospital for study assessments, but the interventions will be delivered using a hybrid approach; either face-to-face at the study hospital or remotely via telephone or video conferencing depending on participant preference.

The three face-to-face sessions with the lead researcher will be prior to, at week three and at week six of a participant's oncological treatment regimen.
Following consent, participants will be guided through a set of questionnaires (as detailed below) to complete prior to their first face-to-face appointment. These questionnaires will be reviewed during the initial session, enabling the nutrition, physical activity and/or psychological wellbeing interventions to be stratified according to a participant's priorities, level of readiness and expressed needs:

- Cancer Behaviour Inventory (CBI) brief form - to measure self-efficacy for coping with cancer. A score of less than 50% indicates a low level of readiness, whereas a score of 50% or greater indicates a high level of readiness (49).
- Patient Activation Measure (PAM) - to measure a person's knowledge, skills and confidence in managing their own health and wellbeing. A score of one-two indicates a low level of readiness, whereas a score of three-four indicates a high level of readiness (50).
- Modified Godin leisure time exercise questionnaire - to measure activity. A score of 14 or greater demonstrates that a person is active, thereby indicating a low level of need for physical activity. Conversely, a score less than 14 demonstrates that a person is insufficiently active, thereby indicating a high level of need to optimise activity levels (51).
- Patient Generated Subjective Global Assessment (PG-SGA) short form - to identify malnutrition risk. A score less than four demonstrates no or minimal risk for malnutrition, thereby indicating a low level of need for nutritional support. Conversely, a score of four or greater demonstrates that a person is at risk of malnutrition or is malnourished, thereby indicating a high level of need for nutrition support (52).
- Emotions Thermometer (ET) - to evaluate levels of emotional distress. A score in any domain less than five or a score of less than four in need for help demonstrates low

For peer review only - http://bmjopen.bmj.com/site/about/guidelines.xhtml

levels of emotional distress, thereby indicating a low level of need. Conversely, a score of five or greater in any domain or a score of four or greater in need for help demonstrates high levels of emotional distress, thereby indicating a high level of need (53).

- Patient Generated Index (PGI) - to identify participant priorities (54).

Participant needs will also be determined objectively using the six-minute walk test (6MWT) to assess functional capacity and handgrip strength to assess upper body strength. Participants will undertake these functional assessments during the face-to-face sessions.

Questionnaire responses and outcomes from the functional measurements will inform the conversation with the participant about their priorities and enable interventions to be stratified according to a participant's level of readiness and need. If a subjective (questionnaire) or objective measurement (6MWT or handgrip strength) indicates a low or high level of readiness or need, the intervention will be tailored to the individual accordingly. See figures 2a, 2b and 2c.

The personalised prehabilitation programme uses a universal, targeted and specialist approach to prehabilitation interventions, in line with current guidance (39). All participants will receive a universal intervention comprising of written information and relevant signposting to cancer specific nutrition, physical activity and/or psychosocial wellbeing support resources. If a participant identifies a psychological, nutrition and/or physical activity need, they will then receive the appropriate intervention according to their level of need and readiness (see figures 2a, 2b and 2c). Targeted interventions will be delivered via the use of certified apps and/or specific group activities at Maggie's cancer centre (an independent charity that provides free cancer support, workshops and courses to help anyone affected by cancer). For example, exercise classes or nutritional educational sessions for participants undergoing treatment as well as the opportunity to attend a lung cancer support group. These group activities are run by cancer support workers or qualified instructors. Specialist interventions involve a 1:1 assessment with a registered healthcare professional such as a physiotherapist, dietitian and/or a clinical psychologist depending on a participant's needs. The objectives of specialist intervention with the physiotherapist and dietitian are to increase physical activity and optimise dietary intake to minimise loss of body weight and muscle mass, respectively. Specialist intervention with the physiotherapist will include tailored advice regarding resistance and aerobic exercise. In comparison, specialist intervention with the dietitian will include a full individualised nutritional assessment, taking into account anthropometric, biochemical and clinical parameters as well as undertaking a comprehensive diet history and calculation of nutritional requirements to minimise further malnutrition.

Owing to the prolonged nature of chemotherapy, radiotherapy or immunotherapy treatment in contrast to a defined one-off target such as surgery, person-led values and needs are likely to be more variable within the context of peri-oncological prehabilitation. Throughout treatment, participants will be asked to keep a daily diary to record their symptoms, appetite, mobility and level of distress each day using a Likert scale. This will provide participants with a visual representation of their progress and help identify any adverse trends.
Participants will also receive a weekly telephone call from the lead researcher (a specialist oncology dietitian). Participants will be able to use the diary to highlight any days they found

For peer review only - http://bmjopen.bmj.com/site/about/guidelines.xhtml

particularly good or difficult and have the opportunity to discuss these. Personalisation is central to this study and the language used during the face-to-face appointments and the weekly telephone calls has validation and compassion at its core to help build self-efficacy and sustain behaviour change (55). The use of the PGI to understand personal priorities is fundamental to this personalised approach. A systematic review (56) demonstrated the large potential value of using the PGI by enabling people with cancer to participate in the decision-making process. This is key to empower people to take ownership of their own health and for the sustainability of long-term positive behaviour change and self-care.

A traffic light system with varying levels of acceptability has often been cited as a means of judging feasibility (42,44,57–60). Following consensus amongst the research team, a priori feasibility endpoints were defined using a RAG criteria as shown in table 1.

Table 1. A priori feasibility endpoints using a RAG criteria.

**Parameter**	**Red (don't proceed to a further study)**	**Amber (modifications +/- further study required)**	**Green (proceed to a larger study)**
Recruitment rate	<30%	30-60%	>60%
Refusal rate	>50%	30-50%	<30%
Attrition rate	>80%	20-80%	<20%
Intervention adherence	<30%	30-60%	>60%

Primary objective and outcome measure

The primary objective is to assess the feasibility of the personalised prehabilitation programme in clinical practice by gathering data on how accessible and relevant it is for lung cancer patients undergoing chemotherapy, radiotherapy or immunotherapy treatment. This will be addressed using the research objectives and methods of assessment outlined in table 2 below.

Table 2. Research objectives and methods of assessment to investigate the feasibility of the personalised prehabilitation programme in clinical practice.

**Research objective**	**Method of assessment(s)**
To estimate eligibility, recruitment, refusal and attrition rates (including the reasons why)	Descriptive statistics – number approached, eligible, recruited, refused and number who drop out and at what stage<br>Field notes taken during the Lung Cancer Multi-Disciplinary Team (LCMDT)<br>Participant discussion
To assess adherence to the personalised prehabilitation interventions	Weekly telephone call – adherence to goals set
To assess the barriers and facilitators to adherence and intervention fidelity	Daily diary – symptom log<br>Weekly telephone call<br>Short free text questionnaire at the end of the study, analysed thematically
To evaluate acceptability of the study processes and outcome measures	Completion rates<br>Weekly telephone call<br>Short free text questionnaire at the end of the study, analysed thematically

For peer review only - http://bmjopen.bmj.com/site/about/guidelines.xhtml

To determine the 'prehabilitation window' (length of time between diagnosis and the start of treatment)	Descriptive statistics – date of diagnosis, date of start of treatment, date of start and finish of prehabilitation
To determine the extent to which the intervention/s are likely to have an impact on psychological wellbeing, nutrition and/or physical activity and quality of life	Questionnaires – change between baseline and follow up<br>Daily diary – symptom log<br>Weekly telephone call<br>Short free text questionnaire at the end of the study, analysed thematically]<br>Functional objective assessments
To determine the number of participants who require universal, targeted and specialist intervention	Questionnaires – level of need and readiness<br>Descriptive statistics

Heterogeneity of participant characteristics is expected within the study. This will provide valuable insights into how different people experience the personalised prehabilitation programme.

Secondary objectives and outcome measures

Functional benefits of prehabilitation can be seen in as little as two weeks (39,61). The secondary objectives will assess the impact of the personalised prehabilitation programme on:

- Overall quality of life as measured by:
  - European Organisation for Research and Treatment of Cancer (EORTC) QLQ-C30; a cancer specific assessment of quality of life, sensitive to changes over time (62)
  - EuroQOL group EQ-5D-5L for a descriptive overview of quality of life, which can be used for economic evaluation (63).
- Functional capacity as measured by the six-minute walk test (6MWT) (64). The 6MWT is a safe, valid and reliable measure of functional capacity in cancer populations (65). All participants will perform a standardised, self-paced 6MWT in a 30-metre long corridor. Each participant will be instructed to walk as far as they can for six minutes. Participants will be allowed to stop at any time. The distance covered (to the nearest metre) after the six minutes will be measured. A cut off value of 400m has been used to determine a participant's level of need for physical activity prehabilitation intervention. This is in line with a study by Kasymjanova et al (66) on the prognostic value of the 6MWT in advanced non-small cell lung cancer in patients undergoing chemotherapy.
- Handgrip strength as measured by a handgrip dynamometer (67). Grip strength has shown to be correlated with overall health status (68–72). Each participant will be instructed to sit in a chair and bend the elbow of their non-dominant arm at a 90-degree angle. The lead researcher will ask the participant to squeeze the device as hard as they can. Each participant will repeat this three times and the average will be recorded. A cut off value of 27kg for men and 16kg for women has been used to determine a participant's level of need for nutritional prehabilitation intervention. This is based on the revised European consensus guidelines on the definition and diagnosis of sarcopenia (73).

To tailor the intervention, the above outcome measures will be assessed prior to starting treatment and before the personalised prehabilitation programme begins (baseline), at week three and at week six of a participant's oncological treatment (end point).

For peer review only - http://bmjopen.bmj.com/site/about/guidelines.xhtml

Demographic and clinical data including age, gender, level of education achieved, ethnicity, employment status, level of social support (measured using the Duke-University of North Carolina Functional Social Support Questionnaire; DUFSSQ) (74), diagnosis, staging, treatment and co-morbidities (measured using the Adult Co-morbidity Evaluation (ACE) 27 score) (75) will be collected at baseline.

Training

The lead researcher is a highly specialised oncology dietitian trained in advanced communication and psychological skills (76) to manage complex conversations with patients and help recognise and respond to psychological distress. The lead researcher is familiar with using a handgrip dynamometer from previous experience in clinical practice. Training in conducting the 6MWT has been provided by a specialist oncology physiotherapist.

Patient and public involvement (PPI)

Four patients (and a spouse) who had recently completed chemotherapy or radiotherapy treatment for lung cancer were consulted on the personalised prehabilitation programme via a focus group. This was separate to focus groups used in the initial co-design process to develop the personalised prehabilitation programme, which used a three-step participatory action research approach (36–38). See supplemental material file 1. Members of the focus group were very positive about the programme, with many patients reporting that they found having a strong mind-set, staying active and eating well helped them get through treatment. These are key components of the personalised prehabilitation programme. The focus group also reviewed the study questionnaires and patient documentation. Patients and their relatives expressed the benefits of having a weekly telephone call to discuss their concerns and reported that the questionnaires and daily diary would make them feel supported, as the information would be tailored to them individually.

All participants who participate in the study will be offered the opportunity to provide feedback on their experience of the personalised prehabilitation programme via the use of a short free text questionnaire. This feedback will help inform future programme adjustments and have the potential to improve patient experience.

Data collection and management

Data will be collected and stored according to the Data Protection Act (2018) and in line with General Data Protection Regulation (GDPR).

All data included in this study will be kept as part of the participants clinical care record on a secure password protected electronic patient record system at the study hospital.

A subset of this data will be extracted and entered into a case report form, which will be kept on a secure password protected database at the study hospital and only accessed by the research team. This data will be pseudonymised and all participants will be given a unique study number.

Statistical analysis plan

This study includes a small cohort of lung cancer participants from a single London teaching hospital. There are no groups to detect differences between, therefore, no formal tests of statistical significance will be undertaken.

Descriptive statistics will be used to analyse baseline demographic and clinical data.

Continuous measures will be reported as means, standard deviations and/or medians along

For peer review only - http://bmjopen.bmj.com/site/about/guidelines.xhtml

with confidence intervals, while categorical data will be reported as percentages. Missing data will be reported where applicable.
Inductive thematic analysis will be used to analyse qualitative data from the short free text questionnaire regarding patient experience.
Feasibility will be determined if the a priori thresholds for feasibility have been met.
The data collected will be used to inform the design of a larger randomised pilot study.

Strengths and limitations
This study uses a novel way of delivering a multi-modal prehabilitation programme through the use of a personalised, inclusive research approach that aligns with patient values, needs and functional goals, with interventions tailored to a participant's capabilities. The personalised prehabilitation programme provides a structure and process for personalising prehabilitation that is person-centred and underpinned by a conceptual framework, which is consistent with psychological adjustment theory. This could be key to empower patients to maintain activity through the longer, more variable context of peri-oncological prehabilitation.
This study will help address key uncertainties, which need to be resolved before implementation, including engagement and adherence to prehabilitation interventions in lung cancer patients receiving chemotherapy, radiotherapy or immunotherapy treatment.
This study is limited by (a) lack of a comparison group (b) the small sample size used (c) self-reported adherence to the prehabilitation interventions and (d) the lead researcher also being the clinician delivering the interventions and performing assessments. In order to mitigate potential bias regarding the latter, where possible, aspects of the programme have been pre-defined.

Ethics and dissemination
Ethical approval was obtained from the Health Research Authority (HRA) on 13th April 2022 (reference number 22/PR/0390) prior to the start of the study. Any modifications will be approved by the HRA. This study has been registered with ClinicalTrials.gov (NCT: NCT05318807). Imperial College Healthcare NHS Trust is the sponsor of the study.
Monitoring and auditing will be conducted in accordance with the sponsor's policies and procedures. Results of this study will be disseminated to academics, stakeholders, cancer alliances, relevant charities and to the public and people with cancer. This will be through publication in peer-reviewed articles, presentations at scientific conferences, social media and collaboration with patient and public involvement representatives.

Author contributions

KWM is the principal investigator. KWM devised the clinical question, applied for funding and research ethics and was involved in the conception and design of the study and protocol. AK contributed to the study design and methods. AK and CU were involved in the conception and design of the study. LJ and MW contributed to all versions of the protocol. AK, CU and MW provided their expertise in cancer care and contributed to the knowledge needed for prehabilitation. CU, LJ and MW provided supervision and mentorship. All authors read and approved the final protocol.

For peer review only - http://bmjopen.bmj.com/site/about/guidelines.xhtml

Funding

This work was supported by Health Education England and the National Institute for Health and Care Research as part of an ICA Pre-doctoral Clinical and Practitioner Academic Fellowship (Grant award number: NIHR302680).

Competing interests

The authors declare that they have no competing interests.

References

1. Berkel AEM, Bongers BC, van Kamp MJS, Kotte H, Weltevreden P, de Jongh FHC, et al. The effects of prehabilitation versus usual care to reduce postoperative complications in high-risk patients with colorectal cancer or dysplasia scheduled for elective colorectal resection: study protocol of a randomized controlled trial. BMC Gastroenterol. 2018 Dec;18(1):29.

2. van Rooijen S, Carli F, Dalton S, Thomas G, Bojesen R, Le Guen M, et al. Multimodal prehabilitation in colorectal cancer patients to improve functional capacity and reduce postoperative complications: the first international randomized controlled trial for multimodal prehabilitation. BMC Cancer. 2019 Dec;19(1):98.

3. Moore J, Merchant Z, Rowlinson K, McEwan K, Evison M, Faulkner G, et al. Implementing a system-wide cancer prehabilitation programme: The journey of Greater Manchester's 'Prehab4cancer.' Eur J Surg Oncol. 2021 Mar;47(3):524–32.

4. Perry R, Herbert G, Atkinson C, England C, Northstone K, Baos S, et al. Pre-admission interventions (prehabilitation) to improve outcome after major elective surgery: a systematic review and meta-analysis. BMJ Open. 2021 Sep;11(9):e050806.

5. Molenaar CJ, van Rooijen SJ, Fokkenrood HJ, Roumen RM, Janssen L, Slooter GD. Prehabilitation versus no prehabilitation to improve functional capacity, reduce postoperative complications and improve quality of life in colorectal cancer surgery. Cochrane Colorectal Group, editor. Cochrane Database Syst Rev [Internet]. 2022 May 19 [cited 2023 Jan 26];2022(5). Available from: http://doi.wiley.com/10.1002/14651858.CD013259.pub2

6. Gillis C, Buhler K, Bresee L, Carli F, Gramlich L, Culos-Reed N, et al. Effects of Nutritional Prehabilitation, With and Without Exercise, on Outcomes of Patients Who Undergo Colorectal Surgery: A Systematic Review and Meta-analysis. Gastroenterology. 2018 Aug;155(2):391-410.e4.

7. Lambert JE, Hayes LD, Keegan TJ, Subar DA, Gaffney CJ. The Impact of Prehabilitation on Patient Outcomes in Hepatobiliary, Colorectal, and Upper Gastrointestinal Cancer Surgery: A PRISMA-Accordant Meta-analysis. Ann Surg. 2021 Jul;274(1):70–7.

8. Waterland JL, McCourt O, Edbrooke L, Granger CL, Ismail H, Riedel B, et al. Efficacy of Prehabilitation Including Exercise on Postoperative Outcomes Following Abdominal Cancer Surgery: A Systematic Review and Meta-Analysis. Front Surg. 2021 Mar 19;8:628848.

For peer review only - http://bmjopen.bmj.com/site/about/guidelines.xhtml

9. Treanor C, Kyaw T, Donnelly M. An international review and meta-analysis of prehabilitation compared to usual care for cancer patients. J Cancer Surviv. 2018 Feb;12(1):64–73.

10. Fulop A, Lakatos L, Susztak N, Szijarto A, Banky B. The effect of trimodal prehabilitation on the physical and psychological health of patients undergoing colorectal surgery: a randomised clinical trial. Anaesthesia. 2021 Jan;76(1):82–90.

11. Wu F, Rotimi O, Laza-Cagigas R, Rampal T. The Feasibility and Effects of a Telehealth-Delivered Home-Based Prehabilitation Program for Cancer Patients during the Pandemic. Curr Oncol. 2021 Jun 17;28(3):2248–59.

12. Allen SK, Brown V, White D, King D, Hunt J, Wainwright J, et al. Multimodal Prehabilitation During Neoadjuvant Therapy Prior to Esophagogastric Cancer Resection: Effect on Cardiopulmonary Exercise Test Performance, Muscle Mass and Quality of Life—A Pilot Randomized Clinical Trial. Ann Surg Oncol. 2022 Mar;29(3):1839–50.

13. Cancer Research UK. Cancer Research UK. Cancer treatment statistics 2013-2016 [Internet]. 2013 2016 [cited 2022 Oct 3]. Available from: https://www.cancerresearchuk.org/health-professional/cancer-statistics/treatment

14. West MA, Loughney L, Lythgoe D, Barben CP, Sripadam R, Kemp GJ, et al. Effect of prehabilitation on objectively measured physical fitness after neoadjuvant treatment in preoperative rectal cancer patients: a blinded interventional pilot study. Br J Anaesth. 2015 Feb;114(2):244–51.

15. Christensen JF, Simonsen C, Banck-Petersen A, Thorsen-Streit S, Herrstedt A, Djurhuus SS, et al. Safety and feasibility of preoperative exercise training during neoadjuvant treatment before surgery for adenocarcinoma of the gastro-oesophageal junction: Preoperative exercise training during neoadjuvant treatment for gastro-oesophageal junction adenocarcinoma. BJS Open. 2019 Feb;3(1):74–84.

16. Dewberry LC, Wingrove LJ, Marsh MD, Glode AE, Schefter TE, Leong S, et al. Pilot Prehabilitation Program for Patients With Esophageal Cancer During Neoadjuvant Therapy and Surgery. J Surg Res. 2019 Mar;235:66–72.

17. Halliday LJ, Doganay E, Wynter-Blyth V, Osborn H, Buckley J, Moorthy K. Adherence to Pre-operative Exercise and the Response to Prehabilitation in Oesophageal Cancer Patients. J Gastrointest Surg. 2021 Apr;25(4):890–9.

18. Ligthart-Melis GC, Weijs PJM, te Boveldt ND, Buskermolen S, Earthman CP, Verheul HMW, et al. Dietician-delivered intensive nutritional support is associated with a decrease in severe postoperative complications after surgery in patients with esophageal cancer: INS limits postoperative complications. Dis Esophagus. 2013 Aug;26(6):587–93.

19. Schmid S, Minnella EM, Pilon Y, Rokah M, Rayes R, Najmeh S, et al. Neoadjuvant Prehabilitation Therapy for Locally Advanced Non–Small-Cell Lung Cancer: Optimizing Outcomes Throughout the Trajectory of Care. Clin Lung Cancer. 2022 Nov;23(7):593–9.

20. Cancer Research UK. Cancer Research UK. Lung cancer treatment statistics 2013-2014 [Internet]. 2013 2014 [cited 2022 Oct 17]. Available from: https://www.cancerresearchuk.org/health-professional/cancer-statistics/statistics-by-cancer-type/lung-cancer/diagnosis-and-treatment#heading-Three

For peer review only - http://bmjopen.bmj.com/site/about/guidelines.xhtml

21. Cancer Research UK. Cancer Research UK. Lung cancer mortality statistics 2017-2019 [Internet]. 2017 2019 [cited 2022 Oct 17]. Available from: https://www.cancerresearchuk.org/health-professional/cancer-statistics/statistics-by-cancer-type/lung-cancer#heading-Five

22. Laurent H, Aubreton S, Galvaing G, Pereira B, Merle P, Richard R, et al. Preoperative respiratory muscle endurance training improves ventilatory capacity and prevents pulmonary postoperative complications after lung surgery. Eur J Phys Rehabil Med [Internet]. 2020 Feb [cited 2022 Oct 17];56(1). Available from: https://www.minervamedica.it/index2.php?show=R33Y2020N01A0073

23. Liu Z, Qiu T, Pei L, Zhang Y, Xu L, Cui Y, et al. Two-Week Multimodal Prehabilitation Program Improves Perioperative Functional Capability in Patients Undergoing Thoracoscopic Lobectomy for Lung Cancer: A Randomized Controlled Trial. Anesth Analg. 2020 Sep;131(3):840–9.

24. Ricketts WM, Bollard K, Streets E, Hutton K, Hornby C, Lau K. Feasibility of setting up a pre-operative optimisation 'pre-hab' service for lung cancer surgery in the UK. Perioper Med. 2020 Dec;9(1):14.

25. Avancini A, Cavallo A, Trestini I, Tregnago D, Belluomini L, Crisafulli E, et al. Exercise prehabilitation in lung cancer: Getting stronger to recover faster. Eur J Surg Oncol. 2021 Aug;47(8):1847–55.

26. Bradley P, Merchant Z, Rowlinson-Groves K, Grundy S, Al-Najjar H, Brown L, et al. P212 Prehab4Cancer: an innovative regional lung cancer prehabilitation service. In: The lung cancer diagnostic journey [Internet]. BMJ Publishing Group Ltd and British Thoracic Society; 2021 [cited 2022 Oct 17]. p. A204.2-A205. Available from: https://thorax.bmj.com/lookup/doi/10.1136/thorax-2020-BTSabstracts.357

27. Ferreira V, Minnella EM, Awasthi R, Gamsa A, Ferri L, Mulder D, et al. Multimodal Prehabilitation for Lung Cancer Surgery: A Randomized Controlled Trial. Ann Thorac Surg. 2021 Nov;112(5):1600–8.

28. Goldsmith I, Chesterfield-Thomas G, Toghill H. Pre-treatment optimization with pulmonary rehabilitation in lung cancer: Making the inoperable patients operable. EClinicalMedicine. 2021 Jan;31:100663.

29. Bradley P, Merchant Z, Rowlinson-Groves K, Taylor M, Moore J, Evison M. Feasibility and outcomes of a real-world regional lung cancer prehabilitation programme in the UK. Br J Anaesth. 2022 Jul;S0007091222002951.

30. Granger C, Cavalheri V. Preoperative exercise training for people with non-small cell lung cancer. Cochrane Lung Cancer Group, editor. Cochrane Database Syst Rev [Internet]. 2022 Sep 28 [cited 2023 Jan 11];2022(9). Available from: http://doi.wiley.com/10.1002/14651858.CD012020.pub3

31. Ferreira V, Agnihotram RV, Bergdahl A, van Rooijen SJ, Awasthi R, Carli F, et al. Maximizing patient adherence to prehabilitation: what do the patients say? Support Care Cancer. 2018 Aug;26(8):2717–23.

32. Boutillier CL, Archer S, Barry C, King A, Mansfield L, Urch C. Conceptual framework for living with and beyond cancer: A systematic review and narrative synthesis. Psychooncology. 2019;28(5):948–59.

For peer review only - http://bmjopen.bmj.com/site/about/guidelines.xhtml

33. Grimmett C, Bradbury K, Dalton SO, Fecher-Jones I, Hoedjes M, Varkonyi-Sepp J, et al. The Role of Behavioral Science in Personalized Multimodal Prehabilitation in Cancer. Front Psychol. 2021;12:261.

34. Bayly J, Edwards BM, Peat N, Warwick G, Hennig IM, Arora A, et al. Developing an integrated rehabilitation model for thoracic cancer services: views of patients, informal carers and clinicians. Pilot Feasibility Stud. 2018 Dec;4(1):160.

35. Bayly J, Fettes L, Douglas E, Teixiera MJ, Peat N, Tunnard I, et al. Short-term integrated rehabilitation for people with newly diagnosed thoracic cancer: a multi-centre randomized controlled feasibility trial. Clin Rehabil. 2020 Feb;34(2):205–19.

36. Bate P, Robert G. Experience-based design: from redesigning the system around the patient to co-designing services with the patient. Qual Saf Health Care. 2006 Oct 1;15(5):307–10.

37. Tsianakas V, Robert G, Richardson A, Verity R, Oakley C, Murrells T, et al. Enhancing the experience of carers in the chemotherapy outpatient setting: an exploratory randomised controlled trial to test impact, acceptability and feasibility of a complex intervention co-designed by carers and staff. Support Care Cancer. 2015 Oct;23(10):3069–80.

38. Cornish F, Breton N, Moreno-Tabarez U, Delgado J, Rua M, de-Graft Aikins A, et al. Participatory action research. Nat Rev Methods Primer. 2023 Apr 27;3(1):34.

39. Macmillan Cancer Support, NIHR Cancer and Nutrition Collaboration, Royal College of Anaesthetists. Principles and guidance for prehabilitation within the management and support of people with cancer [Internet]. Macmillan Cancer Support; 2019 [cited 2021 Oct 27]. Available from: https://www.macmillan.org.uk/healthcare-professionals/news-and-resources/guides/principles-and-guidance-for-prehabilitation

40. Brennan J. Adjustment to cancer?coping or personal transition? Psychooncology. 2001 Jan;10(1):1–18.

41. Hickmann E, Richter P, Schlieter H. All together now – patient engagement, patient empowerment, and associated terms in personal healthcare. BMC Health Serv Res. 2022 Sep 2;22(1):1116.

42. Eldridge SM, Chan CL, Campbell MJ, Bond CM, Hopewell S, Thabane L, et al. CONSORT 2010 statement: extension to randomised pilot and feasibility trials. BMJ. 2016 Oct 24;i5239.

43. Eldridge SM, Lancaster GA, Campbell MJ, Thabane L, Hopewell S, Coleman CL, et al. Defining Feasibility and Pilot Studies in Preparation for Randomised Controlled Trials: Development of a Conceptual Framework. Lazzeri C, editor. PLOS ONE. 2016 Mar 15;11(3):e0150205.

44. Pearson N, Naylor PJ, Ashe MC, Fernandez M, Yoong SL, Wolfenden L. Guidance for conducting feasibility and pilot studies for implementation trials. Pilot Feasibility Stud. 2020 Dec;6(1):167.

45. Orsmond GI, Cohn ES. The Distinctive Features of a Feasibility Study: Objectives and Guiding Questions. OTJR Occup Particip Health. 2015 Jul;35(3):169–77.

For peer review only

For peer review only - http://bmjopen.bmj.com/site/about/guidelines.xhtml

46. Skivington K, Matthews L, Simpson SA, Craig P, Baird J, Blazeby JM, et al. A new framework for developing and evaluating complex interventions: update of Medical Research Council guidance. BMJ. 2021 Sep 30;n2061.

47. Julious SA. Sample size of 12 per group rule of thumb for a pilot study. Pharm Stat. 2005 Oct;4(4):287–91.

48. Sim J, Lewis M. The size of a pilot study for a clinical trial should be calculated in relation to considerations of precision and efficiency. J Clin Epidemiol. 2012 Mar;65(3):301–8.

49. Heitzmann CA, Merluzzi TV, Jean-Pierre P, Roscoe JA, Kirsh KL, Passik SD. Assessing self-efficacy for coping with cancer: development and psychometric analysis of the brief version of the Cancer Behavior Inventory (CBI-B). Psychooncology. 2011 Mar;20(3):302–12.

50. Hibbard JH, Mahoney ER, Stockard J, Tusler M. Development and Testing of a Short Form of the Patient Activation Measure. Health Serv Res. 2005 Dec;40(6p1):1918–30.

51. Godin G. The Godin-Shephard Leisure-Time Physical Activity Questionnaire. Health Fit J Can. 2011 Jan 30;18-22 Pages.

52. Ottery F. Definition of standardized nutritional assessment and interventional pathways in oncology. Nutrition. 1996 Jan;12:S15–9.

53. Harju E, Michel G, Roser K. A systematic review on the use of the emotion thermometer in individuals diagnosed with cancer. Psychooncology. 2019 Sep;28(9):1803–18.

54. Ruta DA, Garratt AM, Leng M, Russell IT, MacDonald LM. A New Approach to the Measurement of Quality of Life: The Patient-Generated Index. Med Care. 1994 Nov;32(11):1109–26.

55. Kwasnicka D, Dombrowski SU, White M, Sniehotta F. Theoretical explanations for maintenance of behaviour change: a systematic review of behaviour theories. Health Psychol Rev. 2016 Jul 2;10(3):277–96.

56. Tang JA, Oh T, Scheer JK, Parsa AT. The current trend of administering a patient-generated index in the oncological setting: a systematic review. Oncol Rev [Internet]. 2014 Jun 30 [cited 2022 Oct 17]; Available from: https://www.frontierspartnerships.org/articles/10.4081/oncol.2014.245

57. Thabane L, Ma J, Chu R, Cheng J, Ismaila A, Rios LP, et al. A tutorial on pilot studies: the what, why and how. BMC Med Res Methodol. 2010 Jan 6;10(1):1.

58. Avery KNL, Williamson PR, Gamble C, O'Connell Francischetto E, Metcalfe C, Davidson P, et al. Informing efficient randomised controlled trials: exploration of challenges in developing progression criteria for internal pilot studies. BMJ Open. 2017 Feb;7(2):e013537.

59. Hallingberg B, Turley R, Segrott J, Wight D, Craig P, Moore L, et al. Exploratory studies to decide whether and how to proceed with full-scale evaluations of public health interventions: a systematic review of guidance. Pilot Feasibility Stud. 2018 Dec;4(1):104.

60. Mellor K, Albury C, Dutton SJ, Eldridge S, Hopewell S. Recommendations for progression criteria during external randomised pilot trial design, conduct, analysis and reporting. Pilot Feasibility Stud. 2023 Apr 15;9(1):59, s40814-023-01291–5.

For peer review only - http://bmjopen.bmj.com/site/about/guidelines.xhtml

For peer review only

61. Faithfull S, Turner L, Poole K, Joy M, Manders R, Weprin J, et al. Prehabilitation for adults diagnosed with cancer: A systematic review of long-term physical function, nutrition and patient-reported outcomes. Eur J Cancer Care (Engl) [Internet]. 2019 Jul [cited 2021 Oct 27];28(4). Available from: https://onlinelibrary.wiley.com/doi/10.1111/ecc.13023

62. Aaronson NK, Ahmedzai S, Bergman B, Bullinger M, Cull A, Duez NJ, et al. The European Organization for Research and Treatment of Cancer QLQ-C30: A Quality-of-Life Instrument for Use in International Clinical Trials in Oncology. JNCI J Natl Cancer Inst. 1993 Mar 3;85(5):365–76.

63. Herdman M, Gudex C, Lloyd A, Janssen Mf, Kind P, Parkin D, et al. Development and preliminary testing of the new five-level version of EQ-5D (EQ-5D-5L). Qual Life Res. 2011 Dec;20(10):1727–36.

64. ATS Statement: Guidelines for the Six-Minute Walk Test. Am J Respir Crit Care Med. 2002 Jul 1;166(1):111–7.

65. Schmidt K, Vogt L, Thiel C, Jäger E, Banzer W. Validity of the Six-Minute Walk Test in Cancer Patients. Int J Sports Med. 2013 Feb 26;34(07):631–6.

66. Kasymjanova G, Correa JA, Kreisman H, Dajczman E, Pepe C, Dobson S, et al. Prognostic Value of the Six-Minute Walk in Advanced Non-small Cell Lung Cancer. J Thorac Oncol. 2009 May;4(5):602–7.

67. Spruit MA, Sillen MJH, Groenen MTJ, Wouters EFM, Franssen FME. New Normative Values for Handgrip Strength: Results From the UK Biobank. J Am Med Dir Assoc. 2013 Oct;14(10):775.e5-775.e11.

68. Musalek C, Kirchengast S. Grip Strength as an Indicator of Health-Related Quality of Life in Old Age—A Pilot Study. Int J Environ Res Public Health. 2017 Nov 24;14(12):1447.

69. Celis-Morales C, Welsh P, Lyall D, Steell L, Petermann F, Anderson J, et al. Associations of grip strength with cardiovascular, respiratory, and cancer outcomes and all cause mortality: prospective cohort study of half a million UK Biobank participants. 2018 Aug 5;361:1–10.

70. Bohannon RW. Grip Strength: An Indispensable Biomarker For Older Adults. Clin Interv Aging. 2019 Oct;Volume 14:1681–91.

71. Soysal P, Hurst C, Demurtas J, Firth J, Howden R, Yang L, et al. Handgrip strength and health outcomes: Umbrella review of systematic reviews with meta-analyses of observational studies. J Sport Health Sci. 2021 May;10(3):290–5.

72. Jiang R, Westwater ML, Noble S, Rosenblatt M, Dai W, Qi S, et al. Associations between grip strength, brain structure, and mental health in > 40,000 participants from the UK Biobank. BMC Med. 2022 Sep 9;20(1):286.

73. Cruz-Jentoft AJ, Bahat G, Bauer J, Boirie Y, Bruyère O, Cederholm T, et al. Sarcopenia: revised European consensus on definition and diagnosis. Age Ageing. 2019 Jan 1;48(1):16–31.

For peer review only - http://bmjopen.bmj.com/site/about/guidelines.xhtml

74. Saracino R, Kolva E, Rosenfeld B, Breitbart W. Measuring social support in patients with advanced medical illnesses: An analysis of the Duke–UNC Functional Social Support Questionnaire. Palliat Support Care. 2015 Oct;13(5):1153–63.

75. Kallogjeri D, Piccirillo JF, Spitznagel EL, Steyerberg EW. Comparison of scoring methods for ACE-27: Simpler is better. J Geriatr Oncol. 2012 Jul;3(3):238–45.

76. London Cancer Alliance. Guidance for Level Two Healthcare Workers and Level Three/Four Supervisors [Internet]. London Cancer Alliance; 2015. Available from: https://rmpartners.nhs.uk/wp-content/uploads/2017/03/lca-guidance-for-level-2-healthcare-workers-and-level-3_4-supervisors-september-2015.pdf

For peer review only

For peer review only - http://bmjopen.bmj.com/site/about/guidelines.xhtml

Figure legends

Figure 1. Diagrammatic representation of the Adversity, Restoration and Compatibility (ARC) model of the experience of adjusting to living with and beyond cancer. The waves and curves reflect the fluidity and non-linear nature of process, with the sizes reducing as one adjusts to living with and beyond cancer.

Figures 2a, 2b and 2c. Examples of the models used in the personalised prehabilitation programme for psychological wellbeing, nutrition and physical activity.

For peer review only

For peer review only - http://bmjopen.bmj.com/site/about/guidelines.xhtml

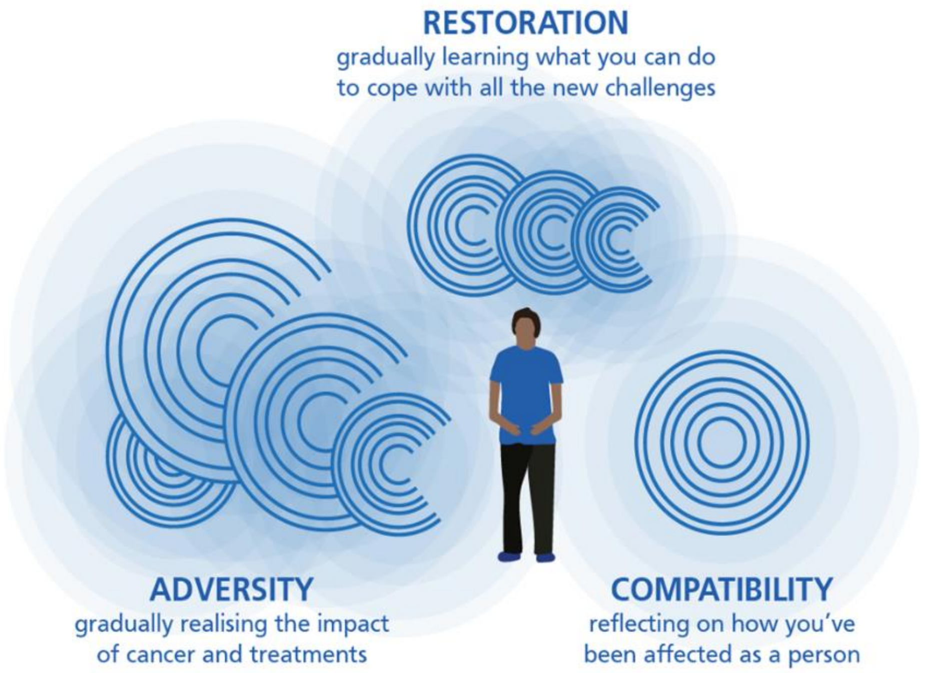

Figure 1. Diagrammatic representation of the Adversity, Restoration and Compatibility (ARC) model of the experience of adjusting to living with and beyond cancer. The waves and curves reflect the fluidity and non-linear nature of process, with the sizes reducing as one adjusts to living with and beyond cancer.

For peer review only

2a) Psychological wellbeing

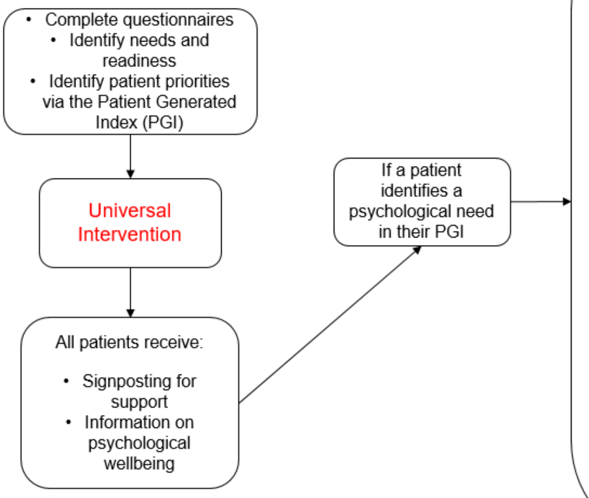

Consider / propose:	LOW NEED<br><5 in any domain on the emotions thermometer<br><4 in need for help on the emotions thermometer	HIGH NEED<br>>5 in any domain on the emotions thermometer<br>>4 in need for help on the emotions thermometer
**LOW READINESS**<br>Patient Activation Measure (PAM) 1-2<br>Cancer Behaviour Inventory (CBI) <50%	Universal Intervention<br>❑ Reinforce one existing psychological wellbeing activity the patient already does	Targeted Intervention – Professional<br>❑ Clinical Nurse Specialist psychological assessment<br>❑ Macmillan Cancer Support care line<br>❑ Referral to the psycho-oncology team if mental health history / risk
**HIGH READINESS**<br>Patient Activation Measure (PAM) 3-4<br>Cancer Behaviour Inventory (CBI) >50%	Targeted Intervention – Self-management<br>❑ Specific group or activity e.g. Maggies (or other)<br>❑ Try an NHS approved app e.g. www.nhs.uk/apps-library/worrytree/	Specialist Intervention<br>❑ Refer to the psycho-oncology team

2b) Nutrition

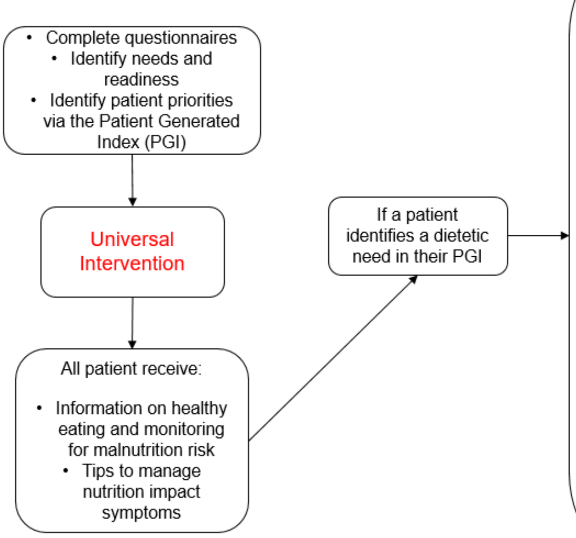

Consider / propose:	LOW NEED<br>Patient Generated Subjective Global Assessment (PG-SGA) <4	HIGH NEED<br>Patient Generated Subjective Global Assessment (PG-SGA) >4<br>>5-10% weight loss over 3-6 months<br>Handgrip < 27kg men, <16kg women
**LOW READINESS**<br>Patient Activation Measure (PAM) 1-2<br>Cancer Behaviour Inventory (CBI) <50%	Universal Intervention<br>❑ Reinforce one existing nutritional benefit that the patient already does	Targeted Intervention – Professional<br>❑ Targeted dietary tips and handouts from the Dietitian<br>❑ Refer to the Dietitian if high risk; PG-SGA > 9
**HIGH READINESS**<br>Patient Activation Measure (PAM) 3-4<br>Cancer Behaviour Inventory (CBI) >50%	Targeted Intervention – Self-management<br>❑ Specific group or activity e.g. Maggies (or other)<br>❑ Monitor weight and nutrition impact symptoms	Specialist Intervention<br>❑ Refer to the Dietitian for a 1:1 consultation

2c) Physical activity

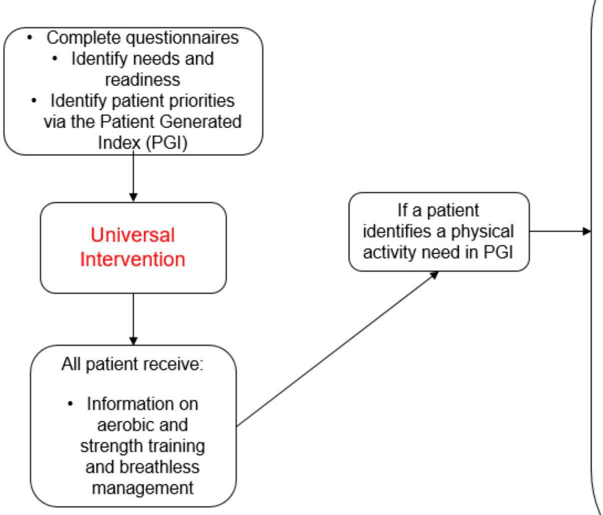

Consider / propose:	LOW NEED<br>>14 on the modified Godin Leisure Time Exercise questionnaire<br>>400m 6 minute walk test	HIGH NEED<br><14 on the modified Godin Leisure Time Exercise questionnaire<br><400m 6 minute walk test
**LOW READINESS**<br>Patient Activation Measure (PAM) 1-2<br>Cancer Behaviour Inventory (CBI) <50%	Universal Intervention<br>❑ Reinforce one existing physical activity the patient already does e.g. housework/walking	Targeted Intervention – Professional<br>❑ Can rehab<br>❑ Exercise on referral e.g. Healthwise in Hammersmith & Fulham
**HIGH READINESS**<br>Patient Activation Measure (PAM) 3-4<br>Cancer Behaviour Inventory (CBI) >50%	Targeted Intervention – Self-management<br>❑ Specific group or activity e.g. Maggies, move against cancer, Move More, public health e.g. walking groups<br>❑ Try an NHS approved app e.g. for CRF: https://untire.me/	Specialist Intervention<br>❑ Refer to a registered physiotherapist for a 1:1 consultation

Figures 2a, 2b and 2c. Examples of the models used in the personalised prehabilitation programme for psychological wellbeing, nutrition and physical activity.

Co-design approach used for the development of the personalised prehabilitation programme.

A participatory action research approach using qualitative group interviews to develop the intervention, as guided by the MRC framework was undertaken in partnership with a single London teaching hospital and Maggie's cancer centre (an independent charity that provides free cancer support, workshops and courses to help anyone affected by cancer).

Participants who had completed oncological treatment for lung cancer were invited to an informal face-to-face group discussion. Participants were invited either in person at the monthly lung cancer support group held at Maggie's cancer centre or by their clinical oncologist or clinical nurse specialist at a follow up outpatient appointment.

Nine participants were invited to the informal group discussion and a total of six participants attended. The main reason for non-attendance was a lack of time. Participants were invited to share their thoughts on what they would want from a prehabilitation programme and through facilitated discussion, participant priorities were agreed. Many participants remarked on the importance of individualisation, being seeing face-to-face and the programme being endorsed by all members of the team.

Separately, five healthcare professionals from a range of different specialties including clinical psychology, physiotherapy, dietetics and cancer services met to discuss their ideas about what a prehabilitation programme for people with lung cancer undergoing chemotherapy or radiotherapy treatment should look like.

A joint meeting was then held with both participants and healthcare professionals to share thoughts and ideas and an agreement was sought on what an ideal prehabilitation programme should look like.

Table 2 | CONSORT checklist of information to include when reporting a pilot trial

Section/topic and item No	Standard checklist item	Extension for pilot trials	Page No where item is reported
**Title and abstract**			
1a	Identification as a randomised trial in the title	Identification as a pilot or feasibility randomised trial in the title	Page 1
1b	Structured summary of trial design, methods, results, and conclusions (for specific guidance see CONSORT for abstracts)	Structured summary of pilot trial design, methods, results, and conclusions (for specific guidance see CONSORT abstract extension for pilot trials)	Page 1
**Introduction**			
Background and objectives:			
2a	Scientific background and explanation of rationale	Scientific background and explanation of rationale for future definitive trial, and reasons for randomised pilot trial	Pages 2-3
2b	Specific objectives or hypotheses	Specific objectives or research questions for pilot trial	Page 3
**Methods**			
Trial design:			
3a	Description of trial design (such as parallel, factorial) including allocation ratio	Description of pilot trial design (such as parallel, factorial) including allocation ratio	Page 3
3b	Important changes to methods after trial commencement (such as eligibility criteria), with reasons	Important changes to methods after pilot trial commencement (such as eligibility criteria), with reasons	N/A
Participants:			
4a	Eligibility criteria for participants		Page 3
4b	Settings and locations where the data were collected		Page 4
4c		How participants were identified and consented	Page 4
Interventions:			
5	The interventions for each group with sufficient details to allow replication, including how and when they were actually administered		Pages 4-5
Outcomes:			
6a	Completely defined prespecified primary and secondary outcome measures, including how and when they were assessed	Completely defined prespecified assessments or measurements to address each pilot trial objective specified in 2b, including how and when they were assessed	Pages 6-7
6b	Any changes to trial outcomes after the trial commenced, with reasons	Any changes to pilot trial assessments or measurements after the pilot trial commenced, with reasons	N/A
6c		If applicable, prespecified criteria used to judge whether, or how, to proceed with future definitive trial	Page 6
Sample size:			
7a	How sample size was determined	Rationale for numbers in the pilot trial	Page 4
7b	When applicable, explanation of any interim analyses and stopping guidelines		N/A
Randomisation:			
Sequence generation:			
8a	Method used to generate the random allocation sequence		N/A
8b	Type of randomisation; details of any restriction (such as blocking and block size)	Type of randomisation(s); details of any restriction (such as blocking and block size)	N/A
Allocation concealment mechanism:			
9	Mechanism used to implement the random allocation sequence (such as sequentially numbered containers), describing any steps taken to conceal the sequence until interventions were assigned		N/A
Implementation:			
10	Who generated the random allocation sequence, enrolled participants, and assigned participants to interventions		N/A
Blinding:			
11a	If done, who was blinded after assignment to interventions (eg, participants, care providers, those assessing outcomes) and how		N/A
11b	If relevant, description of the similarity of interventions		N/A
Analytical methods:			
12a	Statistical methods used to compare groups for primary and secondary outcomes	Methods used to address each pilot trial objective whether qualitative or quantitative	Pages 8-9
12b	Methods for additional analyses, such as subgroup analyses and adjusted analyses	Not applicable	N/A

Table 2 | CONSORT checklist of information to include when reporting a pilot trial

Section/topic and item No	Standard checklist item	Extension for pilot trials	Page No where item is reported
**Results**			
Participant flow (a diagram is strongly recommended):			
13a	For each group, the numbers of participants who were randomly assigned, received intended treatment, and were analysed for the primary outcome	For each group, the numbers of participants who were approached and/or assessed for eligibility, randomly assigned, received intended treatment, and were assessed for each objective	N/A – protocol paper
13b	For each group, losses and exclusions after randomisation, together with reasons		N/A – protocol paper
Recruitment:			
14a	Dates defining the periods of recruitment and follow-up		Page 4
14b	Why the trial ended or was stopped	Why the pilot trial ended or was stopped	N/A
Baseline data:			
15	A table showing baseline demographic and clinical characteristics for each group		N/A – protocol paper
Numbers analysed:			
16	For each group, number of participants (denominator) included in each analysis and whether the analysis was by original assigned groups	For each objective, number of participants (denominator) included in each analysis. If relevant, these numbers should be by randomised group	N/A – protocol paper
Outcomes and estimation:			
17a	For each primary and secondary outcome, results for each group, and the estimated effect size and its precision (such as 95% confidence interval)	For each objective, results including expressions of uncertainty (such as 95% confidence interval) for any estimates. If relevant, these results should be by randomised group	N/A – protocol paper
17b	For binary outcomes, presentation of both absolute and relative effect sizes is recommended	Not applicable	N/A
Ancillary analyses:			
18	Results of any other analyses performed, including subgroup analyses and adjusted analyses, distinguishing prespecified from exploratory	Results of any other analyses performed that could be used to inform the future definitive trial	N/A – protocol paper
Harms:			
19	All important harms or unintended effects in each group (for specific guidance see CONSORT for harms)		N/A – protocol paper
19a		If relevant, other important unintended consequences	N/A – protocol paper
**Discussion**			
Limitations:			
20	Trial limitations, addressing sources of potential bias, imprecision, and, if relevant, multiplicity of analyses	Pilot trial limitations, addressing sources of potential bias and remaining uncertainty about feasibility	Page 9
Generalisability:			
21	Generalisability (external validity, applicability) of the trial findings	Generalisability (applicability) of pilot trial methods and findings to future definitive trial and other studies	N/A – protocol paper
Interpretation:			
22	Interpretation consistent with results, balancing benefits and harms, and considering other relevant evidence	Interpretation consistent with pilot trial objectives and findings, balancing potential benefits and harms, and considering other relevant evidence	N/A – protocol paper
22a		Implications for progression from pilot to future definitive trial, including any proposed amendments	N/A – protocol paper
**Other information**			
Registration:			
23	Registration number and name of trial registry	Registration number for pilot trial and name of trial registry	Page 9
Protocol:			
24	Where the full trial protocol can be accessed, if available	Where the pilot trial protocol can be accessed, if available	N/A
Funding:			
25	Sources of funding and other support (such as supply of drugs), role of funders		Page 10
26		Ethical approval or approval by research review committee, confirmed with reference number	Page 9

SPIRIT

STANDARD PROTOCOL ITEMS: RECOMMENDATIONS FOR INTERVENTIONAL TRIALS

SPIRIT 2013 Checklist: Recommended items to address in a clinical trial protocol and related documents*

Section/item	Item No	Description	Page / line number in main document
**Administrative information**			
Title	1	Descriptive title identifying the study design, population, interventions, and, if applicable, trial acronym	Page 1
Trial registration	2a	Trial identifier and registry name. If not yet registered, name of intended registry	Page 9
	2b	All items from the World Health Organization Trial Registration Data Set	N/A
Protocol version	3	Date and version identifier	Page 1
Funding	4	Sources and types of financial, material, and other support	Page 10
Roles and responsibilities	5a	Names, affiliations, and roles of protocol contributors	Pages 1 and 9
	5b	Name and contact information for the trial sponsor	N/A
	5c	Role of study sponsor and funders, if any, in study design; collection, management, analysis, and interpretation of data; writing of the report; and the decision to submit the report for publication, including whether they will have ultimate authority over any of these activities	N/A
	5d	Composition, roles, and responsibilities of the coordinating centre, steering committee, endpoint adjudication committee, data management team, and other individuals or groups overseeing the trial, if applicable (see Item 21a for data monitoring committee)	N/A

For peer review only

Introduction

Background and rationale	6a	Description of research question and justification for undertaking the trial, including summary of relevant studies (published and unpublished) examining benefits and harms for each intervention	Pages 2-3
	6b	Explanation for choice of comparators	N/A
Objectives	7	Specific objectives or hypotheses	Page 3
Trial design	8	Description of trial design including type of trial (eg, parallel group, crossover, factorial, single group), allocation ratio, and framework (eg, superiority, equivalence, noninferiority, exploratory)	Page 3

Methods: Participants, interventions, and outcomes

Study setting	9	Description of study settings (eg, community clinic, academic hospital) and list of countries where data will be collected. Reference to where list of study sites can be obtained	Page 4
Eligibility criteria	10	Inclusion and exclusion criteria for participants. If applicable, eligibility criteria for study centres and individuals who will perform the interventions (eg, surgeons, psychotherapists)	Page 3
Interventions	11a	Interventions for each group with sufficient detail to allow replication, including how and when they will be administered	Pages 4-5
	11b	Criteria for discontinuing or modifying allocated interventions for a given trial participant (eg, drug dose change in response to harms, participant request, or improving/worsening disease)	See page 3 of the participant information sheet at the end of this checklist
	11c	Strategies to improve adherence to intervention protocols, and any procedures for monitoring adherence (eg, drug tablet return, laboratory tests)	See page 7 of the participant information sheet at the end of this checklist
	11d	Relevant concomitant care and interventions that are permitted or prohibited during the trial	N/A

For peer review only - http://bmjopen.bmj.com/site/about/guidelines.xhtml

Outcomes	12	Primary, secondary, and other outcomes, including the specific measurement variable (eg, systolic blood pressure), analysis metric (eg, change from baseline, final value, time to event), method of aggregation (eg, median, proportion), and time point for each outcome. Explanation of the clinical relevance of chosen efficacy and harm outcomes is strongly recommended	Pages 6-7
Participant timeline	13	Time schedule of enrolment, interventions (including any run-ins and washouts), assessments, and visits for participants. A schematic diagram is highly recommended (see Figure)	See page 6 of the participant information sheet at the end of this checklist
Sample size	14	Estimated number of participants needed to achieve study objectives and how it was determined, including clinical and statistical assumptions supporting any sample size calculations	Page 3
Recruitment	15	Strategies for achieving adequate participant enrolment to reach target sample size	Page 3

Methods: Assignment of interventions (for controlled trials)

Allocation:

Sequence generation	16a	Method of generating the allocation sequence (eg, computer-generated random numbers), and list of any factors for stratification. To reduce predictability of a random sequence, details of any planned restriction (eg, blocking) should be provided in a separate document that is unavailable to those who enrol participants or assign interventions	N/A
Allocation concealment mechanism	16b	Mechanism of implementing the allocation sequence (eg, central telephone; sequentially numbered, opaque, sealed envelopes), describing any steps to conceal the sequence until interventions are assigned	N/A

Implementation	16c	Who will generate the allocation sequence, who will enrol participants, and who will assign participants to interventions	N/A
Blinding (masking)	17a	Who will be blinded after assignment to interventions (eg, trial participants, care providers, outcome assessors, data analysts), and how	N/A
	17b	If blinded, circumstances under which unblinding is permissible, and procedure for revealing a participant's allocated intervention during the trial	N/A
**Methods: Data collection, management, and analysis**			
Data collection methods	18a	Plans for assessment and collection of outcome, baseline, and other trial data, including any related processes to promote data quality (eg, duplicate measurements, training of assessors) and a description of study instruments (eg, questionnaires, laboratory tests) along with their reliability and validity, if known. Reference to where data collection forms can be found, if not in the protocol	Pages 4-8
	18b	Plans to promote participant retention and complete follow-up, including list of any outcome data to be collected for participants who discontinue or deviate from intervention protocols	See page 3 of the participant information sheet at the end of this checklist
Data management	19	Plans for data entry, coding, security, and storage, including any related processes to promote data quality (eg, double data entry; range checks for data values). Reference to where details of data management procedures can be found, if not in the protocol	Page 8 and page 3 of the participant information sheet at the end of this checklist
Statistical methods	20a	Statistical methods for analysing primary and secondary outcomes. Reference to where other details of the statistical analysis plan can be found, if not in the protocol	Page 8

	20b	Methods for any additional analyses (eg, subgroup and adjusted analyses)	N/A
	20c	Definition of analysis population relating to protocol non-adherence (eg, as randomised analysis), and any statistical methods to handle missing data (eg, multiple imputation)	Pages 8-9
**Methods: Monitoring**			
Data monitoring	21a	Composition of data monitoring committee (DMC); summary of its role and reporting structure; statement of whether it is independent from the sponsor and competing interests; and reference to where further details about its charter can be found, if not in the protocol. Alternatively, an explanation of why a DMC is not needed	A DMC is not needed as this is a small, short-term feasibility pilot study with low risk
	21b	Description of any interim analyses and stopping guidelines, including who will have access to these interim results and make the final decision to terminate the trial	N/A
Harms	22	Plans for collecting, assessing, reporting, and managing solicited and spontaneously reported adverse events and other unintended effects of trial interventions or trial conduct	See reporting of adverse events from study protocol at the end of this checklist
Auditing	23	Frequency and procedures for auditing trial conduct, if any, and whether the process will be independent from investigators and the sponsor	N/A
**Ethics and dissemination**			
Research ethics approval	24	Plans for seeking research ethics committee/institutional review board (REC/IRB) approval	Page 9
Protocol amendments	25	Plans for communicating important protocol modifications (eg, changes to eligibility criteria, outcomes, analyses) to relevant parties (eg, investigators, REC/IRBs, trial participants, trial registries, journals, regulators)	Page 9

Consent or assent	26a	Who will obtain informed consent or assent from potential trial participants or authorised surrogates, and how (see Item 32)	The lead researcher. An example of the participant consent form can be found at the end of this checklist
	26b	Additional consent provisions for collection and use of participant data and biological specimens in ancillary studies, if applicable	N/A
Confidentiality	27	How personal information about potential and enrolled participants will be collected, shared, and maintained in order to protect confidentiality before, during, and after the trial	See pages 3-4 of the participant information sheet at the end of this checklist
Declaration of interests	28	Financial and other competing interests for principal investigators for the overall trial and each study site	Page 10
Access to data	29	Statement of who will have access to the final trial dataset, and disclosure of contractual agreements that limit such access for investigators	See page 3 of the participant information sheet at the end of this checklist
Ancillary and post-trial care	30	Provisions, if any, for ancillary and post-trial care, and for compensation to those who suffer harm from trial participation	N/A
Dissemination policy	31a	Plans for investigators and sponsor to communicate trial results to participants, healthcare professionals, the public, and other relevant groups (eg, via publication, reporting in results databases, or other data sharing arrangements), including any publication restrictions	Page 9
	31b	Authorship eligibility guidelines and any intended use of professional writers	N/A
	31c	Plans, if any, for granting public access to the full protocol, participant-level dataset, and statistical code	N/A

Appendices

For peer review only

Informed consent materials	32	Model consent form and other related documentation given to participants and authorised surrogates	See participant information sheet and example of the participant consent form at the end of this checklist
Biological specimens	33	Plans for collection, laboratory evaluation, and storage of biological specimens for genetic or molecular analysis in the current trial and for future use in ancillary studies, if applicable	N/A

*It is strongly recommended that this checklist be read in conjunction with the SPIRIT 2013 Explanation & Elaboration for important clarification on the items. Amendments to the protocol should be tracked and dated. The SPIRIT checklist is copyrighted by the SPIRIT Group under the Creative Commons "Attribution-NonCommercial-NoDerivs 3.0 Unported" license.

NHS
Imperial College Healthcare
NHS Trust

Kelly Wade-Mcbane

Imperial-lung-prehab@nhs.net

PATIENT INFORMATION SHEET

Study title

A personalised plan of diet, exercise and emotional support for patients having chemotherapy, radiotherapy or immunotherapy treatment for lung cancer.

Reviewed by: The London Bridge Research Ethics Committee

Invitation

We would like to invite you to take part in our research study. Before you decide, it is important that you understand why the research is being done and what it will involve. Please take time to read and consider this information. If anything is not clear, please ask one of the research team.

Purpose of the study

The term 'prehabilitation' refers to a period of time before treatment and often includes a combination of diet, exercise and/or wellbeing plans to help patients through their cancer treatment. The benefits of prehabilitation have been shown in patients having surgery, but there is little research into its use for patients having chemotherapy, radiotherapy or immunotherapy treatment.

This study will see if a personalised plan of diet, exercise and emotional support can be used in practice for patients having chemotherapy, radiotherapy or immunotherapy treatment for lung cancer.

This study is part of the researcher's educational higher degree.

Why have I been invited?

You have been invited to take part as you have been diagnosed with lung cancer and are about to start chemotherapy, radiotherapy or immunotherapy treatment.

Do I have to take part?

No, you do not have to take part. Your choice to take part in the study is entirely voluntary. To help with the development of the service, a member of your clinical team may ask you why you have decided not to participate. This is entirely optional and you do not have to provide a reason. Your decision about the research will not affect the medical care you receive.

For peer review only

What will happen to me if I decide to take part?

Firstly, you will be asked to provide written consent either in person or online using your personal computer. Following consent, you will be asked to complete five questionnaires on wellbeing, physical activity and diet. These questionnaires can be completed either on paper or online. These questionnaires will take approximately 30 minutes to complete.

You will then be invited to a face to face appointment which will last approximately one hour at Charing Cross Hospital. This appointment will be with the lead researcher, who is a specialist dietitian at Imperial College Healthcare NHS Trust. At this appointment, we will:

- Review your questionnaires
- Ask you to complete some further questionnaires related to quality of life, diet and activity
- Do an assessment of your walking. This will involve you walking at your own pace as far as you can in 6 minutes
- Measure your grip strength

We will then agree your personalised plan, taking into account your level of need and readiness; identified from the questionnaires and the goals you want to achieve. If your personalised plan identifies an emotional, diet or activity need, you will receive an intervention that is suited to you.

After this appointment, we will ask you to keep a daily diary to rate your symptoms, appetite, mobility and mood throughout your treatment by providing a score each day using a scale from 0-10. You can use this diary when we phone you each week to let us know how you are getting on and to talk about any days you have found particularly good or bad.

We will invite you for two further face to face appointments at the week three and week six of your chemotherapy, radiotherapy or immunotherapy treatment. At these appointments, we will repeat the questionnaires, assess your walking and measure your handgrip strength. Personalised goals will be agreed at each stage.

Please see the diagram on page six for further information.

What are the possible benefits of taking part?

We do not know if there will be any individual benefits, but the information we get might help inform future research to help improve the lives of lung cancer patients.

Are there any possible disadvantages or risks from taking part?

There are no risks from taking part in this study.

You will be asked to complete a walking test, questionnaires and keep a daily diary, which could be a burden.
The walking test involves you walking at your own pace over a 6 minute period. You can stop when you need to. Physical activity has shown to be safe in lung cancer patients and the walking test is widely used in cancer research.

For peer review only

The questionnaires are quick to complete (about 30 minutes) and will be used to personalise your care.

The daily diary is simple to use. You will be asked to provide a score each day using a scale to rate your concerns. Please see page seven for an example of the daily diary.

All research appointments will be scheduled for the same days as your clinical appointments to prevent you having to make additional trips to the hospital. Your clinical visits may be extended by approximately one hour for the research.

Will my General Practitioner (GP) be informed of my participation?

This is optional and will not affect your participation in the study. We can let your GP know if you wish and you can tell us about this in the consent form. If you wish for your GP to be informed, we will send them a written letter accompanied with a study information sheet.

Will I be reimbursed for taking part?

Yes, we offer up to £10 towards the cost of your travel if you are required to come into hospital on a day outside your usual clinical visits. Please retain your receipts. We will provide you with a travel expenses form. Once completed, you can take this to the cashier’s desk at Charing Cross Hospital to be reimbursed.

What will happen if I don't want to carry on with the study?

Participation in the study is entirely voluntary and you can withdraw at any point. Withdrawal will not affect any medical care that you are receiving. If you no longer want to carry on with the research study, please email us at Imperial-lung-prehab@nhs.net. We will retain and use the information collected up to that point, but we will not collect any further information.

What happens at the end of the study?

If the results are positive, we will plan to do more testing and offer this to more people. We will provide a written summary of the findings to all participants. We will share the results in scientific journals and conferences, so that others can learn from this as well. Patients will not be identifiable from any report or publication.

Will my taking part in the study be kept confidential and what will happen to my data?

To ensure the confidentiality of all personal data, all members of the research team will follow the Imperial College Healthcare NHS Trust confidentiality policy. Any identifiable data gathered will be stored securely in accordance with the Trust policy on either a password protected Trust computer or under lock and key.

You will be given a unique study number that will be used from the start of the study. Files such as paper consent forms and questionnaires will be stored in research offices located within the clinical areas and will be secured either by lock and key or key code and accessible by only departmental staff or the Trust’s security team. All paper documentation kept in these will be locked.

For peer review only

Your data will not be shared with anyone outside of your hospital treating team or local research team. Any data containing any identifiable information will only be available to the chief investigator and appropriate members of the study team on a delegation log. Your medical record will not be removed from the clinical areas.

All data will be collected according to the Data Protection Act (2018) and in line with General Data Protection Regulations (GDPR).

If you lose capacity to consent during the study, you will be withdrawn from the study. Identifiable data already collected with consent would be retained and used in the study. No further data would be collected or any other research procedures carried out on or in relation to you.

Once we have finished the study, we will keep some of the data so that we can check the results. We will write our reports in a way that no-one can work out that you took part in the study. You can find out more about how we use your information by contacting the research team at Imperial-lung-prehab@nhs.net.

How will we use information about you?

We will need to use information from you and from your medical records for this research project.

This information will include name, contact number, date of birth, contact details and proposed treatment plan held by the site and/or sponsor for the research. People will use this information to do the research or to check your records to make sure that the research is being done properly.

People who do not need to know who you are will not be able to see your name or contact details. Your data will have a code number instead.

We will keep all information about you safe and secure.

Once we have finished the study, we will keep some of the data so we can check the results. We will write our reports in a way that no-one can work out that you took part in the study.

What are your choices about how your information is used?

You can stop being part of the study at any time, without giving a reason, but we will keep information about you that we already have.

We need to manage your records in specific ways for the research to be reliable. This means that we won't be able to let you see or change the data we hold about you.

If you agree to take part in this study, you will have the option to take part in future research using your data saved from this study.

How do I find out more about how my information is used?

You can find out more about how we use your information at www.hra.nhs.uk/information-about-patients/

For peer review only

Alternatively you can:

- Ask one of the research team
- Send an email to Imperial-lung-prehab@nhs.net.
- Call us on 0203 311 7135
- Contact the Imperial Academic Health Science Centre Data Protection Officer by calling 020 7594 1862

What if we find something unexpected?

If anything of concern regarding your health comes to light during the study, your clinical team and GP will be notified.

What if something goes wrong?

Imperial College Healthcare NHS Trust holds standard NHS Hospital indemnity and insurance cover with NHS resolution for NHS Trusts in England, which apply to this study. This does not affect your legal rights to seek compensation.

If you are harmed due to someone's negligence, then you may have grounds for a legal action. Regardless of this, if you wish to complain, or have any concerns about any aspect of the way you have been treated during the course of this study then you should immediately inform the Investigator.

The normal National Health Service complaints mechanisms are also available to you. If you are still not satisfied with the response, you may contact the Imperial Academic Health Science Centre Research Governance and Integrity Team.

What if there is a problem?

If you wish to make a complaint or if you have any concerns about any aspect of the way you have been approached or treated during the course of this study, please contact the research team at Imperial-lung-prehab@nhs.net.
You can also contact the Patient Advice and Liaison Service (PALS) on 020 331 27777.
If you are still not satisfied with the response, you may contact the Imperial Academic Health Science Centre Research Governance and Integrity Team on 020 7594 1862.

Who has reviewed the study?

All research in the NHS is looked at by an independent group of people, called a Research Ethics Committee, to protect participants' interests. This study has been reviewed and given favourable ethical opinion for conduct in the NHS by The London Bridge Research Ethics Committee.

Thank you for considering to take part in this study

For peer review only

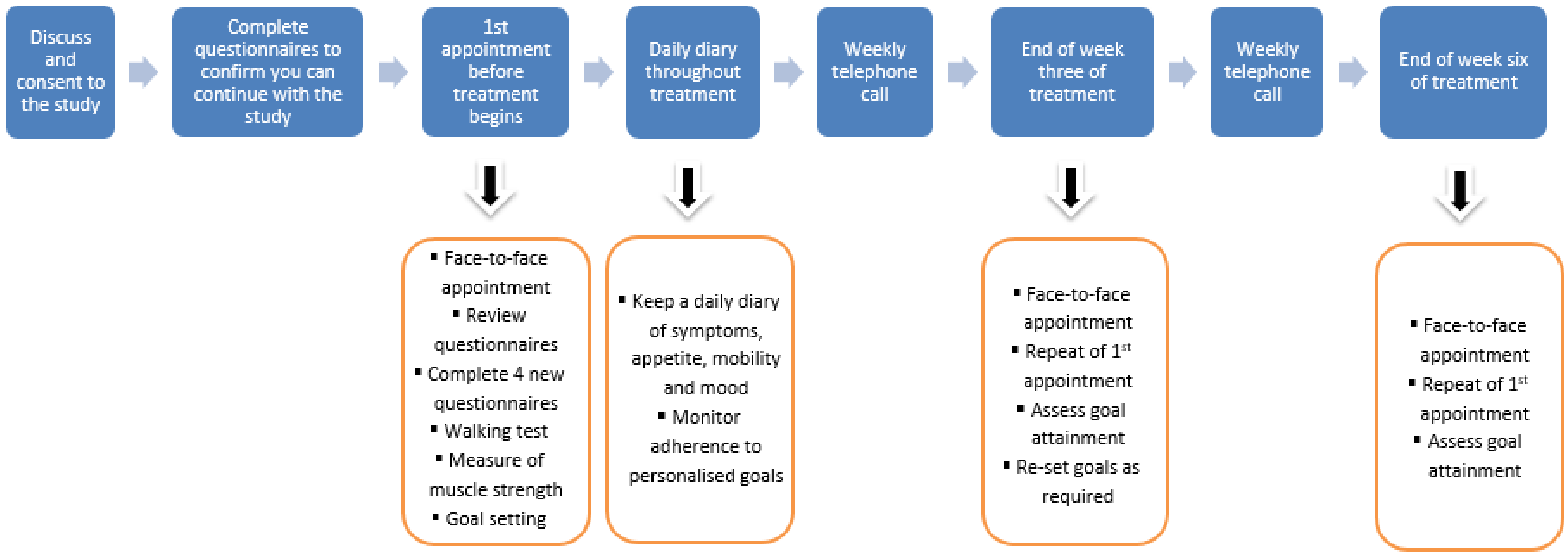
Discuss and consent to the study
Complete questionnaires to confirm you can continue with the study
1st appointment before treatment begins
Daily diary throughout treatment
Weekly telephone call
End of week three of treatment
Weekly telephone call
End of week six of treatment
▪ Face-to-face appointment ▪ Review questionnaires ▪ Complete 4 new questionnaires ▪ Walking test ▪ Measure of muscle strength ▪ Goal setting
▪ Keep a daily diary of symptoms, appetite, mobility and mood ▪ Monitor adherence to personalised goals
▪ Face-to-face appointment ▪ Repeat of 1st appointment ▪ Assess goal attainment ▪ Re-set goals as required
▪ Face-to-face appointment ▪ Repeat of 1st appointment ▪ Assess goal attainment

DIARY

Using this scale from 0-10 with 0 being no concerns and 10 being the worst it has been, please rate your symptoms, appetite, mobility and level of distress each day.

0	1	2	3	4	5	6	7	8	9	10

Cycle 1

	Monday	Tuesday	Wednesday	Thursday	Friday	Saturday	Sunday
Symptoms							
Appetite							
Mobility							
Distress							

At the end of the week, please tell us about any days you found particularly good or difficult. Highlight these and we will talk about them.

NHS
Imperial College Healthcare
NHS Trust

Kelly Wade-Mcbane

Imperial-lung-prehab@nhs.net

Participant identification number | | | | | |

CONSENT FORM

Study Title

A personalised prehabilitation model for patients undergoing chemotherapy, radiotherapy or immunotherapy treatment for lung cancer: A feasibility pilot study.

Name of Researcher: Kelly Wade-Mcbane

If you agree, please initial the box

1. I confirm that I have received a copy of the patient information sheet (version 7, 06/01/2023) for this study. I have read and considered the information and have had the opportunity to ask questions and have had these answered satisfactorily	
2. I understand that my participation is voluntary. I am free to withdraw at any time without having to explain and my medical care or legal rights will not be affected	
3. I understand that relevant sections of my medical data may be looked at by responsible individuals from Imperial College Healthcare NHS Trust or by regulatory authorities where it is relevant to my taking part in this research. I give permission for these individuals to have access to my records	
4. I agree / do not agree (delete as applicable) to my general practitioner (GP) being informed of my participation in the study. This is optional and will not affect your participation in the study	
5. I understand that in any report or publication on the results of this study, my identity will remain anonymous	
6. I give / do not give (delete as applicable) consent for information collected about me to be used to support other research in the future. This is optional and will not affect your participation in the study	
7. I agree to take part in this study	

_________________________ _______________ _________________________

Name of participant Date Signature

_________________________ _______________ _________________________

Name of person taking consent Date Signature

** 1 copy for participant; 1 copy for researcher's log; 1 copy to be uploaded into patient's* electronic medical notes

For peer review only - http://bmjopen.bmj.com/site/about/guidelines.xhtml

ADVERSE EVENTS

1.1. DEFINITIONS

Adverse Event (AE): any untoward medical occurrence in a patient or clinical study subject.

Serious Adverse Event (SAE): any untoward and unexpected medical occurrence or effect that:

- **Results in death**
- **Is life-threatening** – *refers to an event in which the subject was at risk of death at the time of the event; it does not refer to an event which hypothetically might have caused death if it were more severe*
- **Requires hospitalisation, or prolongation of existing inpatients' hospitalisation**
- **Results in persistent or significant disability or incapacity**
- **Is a congenital anomaly or birth defect**

Medical judgement should be exercised in deciding whether an AE is serious in other situations. Important AEs that are not immediately life-threatening or do not result in death or hospitalisation but may jeopardise the subject or may require intervention to prevent one of the other outcomes listed in the definition above, should also be considered serious.

1.2. REPORTING PROCEDURES

All adverse events should be reported. Depending on the nature of the event the reporting procedures below should be followed. Any questions concerning adverse event reporting should be directed to the Chief Investigator in the first instance.

5.3.1 Non serious AEs

All such events, whether expected or not, should be recorded- it should be specified if only some non-serious AEs will be recorded, any reporting should be consistent with the purpose of the trial end points.

5.3.2 Serious AEs

An SAE form should be completed and emailed to the Chief Investigator within 24 hours. However, relapse and death due to lung cancer, and hospitalisations for elective treatment of a pre-existing condition do not need reporting as SAEs.

All SAEs should be reported to the London Bridge Research Ethics Committee where in the opinion of the Chief Investigator, the event was:

- 'related', ie resulted from the administration of any of the research procedures; and
- 'unexpected', ie an event that is not listed in the protocol as an expected occurrence

Reports of related and unexpected SAEs should be submitted within 15 days of the Chief Investigator becoming aware of the event, using the NRES SAE form for non-IMP studies. The Chief Investigator must also notify the Sponsor of all related and unexpected SAEs.

Local investigators should report any SAEs as required by their Local Research Ethics Committee, Sponsor and/or Research & Development Office.

Contact details for reporting SAEs
RGIT@imperial.ac.uk

For peer review only - http://bmjopen.bmj.com/site/about/guidelines.xhtml
